# Associations between IL-6 and trajectories of depressive symptoms across the life course: Evidence from ALSPAC and UK Biobank cohorts

**DOI:** 10.1192/j.eurpsy.2025.7

**Published:** 2025-01-27

**Authors:** Amelia J. Edmondson-Stait, Ella Davyson, Xueyi Shen, Mark James Adams, Golam M. Khandaker, Veronique E. Miron, Andrew M. McIntosh, Stephen M. Lawrie, Alex S.F. Kwong, Heather C. Whalley

**Affiliations:** 1 Translational Neuroscience PhD Programme, Centre for Clinical Brain Sciences, University of Edinburgh, Edinburgh, UK; 2 Centre for Clinical Brain Sciences, University of Edinburgh, Edinburgh, UK; 3 MRC Integrative Epidemiology Unit, Population Health Sciences, Bristol Medical School, University of Bristol, Bristol, UK; 4 Centre for Academic Mental Health, Population Health Sciences, Bristol Medical School, University of Bristol, Bristol, UK; 5 National Institute for Health and Care Research Bristol Biomedical Research Centre, United Hospitals Bristol and Weston NHS Foundation Trust, Bristol, UK; 6 BARLO Multiple Sclerosis Centre, Keenan Research Centre for Biomedical Science at St. Michael’s Hospital, Toronto, ON, Canada; 7 Department of Immunology, University of Toronto, Toronto, ON, Canada; 8 UK Dementia Research Institute at The University of Edinburgh, Centre for Discovery Brain Sciences, University of Edinburgh, Edinburgh, UK; 9 Generation Scotland, Institute of Genetics and Cancer, University of Edinburgh, Edinburgh, UK

**Keywords:** UK Biobank, ALSPAC, depression, trajectories, inflammation

## Abstract

**Background:**

Peripheral inflammatory markers, including serum interleukin 6 (IL-6), are associated with depression, but less is known about how these markers associate with depression at different stages of the life course.

**Methods:**

We examined the associations between serum IL-6 levels at baseline and subsequent depression symptom trajectories in two longitudinal cohorts: ALSPAC (age 10–28 years; *N* = 4,835) and UK Biobank (39–86 years; *N* = 39,613) using multilevel growth curve modeling. Models were adjusted for sex, BMI, and socioeconomic factors. Depressive symptoms were measured using the Short Moods and Feelings Questionnaire in ALSPAC (max time points = 11) and the Patient Health Questionnaire-2 in UK Biobank (max time points = 8).

**Results:**

Higher baseline IL-6 was associated with worse depression symptom trajectories in both cohorts (largest effect size: 0.046 [ALSPAC, age 16 years]). These associations were stronger in the younger ALSPAC cohort, where additionally higher IL-6 levels at age 9 years was associated with worse depression symptoms trajectories in females compared to males. Weaker sex differences were observed in the older cohort, UK Biobank. However, statistically significant associations (pFDR <0.05) were of smaller effect sizes, typical of large cohort studies.

**Conclusions:**

These findings suggest that systemic inflammation may influence the severity and course of depressive symptoms across the life course, which is apparent regardless of age and differences in measures and number of time points between these large, population-based cohorts.

## Introduction

There is substantial evidence to suggest low-grade inflammation, as reflected by elevated levels of circulating inflammatory markers, such as C-reactive protein (CRP) and a cytokine interleukin 6 (IL-6), in the blood and cerebrospinal fluid, may contribute to the etiology of depression [1–4]. Neuroimaging and postmortem brain studies have shown increased markers of neuroinflammation in individuals with depression compared to controls [4–6]. Furthermore, peripheral inflammatory markers have been shown to associate with changes in brain structure in both observational [7, 8] and Mendelian randomization (MR) [9] studies, suggesting a potential mechanism by which inflammation may have a role in depression. Longitudinal studies have shown increased blood IL-6, but not CRP, levels in childhood associate with depressive and psychotic symptoms in early adulthood [10–12]. Increased inflammatory markers have been also shown to associate with worse depressive symptom severity, including in an MR study that found a potential causal association of IL-6 with suicidal thoughts [13, 14]. Causal evidence also comes from RCTs showing anti-inflammatory treatment for chronic inflammatory conditions improves depressive symptoms independent of improvement in physical symptoms and other MR studies suggesting putative causality of IL-6 on major depressive disorder [15, 16]. Other clinical trials on anti-inflammatory agents as adjunctive treatment for depression have resulted in mixed results [17–20], with effective treatment outcomes more commonly found when stratifying by baseline inflammatory markers [18, 19]. Furthermore, there is some pilot evidence to suggest such stratification may be more pertinent in females compared to males [21].

Depression affects individuals across the entire life course with an onset typically occurring between ages 20 and 30 years [22, 23]. However, there are few studies investigating the effect of baseline inflammation on the longitudinal patterns of depressive symptoms over the life course. A study using latent class analysis in the ALSPAC cohort showed that serum IL-6 levels at age 9 years associated with a trajectory group of persistently worse depressive symptoms from ages 10 to 19 years [24]. A study also using latent class analysis in The Netherlands Study of Depression and Anxiety cohort (age 18–65 years at baseline) found increased inflammatory blood markers associated with an atypical depression subgroup [25]. Over a 6-year follow-up, this subgroup had higher BMI and rate of metabolic syndrome compared to a melancholic depression subgroup and controls [26]. Similar findings of increased prevalence of metabolic syndrome in an atypical depression latent class were also found in older individuals (aged 60 years or older, *N* = 510) in The Netherlands Study of Depression in Older persons cohort [27]. However, none of these studies directly examine the effects of inflammation over a larger period of the life course.

Examining the effects of inflammation on depression across the life course provides insight into the heterogeneity and underlying mechanisms of depression at specific developmental stages, aiding the development of biologically based stratification. Depression is highly heterogeneous and there is increasing cross-sectional evidence that an inflammatory subgroup of depression exists, associated with worse depressive symptom severity [13, 28]. Therefore, it is crucial to examine whether increased inflammation associates with increased depression symptom severity over different stages of the life course. One such method for understanding the longitudinal relationships between inflammation and depression is trajectory analysis [29]. Briefly, this method assesses the patterns of change in depressive symptoms over time (trajectories) for individuals or groups of individuals from repeated assessments of depression symptoms [29]. This then facilitates the investigation into risk factors that may influence the course of these trajectories and whether these effects persist over time.

Additionally, it is known there is a sex difference in both depression and inflammation [29–32]. Evidence suggests these sex differences extend into differences in inflammatory-associated depression, especially in adolescence but with inconsistent findings in later life [33–36]. There is a need to understand the sex differences more fully at different stages of the life course, while assessing repeated measures of subsequent depression.

Here, we used multilevel growth curve modeling to investigate the effects of IL-6 on subsequent trajectories of depressive symptoms in two longitudinal cohorts: ALSPAC (age 10–28 years) and UK Biobank (39–86 years). Due to previous observational longitudinal and MR studies showing stronger effects of serum IL-6 compared to CRP with depression, the focus of this study is on IL-6 [9–12, 16]. Specifically, we tested whether increased baseline measures of serum IL-6 are associated with worse trajectories of depressive symptoms and if this effect is seen consistently across two different cohorts spanning early and later life. Individuals were divided into groups based on IL-6 tertiles, with the bottom third tertile group consisting of people with lower levels of IL-6 and the top third tertile group consisting of people with higher levels of IL-6, as has been studied previously [37, 38]. We also tested if there was a sex difference in the relationship between IL-6 and subsequent trajectories of depressive symptoms, by stratifying analysis by sex. Finally, due to the difficulty in interpreting the effects of polynomial trajectory models, we also calculated the mean depressive scores for each IL-6 tertile trajectory and assessed the differences in depressive scores between the top and bottom third IL-6 tertile trajectories of depressive symptoms at different ages.

## Materials and methods

### Study sample

#### ALSPAC cohort

ALSPAC is an ongoing, longitudinal, prospective, population-based study in South-West England investigating the impact of various exposures on health and developmental outcomes [39–41]. Initially, 14,541 pregnant mothers with an estimated delivery date between April 1991 and December 1992 were recruited. This resulted in 14,092 live births and 13,988 children still alive after 1 year. When the oldest children were approximately 7 years of age, an attempt was made to bolster the initial sample with eligible cases who had failed to join the study originally. The total sample size for analyses using any data collected after the age of seven is therefore 15,447 pregnancies, of which 14,901 children were alive at 1 year of age. Further details of this study cohort are described in the cohort profile publications [39–41]. Demographics of ALSPAC participants used within the current study are shown in Table 1. Number of participants at each age for each time point is shown in Supplementary Table 1.

**Table 1.** Demographic table of ALSPAC participants

	IL-6 tertile group		
	Bottom third	Middle third	Top third
n	1616	1602	1617
Sex = Males (%)	943 (58.4)	807 (50.4)	666 (41.2)
BMI (log transformed) (mean [SD])	2.81 (0.11)	2.85 (0.14)	2.90 (0.17)
Maternal education at birth (%)			
A-level/degree	710 (43.9)	646 (40.3)	636 (39.3)
CSE/O-level/vocational	753 (46.6)	789 (49.3)	780 (48.2)
NA	153 (9.5)	167 (10.4)	201 (12.4)
Currently taking any medication (%)			
FALSE	1426 (88.2)	1373 (85.7)	1341 (82.9)
TRUE	187 (11.6)	227 (14.2)	272 (16.8)
NA	3 (0.2)	2 (0.1)	4 (0.2)
Townsend deprivation index quintile (%)			
1	478 (29.6)	399 (24.9)	399 (24.7)
2	219 (13.6)	207 (12.9)	198 (12.2)
3	245 (15.2)	219 (13.7)	235 (14.5)
4	222 (13.7)	261 (16.3)	271 (16.8)
5	74 (4.6)	93 (5.8)	95 (5.9)
NA	378 (23.4)	423 (26.4)	419 (25.9)
IL–6 (inverse normal transformed, *Z*-score) (mean [SD])	−1.09 (0.53)	0.00 (0.25)	1.09 (0.53)
IL–6 (raw value, pg/ml)	0.39 (0.13)	0.82 (0.15)	2.48 (1.95)
SMFQ (time point 1) (mean [SD])	3.87 (3.32)	3.96 (3.50)	4.10 (3.55)
SMFQ (time point 2) (mean [SD])	3.79 (3.75)	3.71 (3.56)	4.26 (4.04)
SMFQ (time point 3) (mean [SD])	4.46 (4.02)	4.73 (4.42)	5.38 (4.74)
SMFQ (time point 4) (mean [SD])	5.38 (5.23)	5.54 (5.43)	6.32 (5.89)
SMFQ (time point 5) (mean [SD])	5.99 (4.90)	6.56 (5.41)	6.87 (5.40)
SMFQ (time point 6) (mean [SD])	6.12 (5.39)	6.19 (5.54)	7.36 (6.21)
SMFQ (time point 7) (mean [SD])	5.09 (4.78)	5.26 (5.26)	5.68 (5.49)
SMFQ (time point 8) (mean [SD])	5.76 (5.13)	5.79 (5.25)	6.25 (5.58)
SMFQ (time point 9) (mean [SD])	6.51 (5.74)	6.27 (5.57)	6.99 (6.01)
SMFQ (time point 10) (mean [SD])	6.19 (5.97)	6.24 (5.97)	7.12 (6.60)
SMFQ (time point 11) (mean [SD])	6.43 (5.89)	6.01 (5.72)	7.01 (6.27)
Age (time point 1) (mean [SD])	10.62 (0.24)	10.64 (0.25)	10.63 (0.24)
Age (time point 2) (mean [SD])	12.81 (0.22)	12.80 (0.22)	12.80 (0.21)
Age (time point 3) (mean [SD])	13.82 (0.19)	13.83 (0.21)	13.83 (0.21)
Age (time point 4) (mean [SD])	16.69 (0.24)	16.68 (0.25)	16.68 (0.23)
Age (time point 5) (mean [SD])	17.79 (0.38)	17.83 (0.39)	17.83 (0.39)
Age (time point 6) (mean [SD])	18.66 (0.48)	18.67 (0.49)	18.64 (0.49)
Age (time point 7) (mean [SD])	21.96 (0.50)	21.95 (0.53)	21.96 (0.51)
Age (time point 8) (mean [SD])	22.88 (0.49)	22.90 (0.53)	22.90 (0.52)
Age (time point 9) (mean [SD])	23.88 (0.48)	23.88 (0.53)	23.86 (0.51)
Age (time point 10) (mean [SD])	25.78 (0.48)	25.77 (0.52)	25.76 (0.51)
Age (time point 11) (mean [SD])	28.38 (0.52)	28.38 (0.54)	28.37 (0.53)

#### UK Biobank cohort

UK Biobank is a large, population-based, prospective study, aiming to investigate contributing factors to a wide range of health-related outcomes [42]. UK Biobank consists of over 500,000 participants, aged 39–69 years when recruited between 2006 and 2010 over 22 assessment centers throughout the UK (http://www.ukbiobank.ac.uk/).

Data collection occurred at both in-person assessment visits and remote online follow-up questionnaires. In-person assessment visits included an initial assessment visit (2006–2010), first repeat assessment visit (2012–2013), an imaging visit (2014+), and a repeat imaging visit (2019+) [42, 43]. Online follow-up questionnaires included assessments such as mental health (2016–2017), experiences of pain (2019), health and well-being (2022+), and mental well-being (2022+). Demographics of UK Biobank participants used within the current study are shown in Table 2. Number of participants at each age for each time point is shown in Supplementary Table 2.

**Table 2.** Demographic table of UK Biobank participants

	IL-6 tertile group		
	Bottom third	Middle third	Top third
n	13776	13640	12197
Sex = Male (%)	6162 (44.7)	6291 (46.1)	5737 (47.0)
BMI (log transformed) (mean [SD])	3.22 (0.13)	3.30 (0.14)	3.37 (0.17)
BMI (categorized) (%)			
Underweight	105 (0.8)	50 (0.4)	36 (0.3)
Healthy	6894 (50.0)	4033 (29.6)	2291 (18.8)
Overweight	5507 (40.0)	6563 (48.1)	4979 (40.8)
Obese	1216 (8.8)	2856 (20.9)	4351 (35.7)
Morbidly obese	18 (0.1)	98 (0.7)	461 (3.8)
Missing	36 (0.3)	40 (0.3)	79 (0.6)
Inflammatory condition = TRUE (%)	1573 (11.4)	2095 (15.4)	2674 (21.9)
Taking inflammatory medication = TRUE (%)	3138 (22.8)	3692 (27.1)	3822 (31.3)
Smoking status (%)			
Previous	4412 (32.0)	4780 (35.0)	4477 (36.7)
Current	1196 (8.7)	1352 (9.9)	1612 (13.2)
Never	8124 (59.0)	7470 (54.8)	6060 (49.7)
NA	44 (0.3)	38 (0.3)	48 (0.4)
Townsend deprivation index (mean [SD])	−1.56 (2.99)	−1.33 (3.07)	−0.90 (3.27)
IL–6 (inverse normal transformed, Z-score) (mean [SD])	−1.09 (0.53)	0.00 (0.25)	1.04 (0.50)
IL–6 (raw value, Olink normalized) (mean [SD])	−0.70 (0.30)	−0.01 (0.17)	0.97 (0.77)
PHQ–2 (initial time point) (mean [SD])	0.53 (1.03)	0.56 (1.08)	0.63 (1.16)
PHQ–2 (repeat time point) (mean [SD])	0.46 (0.96)	0.41 (0.86)	0.45 (1.00)
PHQ–2 (imaging time point) (mean [SD])	0.41 (0.96)	0.43 (0.97)	0.45 (0.95)
PHQ–2 (repeat imaging time point) (mean [SD])	0.32 (0.78)	0.46 (0.96)	0.39 (0.89)
PHQ–2 (mental health time point) (mean [SD])	0.50 (1.03)	0.52 (1.06)	0.57 (1.14)
PHQ–2 (pain time point) (mean [SD])	0.54 (1.05)	0.59 (1.12)	0.68 (1.21)
PHQ–2 (health and well-being time point) (mean [SD])	0.46 (1.01)	0.48 (1.06)	0.56 (1.19)
PHQ–2 (mental well-being time point) (mean [SD])	0.52 (1.07)	0.56 (1.12)	0.64 (1.22)
Age (initial time point) (mean [SD])	54.31 (8.13)	57.30 (7.94)	58.53 (7.60)
Age (repeat time point) (mean [SD])	60.05 (7.38)	61.87 (7.11)	62.96 (6.61)
Age (imaging time point) (mean [SD])	63.34 (7.82)	65.78 (7.69)	66.00 (7.61)
Age (repeat Imaging time point) (mean [SD])	63.84 (7.40)	66.71 (7.37)	65.32 (6.76)
Age (mental health time point) (mean [SD])	62.25 (7.84)	64.77 (7.63)	65.67 (7.33)
Age (pain time point) (mean [SD])	64.96 (7.75)	67.66 (7.57)	68.35 (7.31)
Age (health and well-being time point) (mean [SD])	67.78 (7.64)	70.26 (7.47)	70.93 (7.28)
Age (mental well-being time point) (mean [SD])	67.98 (7.54)	70.40 (7.42)	71.07 (7.22)

**Table 3.** Estimated differences in depression scores between IL-6 tertile top and bottom third trajectories at ages 10, 13, 16, 19, 22, 25, and 28 years, in ALSPAC

Age (years)	Difference (raw score)	Difference (*Z*-score)	95% CI	*P* (uncorrected)	*P* (FDR)
IL6 tertile: Top vs. Bottom – age 10	−0.273	−0.014	−0.739 to 0.194	0.2522	1
IL6 tertile: Top vs. Bottom – age 13	0.41	0.035	0.126–0.694	0.0047	0.0327
IL6 tertile: Top vs. Bottom – age 16	0.573	0.045	0.258–0.888	0.0004	0.0025
IL6 tertile: Top vs. Bottom – age 19	0.516	0.032	0.125–0.906	0.0097	0.0679
IL6 tertile: Top vs. Bottom – age 22	0.408	0.024	−0.002 to 0.818	0.0511	0.3577
IL6 tertile: Top vs. Bottom – age 25	0.292	0.016	−0.163 to 0.746	0.2086	1
IL6 tertile: Top vs. Bottom – age 28	0.079	0.004	−0.472 to 0.63	0.7795	1

*Note*: Results from the fully adjusted model.

### Measures of depressive symptoms

#### ALSPAC cohort

The Short Mood and Feelings Questionnaire (SMFQ) was used to assess self-reported depressive symptoms at 11 time points between the ages of 10 and 28 years (Supplementary Figure 1, Supplementary Table 3). The SMFQ was administered via mail/e-mail or in clinics. There were four clinic time points (ages 10, 12, 14, and 18 years) and seven remote self-reported (mail) time points (ages 17, 19, 22, 23, 24, 26, and 28 years). The SMFQ is a 13-item questionnaire that measures the presence of depressive symptoms within the last 2 weeks [44]. The SMFQ has been used in clinical populations to assess depressive symptoms [45] and has been shown to predict clinical depression in ALSPAC [44]. Supplementary Table 4 lists the SMFQ items. Each item response is scored from 0 to 2 (0 = “not true,” 1 = “sometimes,” 2 = “true”), where the total summed score ranges from 0 to 26 and where a higher score corresponds to worse depressive symptoms. The mean number of time points per participant was 6.12 (median = 6, mode = 3).

#### UK Biobank cohort

Depressive symptoms were assessed at eight time points (four in-person and four online follow-up questionnaire assessments) using questions from the Patient Health Questionnaire-2 (PHQ-2) which reflect depressed mood and anhedonia (Supplementary Table 5) [46]. PHQ-2 has previously been shown to be a valid screening tool for detecting depression [47–49]. The mean, standard deviation, min, max, and interquartile range of ages at each time point are described in Supplementary Table 6 and Supplementary Figure 2. The mean number of time points per participant was 2.56 (median = 2, mode = 1).

### Measures of blood serum IL-6

#### ALSPAC cohort

Blood samples were collected at the age 9 years (mean age: 9.86 years; SD: 0.31) and high sensitivity serum CRP and IL-6 were measured in 5,059 participants. Details of laboratory methods are described in detail previously [10]. Individuals with serum CRP ≥ 10 mg/L (*N* = 60) were excluded from the main analysis to minimize confounding by chronic inflammatory condition or acute infection [50], consistent with previous studies [10, 38]. The final sample used for analysis consisted of 4,999 participants.

#### 1.1.1. UK Biobank cohort

Proteomic data were extracted by Olink by analyzing blood samples collected at the initial assessment from a subset of UK Biobank participants (*N* = 54,239) (https://biobank.ctsu.ox.ac.uk/crystal/ukb/docs/Olink_proteomics_data.pdf) [51]. This subset of participants consisted of 46,595 randomly selected participants from the initial assessment visit, 6,376 participants selected for the UKB-PPP study, and 1,268 participants who participated in a COVID-19 repeat-imaging study at multiple visits [51]. Then, 2,923 unique proteins were measured using the Olink Explore 3072 Proximity Extension Assay. This including IL-6 protein which was measured in 44,076 participants. Further details on Olink proteomics data are described by UK Biobank here: https://biobank.ndph.ox.ac.uk/showcase/ukb/docs/Olink_proteomics_data.pdf, https://biobank.ndph.ox.ac.uk/showcase/ukb/docs/Olink_1536_B0_to_B7_Analysis_Report.pdf, https://biobank.ndph.ox.ac.uk/showcase/ukb/docs/Olink_1536_B0_to_B7_Normalization.pdf, https://biobank.ndph.ox.ac.uk/showcase/ukb/docs/Olink_1536_B0_to_B7_FAQ.pdf, https://biobank.ndph.ox.ac.uk/showcase/ukb/docs/PPP_Phase_1_QC_dataset_companion_doc.pdf. Individuals with CRP ≥ 10 mg/L (*N* = 1,758) were excluded from the main analysis, to minimize confounding by acute infection and keep analysis consistent to ALSPAC analysis. Details of blood sampling processing for CRP are described by UK Biobank here: https://biobank.ndph.ox.ac.uk/ukb/ukb/docs/haematology.pdf, https://biobank.ndph.ox.ac.uk/showcase/showcase/docs/serum_biochemistry.pdf, https://www.ukbiobank.ac.uk/media/oiudpjqa/bcm023_ukb_biomarker_panel_website_v1-0-aug-2015-edit-2018.pdf. The final sample used for analysis consisted of 40,069 participants (mean age for baseline IL-6 measurement: 56.6 years; SD: 8.10).

### Statistical analysis

#### Deriving trajectories of depressive symptoms

Multilevel growth curve modeling was conducted in R, using the “lme4” package, to create population-averaged trajectories of depression [52]. Briefly, multilevel growth curve modeling clusters repeated measures within individuals. Unlike traditional linear regression, which treats each observation as independent, multilevel growth curve modeling recognizes that repeated measures within the same individual are likely to be correlated, which reduces bias. Furthermore, multilevel growth curve modeling enables the exploration of individual trajectories of change over time. By allowing for random effects at both the individual and group levels, this approach can capture not only mean population trends across the entire sample but also variations in trajectories among different individuals or groups.

Age was centered to 10 years in ALSPAC and 39 years in UK Biobank (the minimum age of all assessments in each cohort) in order to improve model convergence and better interpretation of the results. Continuous covariate variables were Z-score scaled.

We assessed both linear and nonlinear (quadratic, cubic, and quartic) models. The fit of the model was assessed using Bayesian information criterion and likelihood ratio test. A quartic model fitted the ALSPAC data best and a quadratic model fitted the UK Biobank data best (Supplementary Tables 7 and 8).

The models included repeated measures per participant of SMFQ scores for ALSPAC and PHQ-2 scores for UK Biobank and age at which the depression questionnaire was completed. In ALSPAC, the intercept and four polynomial age terms were able to vary across individuals to capture each individual’s unique trajectory (i.e., random intercept and random slopes model). In UK Biobank, the intercept and only linear age terms were able to vary across individuals (i.e., random intercept and random linear slope model). The model did not converge when also including a random quadratic slope term or when trying a cubic model. Both ALSPAC and UK Biobank models included unstructured covariance terms for the random effects.

To examine how IL-6 associated with changes in depressive symptoms, we split participants into IL-6 tertile groups [10, 38]. The models included fixed effects of IL-6 tertile and an interaction of IL-6 tertiles with each of the fixed-effect age polynomial terms. The rationale for this is that categorical groupings of low, medium, and high inflammation are more intuitive to interpret in trajectory models compared to a continuous variable and is easier to visualize. The IL-6 values of the tertile cutoffs for UK Biobank were as follows: minimum bottom tertile = −2.34, between bottom and middle tertiles = −0.310, between middle and top tertiles = 0.304, and maximum top tertile = 10.6. UK Biobank IL-6 data are provided after they apply an in-house normalization method, which involves a log2 transformation (https://biobank.ndph.ox.ac.uk/showcase/ukb/docs/Olink_1536_B0_to_B7_Normalization.pdf). The IL-6 values (mg/ml) of the tertile cutoffs for ALSPAC were as follows: minimum bottom tertile = 0.007, between bottom and middle tertiles = 0.588, between middle and top tertiles = 1.12, and maximum top tertile = 20.1.

#### Calculating mean depressive symptom scores

To assess the association between IL-6 tertile groups and the development of symptoms over time, we created a population trajectory for each IL-6 tertile group. We then calculated the mean depressive symptoms scores at ages 10, 13, 16, 19, 22, 25, and 28 years in ALSAPC and 40, 50, 60, 70, and 80 years in UK Biobank for each of these trajectories, in the fully adjusted models. These age groups were chosen to reduce the number of multiple tests performed while still capturing potentially important developmental changes over time. We then calculated the differences in mean depressive symptoms scores at each of these ages of the IL-6 tertile groups in a pair-wise manner. Further information on how these scores and their differences were calculated for the trajectories is presented elsewhere [29]. Briefly, the depressive symptom scores were calculated for each IL-6 tertile group trajectory. The delta method (which incorporates the estimate, standard errors, and confidence intervals) was then used to compare these two scores (i.e., upper vs. lower tertile, lower vs. middle tertile, upper vs. middle tertile in turn), revealing a mean difference in scores that are derived estimates from each trajectory. Differences in scores were transformed to Z-scores to compare results between ALSPAC and UK Biobank (detailed in Supplementary Methods). *P*-values were adjusted for multiple testing using the false discovery rate (FDR). The number of multiple tests was the number of different time points used to calculate scores for (ALSPAC: *n* tests = 7; UK Biobank: *n* tests = 5).

#### Confounders

Confounders used in the ALSPAC models were the same as described in Edmondson-Stait et al. (2022). Three main models were used: the first was an unadjusted model with no covariates added, the second was adjusted for sex only, and the third fully adjusted model further included covarying for log-transformed BMI (at age 9 years) and maternal education as a marker of socioeconomic status [53, 54]. Maternal education was coded as a binary variable as either “CSE/O-level/Vocational education” or “A-level/degree level of education.” Sex was coded as a binary variable as either “Male” or “Female.” BMI (age 9 years) was calculated by dividing the weight (kg) by the squared height (meters). The distributions and participant counts of these variables are shown in Supplementary Figure 3.

Confounders used in the UK Biobank models were similar to those used in the ALSPAC cohort. Three main models were used: the first was a minimally adjusted model with covariates for protein batch and assessment center at the initial assessment. These two covariates were not available in the ALSPAC cohort due to there being only one assessment center (unlike UK Biobank that had multiple assessment centers) and a protein assay (ELISA) that did not include a batch variable in ALSPAC [10]. The second model was additionally adjusted for sex. The third fully adjusted model included further covarying for log-transformed BMI (at the time of blood sample collection), smoking status, and the Townsend deprivation index as a marker of socioeconomic status as these have been previously shown to associate with inflammation or psychiatric disorders [55, 56]. The distributions and participant counts of these variables are shown in Supplementary Figure 4.

#### Missing data

Missing outcome data in the trajectories analysis were addressed using full information maximum likelihood estimation (FIML), as part of the “lmer” function from the “lme4” package in R [52, 57]. Briefly, this assumes that the probability of an individual missing a measure of depressive symptoms does not depend on their underlying depressive symptoms score at that occasion, given their observed depressive symptoms trajectory at other occasions. We included individuals into the analysis if they had at least one measurement of depression symptoms in order to maximize power [58].

#### Sensitivity analyses

Sensitivity analyses involved investigating the impact of sex, tertile categorization of IL-6, the impact of anti-inflammatory medication and impact of attrition. Previous studies have shown trajectories of depression are different for males and females [29, 30]. Therefore, we created a new variable that split the IL-6 tertiles by sex: female and bottom third IL-6 tertile, female and middle third IL-6 tertile, female and top third IL-6 tertile, male and bottom third IL-6 tertile, male and middle third IL-6 tertile, and male and top third IL-6 tertile. The models were then run splitting the trajectories on this sex-split IL-6 tertile variable and analyzed the same way as in the main analysis. To assess the effect of tertile categorization of IL-6, we ran the analysis using a continuous measure of IL-6 (which was inverse normal transformed to achieve normal distribution and Z-score scaled) and compared the model estimates. The impact of inflammatory medication was assessed by removing individuals who might be taking medication that affects inflammation (ALSPAC N removed = 695; UK Biobank N removed = 10,652). In ALSPAC, the only measure available for medication at age 9 years (when IL-6 was measured) was a general variable of “Currently taking medication?,” therefore this may include medications that do not impact inflammation. In the UK Biobank, anyone taking anti-inflammatory medications were removed (Supplementary Material; Supplementary Table 9). To assess attrition, we used linear regression to test for associations between IL-6 tertile on the number of questionnaires completed. In UK Biobank, the two imaging time points were excluded in this count as only a subset of individuals were invited to attend these appointments. To further assess attrition, we also ran the trajectory models limiting individuals to only those that had attended at least two assessments.

Additionally, we assessed the differences in markers of socioeconomic status used in ALSPAC and UK Biobank. To ensure consistency with our previous ALSPAC study we used maternal education as a marker of socioeconomic status [11]. However, Townsend deprivation index was used as a marker of socioeconomic status in UK Biobank as no measure of maternal education was available. Therefore, we also conducted a sensitivity analysis in ALSPAC using Townsend deprivation index quintiles as a covariate in place of maternal education allowing a comparison of results between ALSPAC and UK Biobank (Supplementary Material, Supplementary Figure 5).

In UK Biobank various other sensitivity analyses were performed. Other factors that may affect inflammation included inflammatory conditions and high BMI. Individuals with an inflammatory condition were identified and removed (*N* removed = 6,342), using definitions of 49 conditions [59] (Supplementary Table 10). Individuals with BMI ≥ 40 were also removed (*N* removed = 577), as inflammation associates with high BMI (Supplementary Material) [3]. Finally, to assess attrition due to death we removed people who had died after the initial baseline appointment. Further details are in the Supplementary Material. Similar analysis was not conducted in ALSPAC due to this cohort being a younger age.

## Results

### Sample characteristics of ALSPAC

A total of 4,999 individuals had serum IL-6 data and CRP < 10 mg/L, of these 4,835 had at least one measurement of depressive symptoms (measured by the SMFQ). Sample characteristics of this sample are shown in Table 1, split by IL-6 tertile.

**Table 4.** Estimated differences in depression scores between IL-6 tertile top and bottom third trajectories at ages 10, 13, 16, 19, 22, 25, and 28 years, in ALSPAC, split by sex

Age (years)	Difference (raw score)	Difference (*Z*-score)	95% CI	*P* (uncorrected)	*P* (FDR)
IL6 tertile: Female_Top vs. Female_Bottom – age 10	0.226	0.009	−0.411 to 0.864	0.4864	1
IL6 tertile: Female_Top vs. Female_Bottom – age 13	0.597	0.037	0.202–0.992	0.003	0.0213
IL6 tertile: Female_Top vs. Female_Bottom – age 16	0.683	0.039	0.251–1.114	0.0019	0.0135
IL6 tertile: Female_Top vs. Female_Bottom – age 19	0.605	0.029	0.086–1.123	0.0224	0.1565
IL6 tertile: Female_Top vs. Female_Bottom – age 22	0.451	0.02	−0.089 to 0.99	0.1015	0.7106
IL6 tertile: Female_Top vs. Female_Bottom – age 25	0.275	0.011	−0.321 to 0.871	0.3652	1
IL6 tertile: Female_Top vs. Female_Bottom – age 28	0.099	0.003	−0.608 to 0.806	0.7836	1
IL6 tertile: Male_Top vs. Male_Bottom – age 10	−0.112	−0.004	−0.754 to 0.529	0.7313	1
IL6 tertile: Male_Top vs. Male_Bottom – age 13	0.159	0.01	−0.237 to 0.555	0.4319	1
IL6 tertile: Male_Top vs. Male_Bottom – age 16	0.141	0.008	−0.317 to 0.6	0.5455	1
IL6 tertile: Male_Top vs. Male_Bottom – age 19	0.157	0.007	−0.429 to 0.743	0.5988	1
IL6 tertile: Male_Top vs. Male_Bottom – age 22	0.276	0.011	−0.347 to 0.9	0.3845	1
IL6 tertile: Male_Top vs. Male_Bottom – age 25	0.316	0.011	−0.397 to 1.029	0.385	1
IL6 tertile: Male_Top vs. Male_Bottom – age 28	−0.159	−0.004	−1.038 to 0.72	0.7226	1

*Note*: Results from the fully adjusted model.

### Sample characteristics of UK Biobank

A total of 40,069 individuals had IL-6 data and CRP < 10 mg/L, of these 39,613 had at least one measurement of depressive symptoms (measured by PHQ-2; 18,958 had depressive symptoms measured only at the initial assessment). Sample characteristics of this sample are shown in Table 2, split by IL-6 tertile. IL-6 was measured in the initial assessment in which participant ages ranged from age 39 to 70 years (mean: 56.6 years; SD: 8.10). The mean ages varied for participants in each IL-6 tertile (bottom third: mean: 54.31 years, SD: 8.13; middle third: mean: 57.30 years, SD: 7.94; top third: mean: 58.53 years, SD: 7.60).

### Associations of baseline IL-6 with subsequent depressive symptoms trajectories in ALSPAC

**Figure 1.**
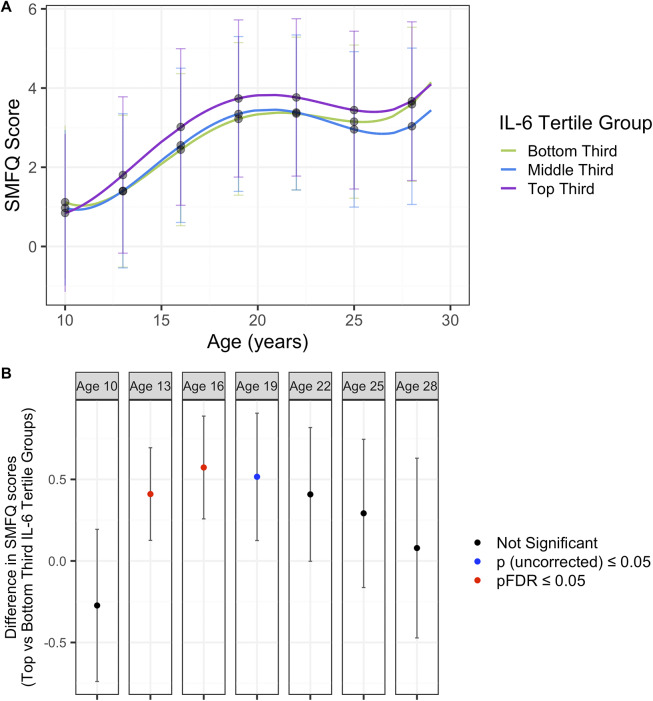
(A) Depression trajectories in ALSPAC split by IL-6 tertile groups. (B) Differences in depression scores in ALSPAC between the top and bottom third IL-6 tertiles. Results from the fully adjusted model. Mean depressive scores were calculated from the depression trajectories in each IL-6 tertile at ages 10, 13, 16, 19, 22, 25, and 28 years. Differences between the top and bottom third IL-6 tertile trajectories were calculated using the delta method. *P*-values are corrected for multiple corrections (FDR).

In the fully adjusted model (sex, BMI and maternal education), the overall pattern of depressive symptom trajectories increased from ages 10 to 20 years, followed by a plateau from 22 years onward. The top third IL-6 tertile group had a higher trajectory compared to both the middle and bottom third IL-6 tertile groups, indicating increased depressive symptoms across this period (Figure 1A, Supplementary Table 11). However, confidence intervals overlapped across all IL-6 tertile group trajectories. Model estimates for all models (unadjusted, sex adjusted and fully adjusted) are presented in Supplementary Table 12. In the fully adjusted model, the intercept score at 10 years of age for the baseline group (bottom third IL-6 tertile) was 1.1192 (SE = 0.9868), the linear rate of change was <0.001 (SE = 0.12), the quadratic rate of change was 0.1355 (SE = 0.0274), the cubic rate of change was <0.0001 (SE = 0.0022) and the quartic rate of change was 0.0003 (SE < 0.0001). The interaction between the top third IL-6 tertile and linear age strongly associated with depressive symptoms at the age at the intercept (10 years) (β = 0.3581, *p* = 0.037; Supplementary Table 12). All other IL-6 tertile terms and their interactions with age did not associate with depressive symptoms at the age at the intercept (10 years) (Supplementary Table 12).

Given the difficulty in interpreting nonlinear trajectories (i.e., positive linear polynomial terms, and negative quadratic polynomial terms), we report the mean differences at various ages across youth development between the bottom third and top third IL-6 tertile groups. There was evidence for a difference in depressive symptom scores between the top and bottom third IL-6 tertiles across adolescence at ages 13 (SMFQ Score^diff^ = 0.41, 95% CI 0.126–0.694, pFDR = 0.0327) and 16 years (SMFQ Score^diff^ = 0.573, 95% CI 0.258–0.888, pFDR = 0.0025), but not the other ages tested (10, 19, 22, 25 and 28 years) (Figure 1B,Table 3).

**Figure 2.**
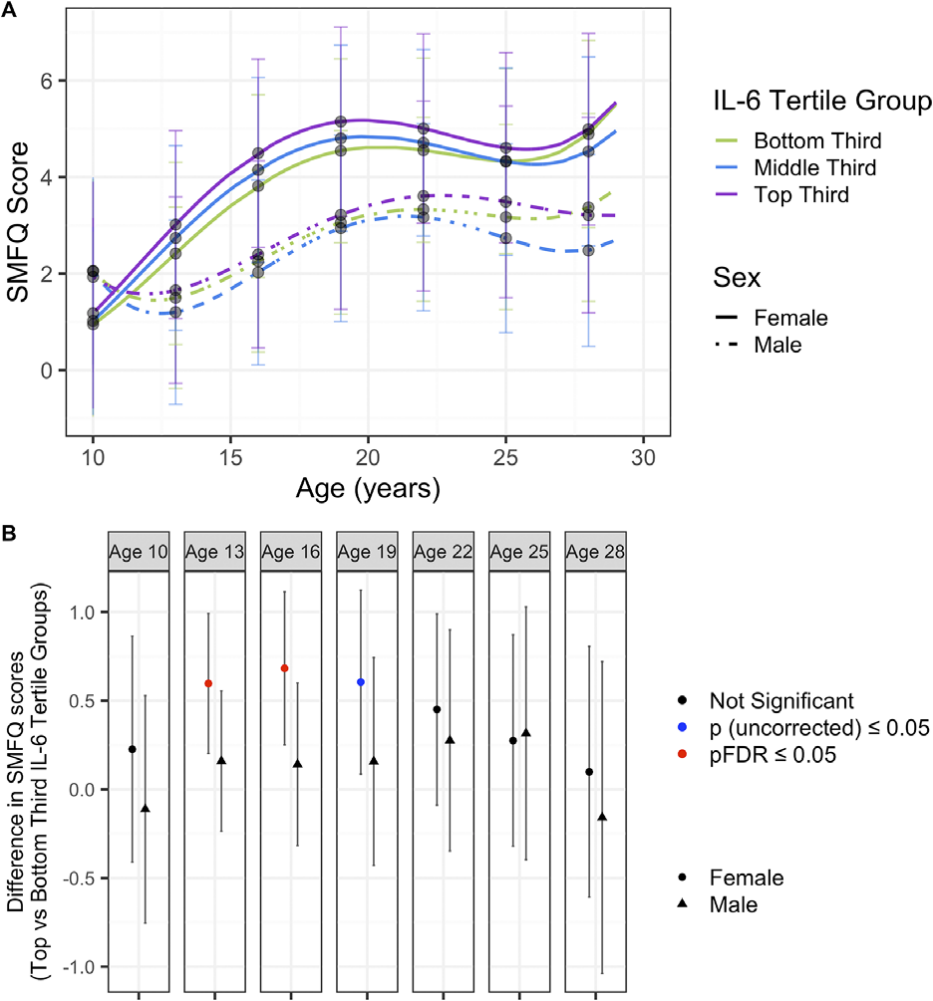
(A) Depression trajectories in ALSPAC split by sex and IL-6 tertile groups. (B) Differences in depression scores in ALSPAC between the top and bottom third IL-6 tertiles, in males and females, separately. Results from the fully adjusted model. Mean depressive scores were calculated from the depression trajectories in each IL-6 tertile split by sex at ages 10, 13, 16, 19, 22, 25, and 28 years. Differences between the top and bottom third IL-6 tertile trajectories were calculated using the delta method. *P*-values are corrected for multiple corrections (FDR).

There was evidence for differences in trajectories between females and males when splitting each IL-6 tertile group by sex. Depression trajectories and mean depressive scores across all IL-6 tertiles were worse in females compared to males (Figure 2A, Table 4, Supplementary Table 13). Females in the top third IL-6 tertile group generally had worse trajectories. There was evidence for a difference in depressive symptom scores between the top and bottom third IL-6 tertiles in females, but not males, at ages 13 and 16 years (Figure 2B, Table 4, Supplementary Table 14).

Similar results were also observed when using a continuous measure of IL-6, when individuals taking any medication were removed, and when using Townsend deprivation index quintiles in place of maternal education (Supplementary Tables 15–17). These results were unlikely to be bias by attrition, as IL-6 tertiles did not associate with the number of completed questionnaires (Supplementary Table 18, Supplementary Figure 6). Additionally, similar results were seen in the trajectory models when limiting the sample to individuals who had attended at least two assessments (Supplementary Table 19; Supplementary Figure 7).

### Associations of baseline IL-6 with subsequent depressive symptoms trajectories in UK Biobank

**Figure 3.**
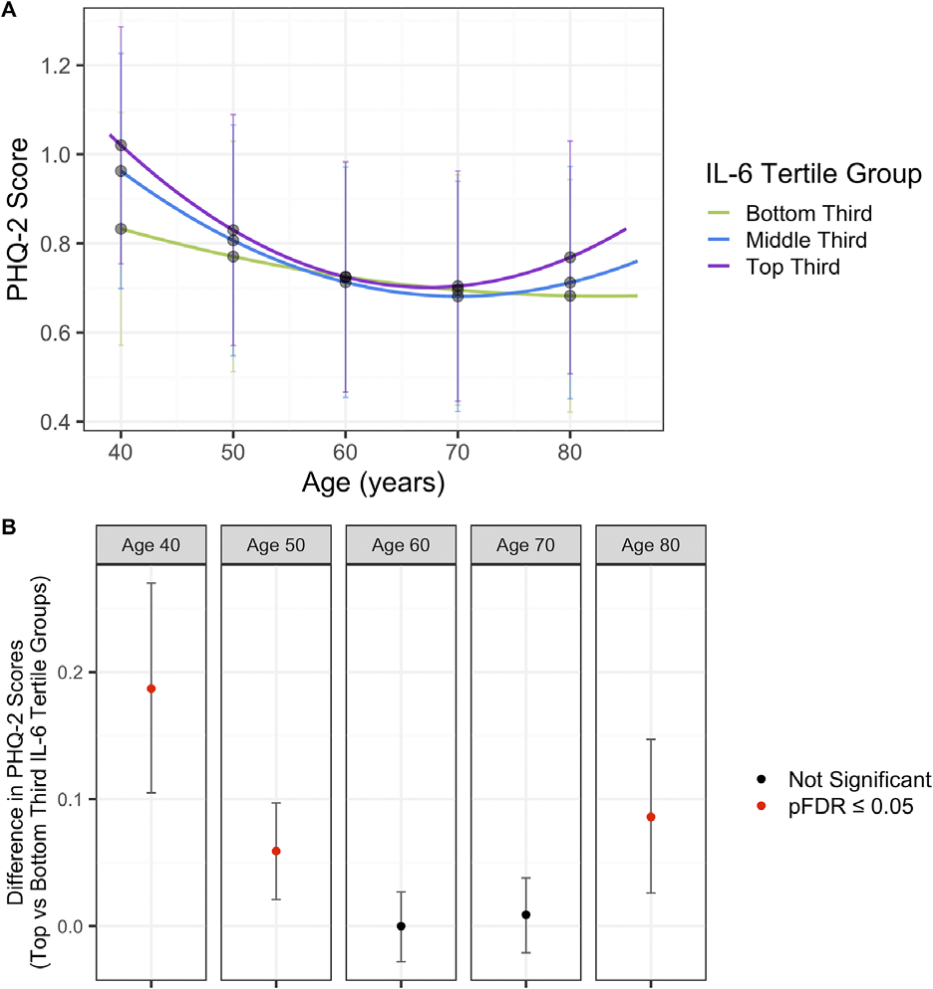
(A) Depression trajectories in UK Biobank split by IL-6 tertile groups. (B) Differences in depression scores in UK Biobank between the top and bottom third IL-6 tertiles. Results from the fully adjusted model. Mean depressive scores were calculated from the depression trajectories in each IL-6 tertile at ages 40, 50, 60, 70, and 80 years. Differences between the top and bottom third IL-6 tertile trajectories were calculated using the delta method. *P*-values are corrected for multiple corrections (FDR).

In the fully adjusted model (sex, BMI, batch, assessment center, Townsend deprivation index and smoking status), the overall pattern of depressive symptom trajectories decreased from age 39 years until mid-60 years where they begin to increase. The top third IL-6 tertile group had a higher trajectory compared to both the middle and bottom third IL-6 tertile groups, indicating increased depressive symptoms across this period (Figure 3A, Supplementary Table 20). However, the confidence intervals overlapped across all IL-6 tertile group trajectories. Model estimates for all models (unadjusted, sex adjusted, and fully adjusted are presented in Supplementary Table 21). In the fully adjusted model, the intercept score at 39 years of age for the baseline group (bottom third IL-6 tertile) was 0.8401 (SE = 0.1338), the linear rate of change was <0.001 (SE = 0.0022), and the quadratic rate of change was <0.0001 (SE < 0.0001). All the IL-6 tertile terms and their interactions with age were strongly associated with depressive symptoms at the intercept age of 39 years. Specifically, the middle and top third IL-6 tertile positively associated with depressive scores at the intercept age of 39 years compared to the lower third IL-6 tertile group (middle third IL-6 tertile: β = 0.1417, *p* = 0.0004; top third IL-6 tertile: β = 0.2041, p < 0.0001). The interactions between the middle and top third IL-6 tertiles and linear age were also associated with depressive symptoms (middle third IL-6 tertile × linear age: β < 0.0001, *p* = 0.0003; top third IL-6 tertile × linear age: β < 0.0001, *p* < 0.0001). Additionally, interactions between the middle and top third IL-6 tertiles and quadratic age were associated with depressive symptoms (middle third IL-6 tertile × quadratic age: β = 0.0002, *p* = 0.0015; top third IL-6 tertile × quadratic age: β = 0.0003, *p* < 0.0001).

There was evidence for a difference in depressive symptom scores between the top and bottom third IL-6 tertile groups at ages 40 (PHQ-2 Score^diff^ = 0.187, 95% CI 0.105–0.27, pFDR<0.0001), 50 (PHQ-2 Score^diff^ = 0.059, 95% CI 0.021–0.097, pFDR = 0.0111), and 80 years (PHQ-2 Score^diff^ = 0.086, 95% CI 0.026–0.147, pFDR = 0.0257), but not the other ages tested (Figure 3B, Table 5).

**Table 5.** Estimated differences in depression scores between different IL-6 tertile trajectories at ages 40, 50, 60, 70, and 80 years, in UK Biobank

Age (years)	Difference (raw score)	Difference (*Z*-score)	95% CI	*P* (uncorrected)	*P* (FDR)
IL6 tertile: Top vs. Bottom – age 40	0.187	0.018	0.105–0.27	*p* < 0.0001	*p* < 0.0001
IL6 tertile: Top vs. Bottom – age 50	0.059	0.013	0.021–0.097	0.0022	0.0111
IL6 tertile: Top vs. Bottom – age 60	0	0	−0.028 to 0.027	0.9728	1
IL6 tertile: Top vs. Bottom – age 70	0.009	0.002	−0.021 to 0.038	0.5692	1
IL6 tertile: Top vs. Bottom – age 80	0.086	0.011	0.026–0.147	0.0051	0.0257

*Note*: Results from the fully adjusted model.

**Figure 4.**
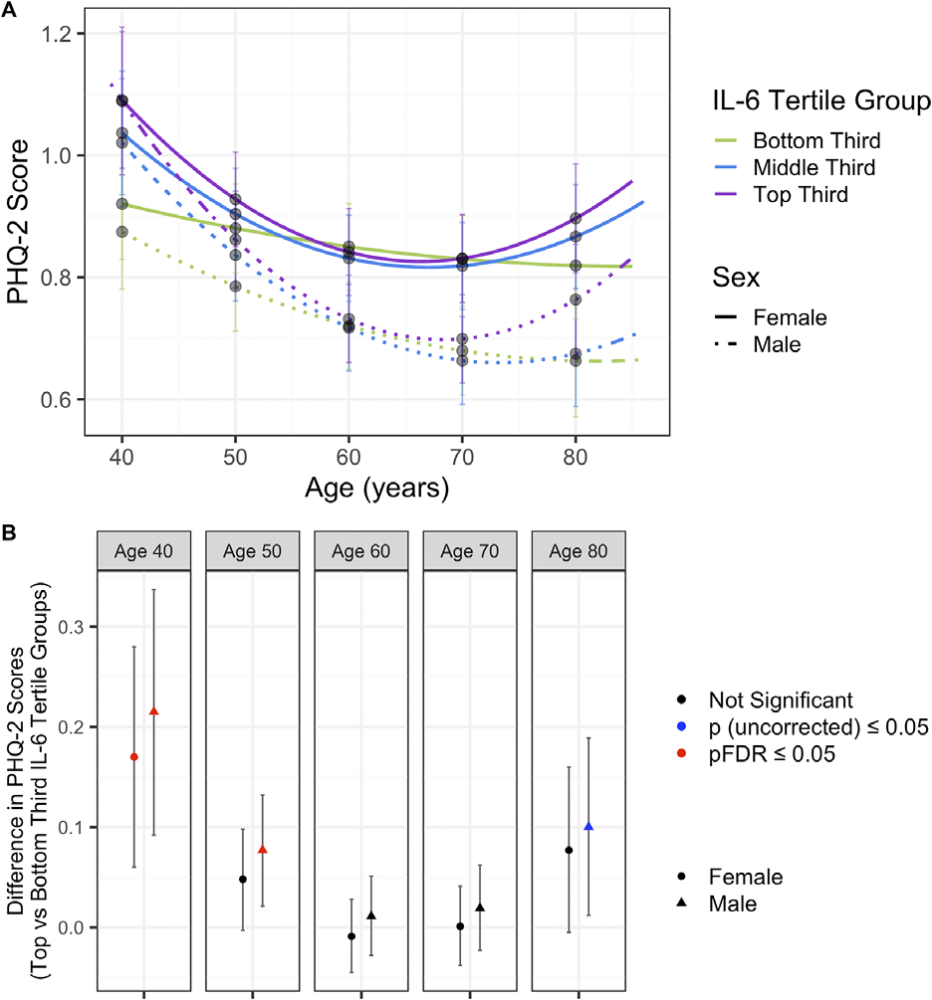
(A) Depression trajectories in UK Biobank split by sex and IL-6 tertile groups. (B) Differences in depression scores in UK Biobank between the top and bottom third IL-6 tertiles, in males and females separately. Results from the fully adjusted model. Mean depressive scores were calculated from the depression trajectories in each IL-6 tertile split by sex at ages 40, 50, 60, 70, and 80 years. Differences between the top and bottom third IL-6 tertile trajectories were calculated using the delta method. *P*-values are corrected for multiple corrections (FDR).

There was evidence for differences between females and males when splitting each IL-6 tertile group by sex. Depression trajectories and mean depressive scores across all IL-6 tertiles were generally worse in females compared to males (Figure 4A, Supplementary Table 22). There was evidence for a difference in depressive symptom scores between the top and bottom third IL-6 tertiles in both males and females at age 40 years, in males only at age 50 years, but not for ages 60, 70, or 80 years (Figure 4B, Table 6, Supplementary Table 23).

**Table 6.** Estimated differences in depression scores between different IL-6 tertile trajectories at ages 40, 50, 60, 70, and 80 years, in UK Biobank, split by sex

Age (years)	Difference (raw score)	Difference (*Z*-score)	95% CI	*P* (uncorrected)	*P* (FDR)
IL6 tertile: Female_Top vs. Female_Bottom – age 40	0.17	0.012	0.06–0.28	0.0025	0.0124
IL6 tertile: Female_Top vs. Female_Bottom – age 50	0.048	0.008	−0.003 to 0.098	0.0651	0.3257
IL6 tertile: Female_Top vs. Female_Bottom – age 60	−0.009	−0.002	−0.045 to 0.028	0.6358	1
IL6 tertile: Female_Top vs. Female_Bottom – age 70	0.001	0	−0.038 to 0.041	0.9536	1
IL6 tertile: Female_Top vs. Female_Bottom – age 80	0.077	0.008	−0.005 to 0.16	0.0651	0.3253
IL6 tertile: Male_Top vs. Male_Bottom – age 40	0.215	0.014	0.092–0.337	0.0006	0.0029
IL6 tertile: Male_Top vs. Male_Bottom – age 50	0.077	0.011	0.021–0.132	0.0067	0.0335
IL6 tertile: Male_Top vs. Male_Bottom – age 60	0.011	0.002	−0.028 to 0.051	0.5675	1
IL6 tertile: Male_Top vs. Male_Bottom – age 70	0.019	0.004	−0.023 to 0.062	0.3687	1
IL6 tertile: Male_Top vs. Male_Bottom – age 80	0.1	0.009	0.012–0.189	0.0256	0.128

*Note*: Results from the fully adjusted model.

Similar results were also observed when using a continuous measure of IL-6, when individuals taking anti-inflammatory medication were removed, when individuals with inflammatory conditions were removed, when individuals with BMI ≤ 40 were removed, and when subsetting to only participants who were alive after the initial appointment (Supplementary Tables 24–28). However, these results may have been biased by attrition, as IL-6 tertiles were associated with the number of completed questionnaires in the sample of participants that remained alive after the initial assessment (Supplementary Table 29, Supplementary Figures 8 and 9). Additionally, trajectory models with samples limited to individuals who had attended at least two assessments showed smaller effect sizes than in the main analysis (Supplementary Table 30; Supplementary Figure 10).

## Discussion

Longitudinal trajectories of depressive symptoms were modeled to investigate the effects of baseline IL-6 on depressive symptoms in two cohorts spanning different stages of the life course (ALSPAC and UK Biobank). Higher IL-6 was associated with worse trajectories of depression symptoms across the life course. This relationship was stronger in the younger cohort (ALSPAC), compared to the older cohort (UK Biobank). Sex differences were also consistent in both cohorts but stronger in the younger cohort (ALSPAC), where the association between higher IL-6 and worse depression trajectories was stronger in females compared to males.

The main strengths of this study are the use of two large-scale population cohorts with prospectively collected data and repeated measures of depression symptoms at 11 assessments across ages 9–28 years in ALSPAC and 8 assessments across ages 39–80 years in UK Biobank. This permitted the investigation of identifying the ages where increased IL-6 associated with worse depression trajectories and whether these effects were persistent across different stages of the life course. Similar relationships were observed between IL-6 and depression trajectories in two different cohorts despite their heterogeneity and no overlap in ages. Also presented is an alternative way of interpreting trajectory results by looking at mean differences in scores at ages, which has only recently been developed in the field of longitudinal epidemiology [29, 60].

The overall pattern of depression trajectories in ALSPAC was consistent with previous studies of the same cohort [29, 61]. Depressive symptoms increased from ages 10 to 20 years, followed by a plateau from 22 years onward. Depression trajectories have been modeled in other cohorts and show a similar pattern to the UK Biobank results in this current study [31], whereby symptoms decrease from age 40 years until mid-60 years where they begin to increase again. There was evidence that people with higher IL-6 (i.e., in the top third IL-6 tertile group) had worse trajectories than those with lower IL-6 (i.e., in bottom third and middle third IL-6 tertile groups), with the greatest difference in mean depressive symptom scores between the top and bottom third IL-6 tertile groups observed at ages 13 and 16 years in ALSPAC and 40, 50, and 80 years in UK Biobank. The Z-scores of the mean differences for these ages were also comparable between ALSPAC and UK Biobank, with slightly larger mean differences in ALSPAC (0.039–0.046) compared to UK Biobank (0.011–0.018). This suggests that at various points across the life course, higher IL-6 associates with worse depression symptom trajectories, with a relatively greater impact of IL-6 on depressive symptoms in younger compared to older people. Inflammation has been shown to associate with changes in brain structure, which could be one mechanism by which inflammation may contribute to depression, especially during this vulnerable period of neurodevelopment [7, 8, 62]. An MR study investigating the effect of peripheral inflammatory markers on brain structure in older adults from UK Biobank found potential causal mechanisms for serum IL-6 on regions associated with major psychiatric disorders (temporal, fusiform and frontal cortices) [9]. Chronic, systemic inflammation is also associated with increased age, termed “inflammaging,” which has been linked to various age-related illnesses [63]. Brain related inflammaging such as increased neuroinflammation and reduced blood–brain barrier integrity, which are proposed mechanisms for depression in later life [63]. Additionally, it may be that other environmental and social factors having more prominent effects on depression at later stages of the life course [64]. It should also be noted that there is a difference in the IL-6 assay method used in ALSPAC and UK Biobank. ALSPAC used ELISA, which is commonly used for assessing IL-6 measures, whereas UK Biobank used Olink, which is a high-throughput method and may not be as accurate as ELISA. Additionally, these measures are on different scales. ALSPAC IL-6 data are provided as raw pg/ml measurements, whereas UK Biobank is provided after they apply an in-house normalization method that involves a log2 transformation.

The findings in this study are in line with previous studies. Previous studies have shown that higher IL-6 is associated with depressive symptoms in both a cross-sectional and cross-lag relationships [3, 10, 16, 24, 38, 65]. Our previous study found that IL-6 was associated with the total number of depressive episodes, representing increased burden of depression in ALSPAC [11]. Cross-sectionally, an inflammatory subgroup of depression has been shown to associate with depression severity [13]. Another study using latent class analysis in ALSPAC showed that baseline serum IL-6 levels were associated with a trajectory group of persistently worse depressive symptoms from ages 10 to 19 years [24]. This current study complemented and extended these studies in numerous ways. First, by extending the age range investigated in ALSPAC to also include a period of early adulthood (up to 28 years) in which the development of psychiatric disorders can occur. Second, analysis was conducted in cohorts of both younger (ALSPAC) and older (UK Biobank) age with prospectively collected data. Third, population-level trajectories of depressive symptoms were assessed using multilevel growth models, rather than probabilistic membership into groups of individuals identified from latent class analysis. Briefly, multilevel growth modeling clusters repeated measures within individuals to capture changes over time. It can also model random effects at both the individual and group levels. This allows for the assessment of not only mean population trends across the entire sample but also variations in trajectories among different individuals or groups (e.g. IL-6 tertile subgroups or males and females). Whereas latent class modeling assumes there are homogenous subgroups that follow similar longitudinal trajectories, and estimates these unobserved (latent) subgroups, rather than modeling predefined or observed groups [66].

The choice of using multilevel modeling for this study was decided based on several factors. First, a limitation of latent class modeling in the context of this study is the sensitivity to the number of time points included in the analysis and the comparison between what different latent classes might mean between the ALSPAC and UKB cohorts (i.e., what might increasing and decreasing trajectories mean in the context of the different developmental windows). Studies using the SMFQ depression data in ALSPAC have demonstrated this sensitivity, with different studies identifying varying numbers of latent classes based on the number of time points analyzed [24, 30, 67, 68]. Such fluctuations make it challenging to study the true relationships between risk factors and outcomes, as the latent groups themselves may shift depending on the study design, whereas multilevel modeling is less sensitive to such changes in the number of time points, as it models trajectories on observed groups of individuals.

There was also strong evidence of the sex-specific effects of IL-6 on depression trajectories in ALSPAC and weaker evidence in the UK Biobank. Previous studies have shown that females have worse depression trajectories than males in ALSPAC [29-31]. Here, in addition to showing this, sex differences in depression trajectories were also shown to persist into older adulthood (39–86 years). This is consistent with the findings in other cohorts [31]. In ALSPAC, there was evidence that the difference in depressive scores between the top and bottom third IL-6 tertiles at ages 13 and 16 years was greater in females than in males. Similar findings have been reported elsewhere, showing that IL-6 associates with more severe depression in female but not male adolescents [33]. This could be due to hormonal changes that occur during pubertal development [69]. Female sex hormones, such as estrogen, have also been shown to have effects on the immune system, although depending on the context can have both anti- or pro-inflammatory effects [70]. However, there may also be methodological explanations for this sex difference, such as there are a greater number of females than males in ALSPAC. Whereas in UK Biobank, the differences in scores between the top third and bottom third IL-6 tertiles remained in both males and females for ages 40 years but occurred only in males at age 50 years and diminished at age 80 years. This could be attributed to a variety of explanations, including that in general inflammation increases with age [71]. In UK Biobank, the ages of participants at the initial assessment when IL-6 was measured varied from age 39 to 70 years. Whereas in ALSPAC IL-6 was measured at age 9 years. This led to differences in the mean ages for UK Biobank participants for each IL-6 tertile group, with an older mean age for each tertile as IL-6 increases. However, age was included in the models. Future studies should assess the relationship of inflammatory markers measured at the same age on depression trajectories in older individuals to strengthen the findings in this current study.

However, extensive sensitivity analyses from both cohorts showed that these findings persisted when controlling for factors that typically affect depression and inflammation. In ALSPAC, these findings were robust against adjusting for covariates sex, BMI, and socioeconomic markers (maternal education or Townsend deprivation index quintiles). Sensitivity analyses removing individuals taking any medication also resulted in similar model coefficients. In UK Biobank, these findings were robust against adjusting for covariates sex, BMI, Townsend deprivation index, and smoking status.

The UKB-PPP study was enriched for people with ill health; therefore, the sample used for the UK Biobank analysis may be bias to this [51]. This was accounted for in the sensitivity analyses removing individuals with an inflammatory condition, with BMI ≥ 40 or who were taking anti-inflammatory medication, which showed similar effects to the main analysis. In both ALSPAC and UK Biobank, using a continuous measure of IL-6 showed similar results to using IL-6 tertile groups.

However, it should be noted that these effect sizes are small, and the confidence intervals are wide (despite the large sample sizes). This could be due to some limitations of the study. Both ALSPAC and UK Biobank are population-based cohorts rather than clinical cohorts. Furthermore, UK Biobank participants are more likely to be female and living in less socioeconomically deprived areas than the general population [72]. Additionally, although the age range of participants in UK Biobank is 39–86 years, the mean participants age is between 57 and 70 years for each time point (Supplementary Table 6). This contributes to higher confidence intervals in the distal ages, and therefore, the results should be interpreted with this in consideration. Additionally, in UK Biobank, the repeat imaging assessment had the lowest sample size across all time points assessed (*N* = 331). This may affect the robustness of the growth curve estimation for the age range covered by this assessment. However, there is some overlap with this age range and other assessment time points with larger sample sizes (Supplementary Table 2; Supplementary Table 6). It should also be noted only one inflammatory marker, serum IL-6, was investigated in this study. This is due to previous longitudinal and MR studies finding the strongest associations between this marker and depression outcomes, even when additional inflammatory markers were investigated [9-11, 16]. However, in addition to inflammatory processes, IL-6 has other physiological roles, such as tissue repair and lipolysis in the liver, which can occur in the absence of inflammation [73]. Future studies should investigate multiple markers that form inflammatory pathways to fully understand the role of inflammation in depression. This will require careful consideration in incorporating statistical approaches capturing the highly correlated structure of inflammatory markers with trajectory modeling.

There are also other limitations to consider in this study. Both ALSPAC and UK Biobank suffer from attrition, and in UK Biobank, 48% only had one measurement of depressive symptoms. These individuals were retained in the analysis as they contribute to the relationship between IL-6 and depression, and a key advantage of multilevel models is that it uses FIML to account for missing outcome data. However, if the data are not missing at random then this method would be biased. We conducted sensitivity tests and found that the IL-6 tertile group associated with the number of times a participant completed a questionnaire associated in UK Biobank but not in ALSPAC. In the UK Biobank, this sensitivity analysis was done in participants that remained alive after the initial assessment, due to this cohort being an older sample, and excluded the two imaging appointments. Additionally, trajectory models with samples limited to individuals who had attended at least two assessments showed smaller effect sizes than in the main analysis in UK Biobank but not in ALSPAC. These individuals with at least two assessments also had lower IL-6 at baseline compared to individuals with only one assessment (Supplementary Figure 10). This suggests that there is likely some bias between the IL-6 tertile group and subsequent attrition with data not missing at random in UK Biobank, but not in ALSPAC. This is similar to findings showing healthy participation bias in UK Biobank affects downstream analyses in genetic epidemiology studies [74].

In conclusion, the findings in this study suggest that high IL-6 associates with worse depression symptom trajectories observed at different stages of the life course, with stronger associations in younger individuals. However, these statistically significant associations (pFDR <0.05) have smaller effect sizes, which is typical of large cohort studies. On further analysis of sex differences, this association was stronger in females, compared to males in early adolescence. Whereas weaker sex differences were observed in later life. Future studies could also investigate the trajectories of different depression subtypes, such as atypical depression, and whether inflammatory proteins from a wider panel of markers influence their trajectories across the life course.

## Supporting information

Edmondson-Stait et al. supplementary material Edmondson-Stait et al. supplementary material

## Data Availability

The data used in the present study are available from UK Biobank and ALSPAC with restrictions applied. Data were used under license and thus not publicly available. Access to the UK Biobank data can be requested through a standard protocol (https://www.ukbiobank.ac.uk/register-apply/). The ALSPAC study website contains the details of all data available: http://www.bristol.ac.uk/alspac/researchers/our-data. The code used for the analysis is publicly available on GitHub (www.github.com/ameliaes/2025_EurPsych).

## Supplementary material

The supplementary material for this article can be found at https://doi.org/10.1192/j.eurpsy.2025.7.
