# Supplementary material for: Associations between IL-6 and trajectories of depressive symptoms across the life course: Evidence from ALSPAC and UK Biobank cohorts
Source: Eur Psychiatry. 2025 Jan 27;68(1):e27. doi: 10.1192/j.eurpsy.2025.7 (PMC11883784; doi:10.1192/j.eurpsy.2025.7)
Supplement: Edmondson-Stait et al. supplementary material [file S0924933825000070sup001.docx]

**Supplementary Material for “Associations between IL-6 and trajectories of depressive symptoms across the life course: Evidence from ALSPAC and UK Biobank cohorts”**

Supplementary Methods

ALSPAC Cohort

Study data [1-3] were collected and managed using REDCap electronic data capture tools hosted at the University of Bristol [4]. REDCap (Research Electronic Data Capture) is a secure, web-based software platform designed to support data capture for research studies. Please note that the study website contains details of all the data that is available through a fully searchable data dictionary and variable search tool: http://www.bristol.ac.uk/alspac/researchers/our[-](http://www.bristol.ac.uk/alspac/researchers/our-data/)data/. Ethical approval for the study was obtained from the ALSPAC Ethics and Law Committee and the Local Research Ethics Committees. Consent for biological samples has been collected in accordance with the Human Tissue Act (2004). Informed consent for the use of data collected via questionnaires and clinics was obtained from participants following the recommendations of the ALSPAC Ethics and Law Committee at the time.

Z-score calculation

Z-scores of the differences between depression questionnaire scores were calculated to compare results between ALSPAC and UK Biobank. This was done in R by calculating the difference in scores / ((upper 95% CI – lower 95% CI) / 3.1999 ) * sqrt(sample size).

Sensitivity analysis using Townsend deprivation index quintiles in ALSPAC

The socioeconomic variables used for ALSPAC and UK Biobank were different in the main analysis. In UK Biobank we used Townsend deprivation index as a continuous score of material deprivation calculated from using data on non-car ownership, non-home ownership, unemployment and overcrowding within geographic regions [5]. A sensitivity analysis was performed in ALSPAC where Townsend deprivation index quintiles were included as a covariate in place of maternal education so results across ALSPAC and UK Biobank could be compared.

Townsend deprivation index quintiles were measured at three timepoints in ALSPAC (age 4, 6 and 16 years). The majority of individuals did not change in the quintile they belonged to across these three time points (Supplementary Figure 5). The variable measured at the 6-year time point was used as the covariate in the sensitivity analysis as this was closest to the time point at which blood samples were taken for IL-6 measurements.

Categorised definitions for BMI and BMI ≥ 40 BMI sensitivity analysis

BMI was categorised into the following groups in UK Biobank: BMI < 18.5 = underweight, BMI 18.5 – 24.9 = healthy weight, BMI 25 – 29.9 = overweight, BMI 30-39.9 = Obese and BMI ≥ 40 = morbidly obese [6]. Numbers of individuals in each group are shown in the demographic table (Table 2). Individuals with BMI ≥ 40 were removed in a sensitivity analysis to ensure any inflammation due to high BMI was not affecting the results. Similar analysis was not conducted in ALSPAC as these BMI category definitions are for adults.

Sensitivity analysis of alive UK Biobank participants and number of completed questionnaires covariate

To assess whether the number of questionnaires someone had completed affected the results we first removed people who had died (N removed = 2,815) after the initial baseline appointment and secondly included a covariate for the number of questionnaires someone had completed (excluding the two imaging time points as only a subset of individuals were invited to attend these appointments).

Medication Sensitivity Analysis

In both cohorts we removed individuals that might be taking medication that affects inflammation (ALSPAC N removed = 695; UK Biobank N removed = 10,652). In ALSPAC, the only measure available for medication at age 9 years (when IL-6 was measured) was a general variable of “Currently taking medication?”, therefore this may include medications that do not impact inflammation. In UK Biobank, anyone taking anti-inflammatory medications were removed (Supplementary Table 7).

Supplementary Figures

Supplementary Figure 1. Box plot of ages of individuals at each appointment time point in ALSPAC.


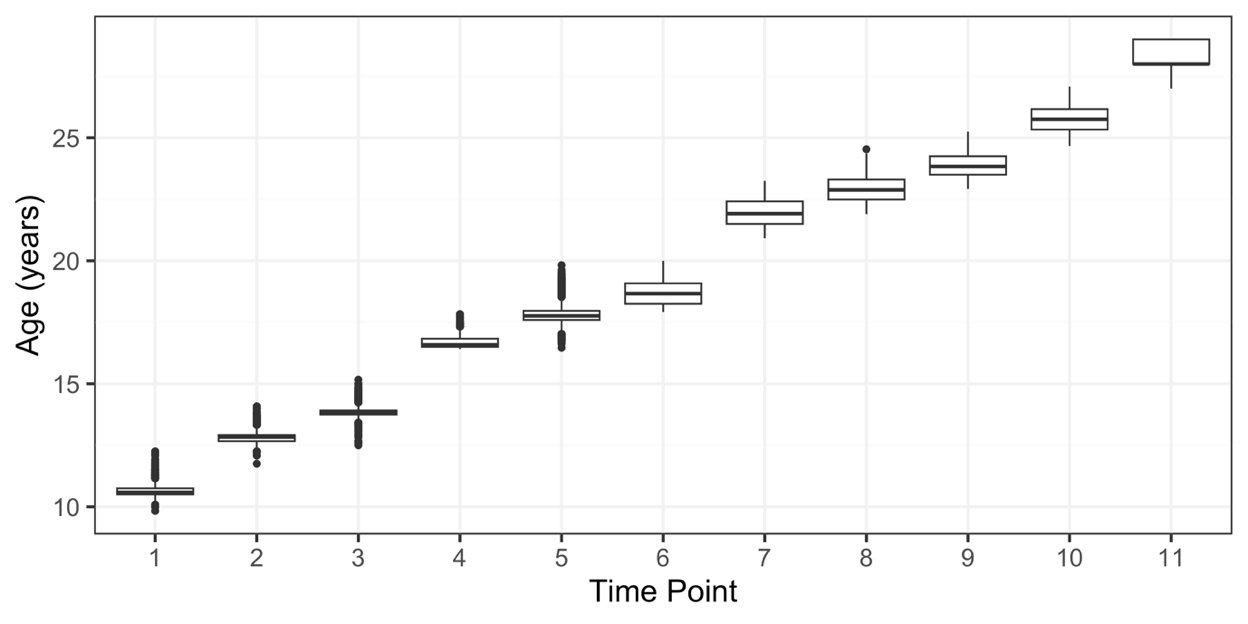


Supplementary Figure 2. Box plot of ages of individuals at each appointment time point in UK Biobank.
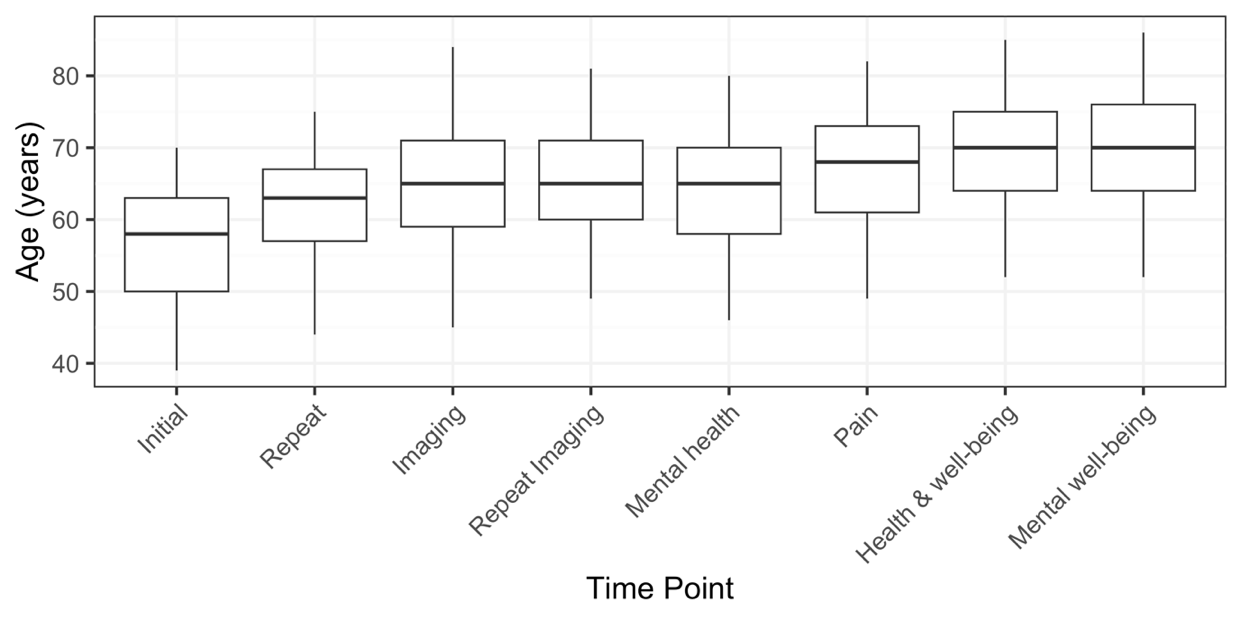


Supplementary Figure 3. Plots of covariates per IL-6 tertile group in ALSPAC. A. Number of females and males, B. Maternal education, C. BMI, D. log transformed BMI. Abbreviations: A-level/Degree, A/D; CSE/O-level/Vocational Qualification, C/O/N.


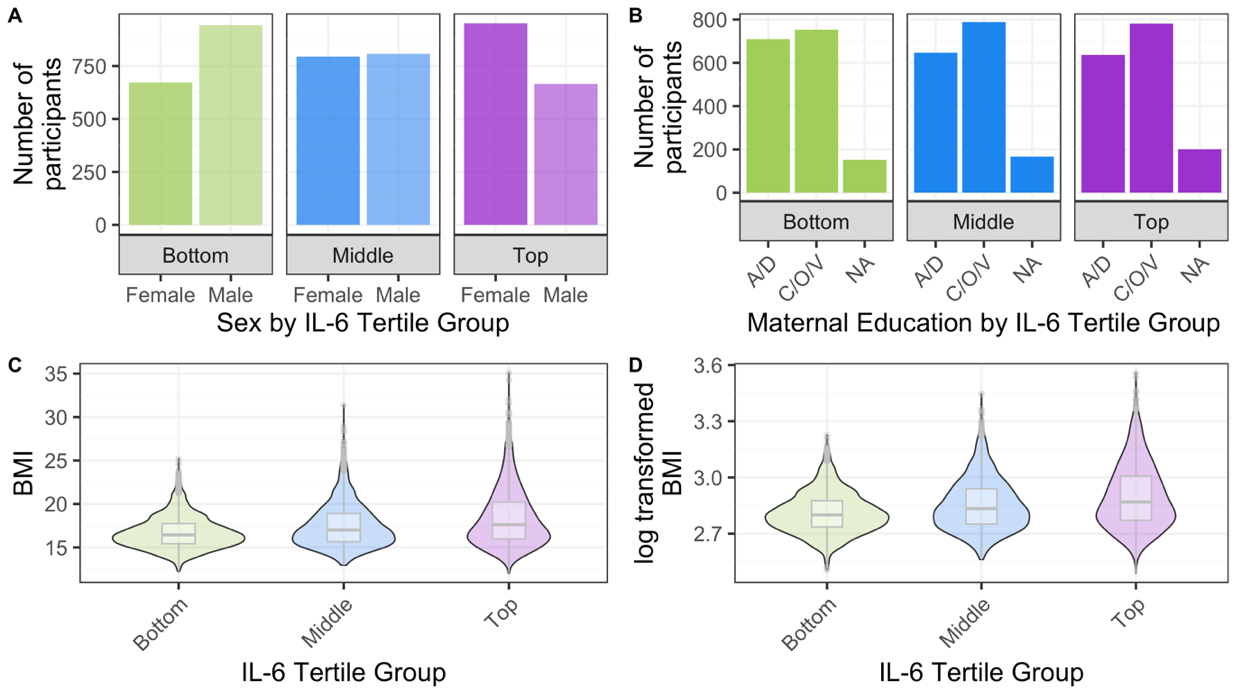


Supplementary Figure 4. Plots of covariates per IL-6 tertile group in UK Biobank. A. Number of females and males, B. Townsend deprivation index, C. Smoking status, D. BMI, E. log transformed BMI.


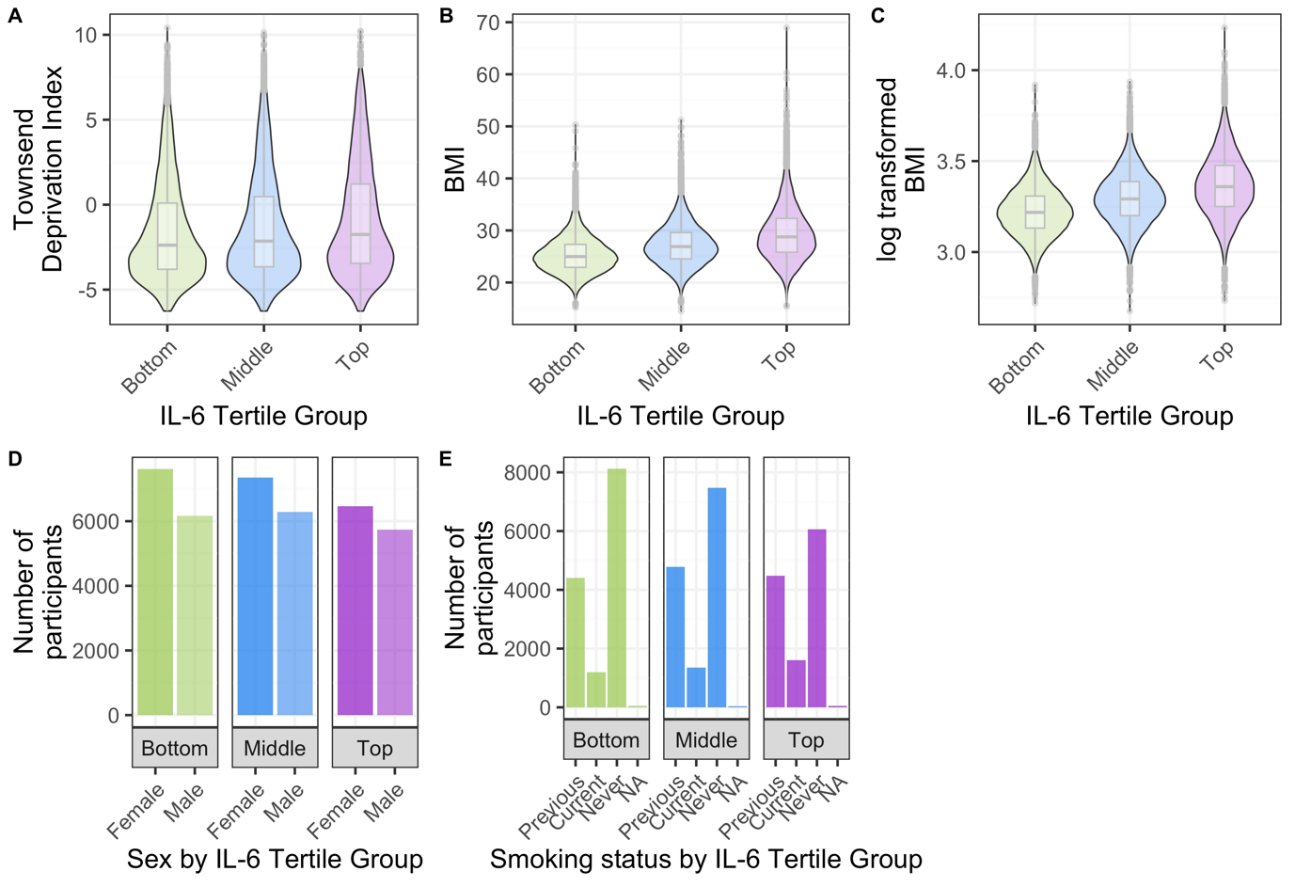


Supplementary Figure 5. Alluvial plot of Townsend deprivation index quintile in ALSPAC for three time points.


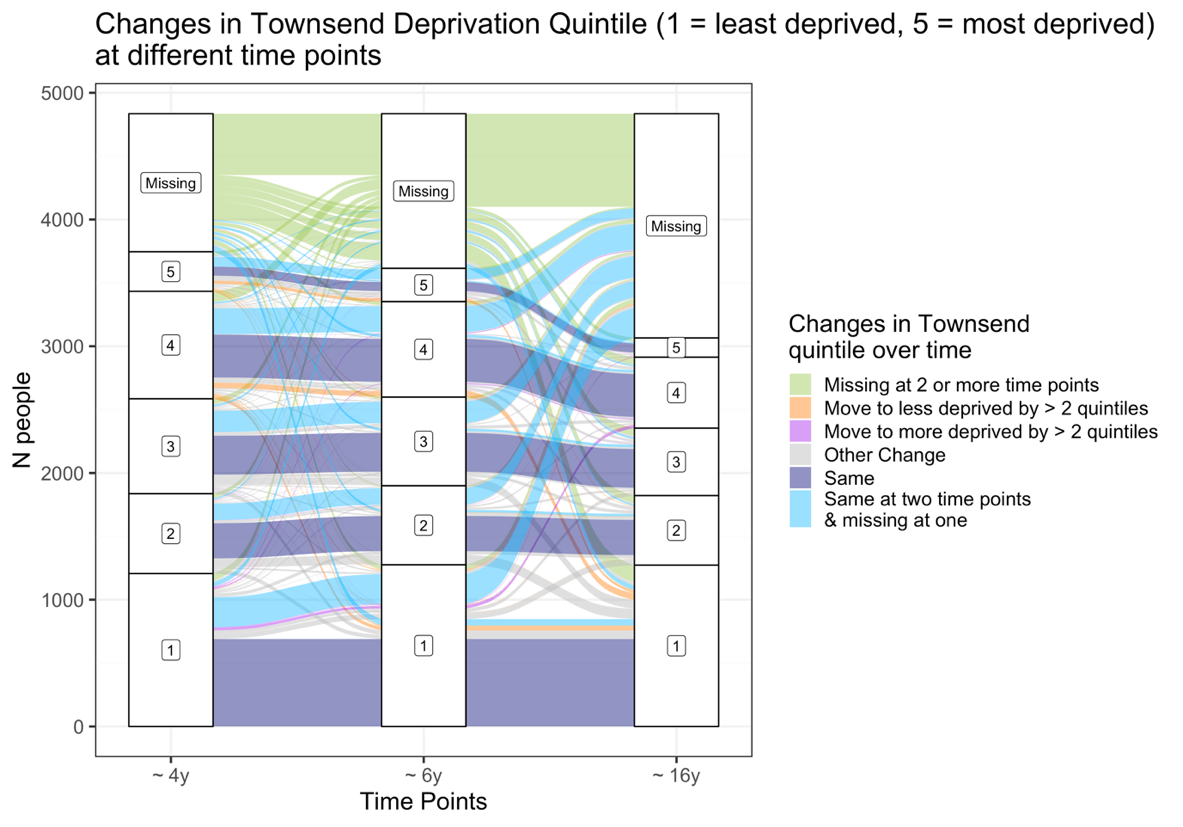


Supplementary Figure 6. Number of completed SMFQ questionnaires in ALSPAC. Coloured by IL-6 tertile.


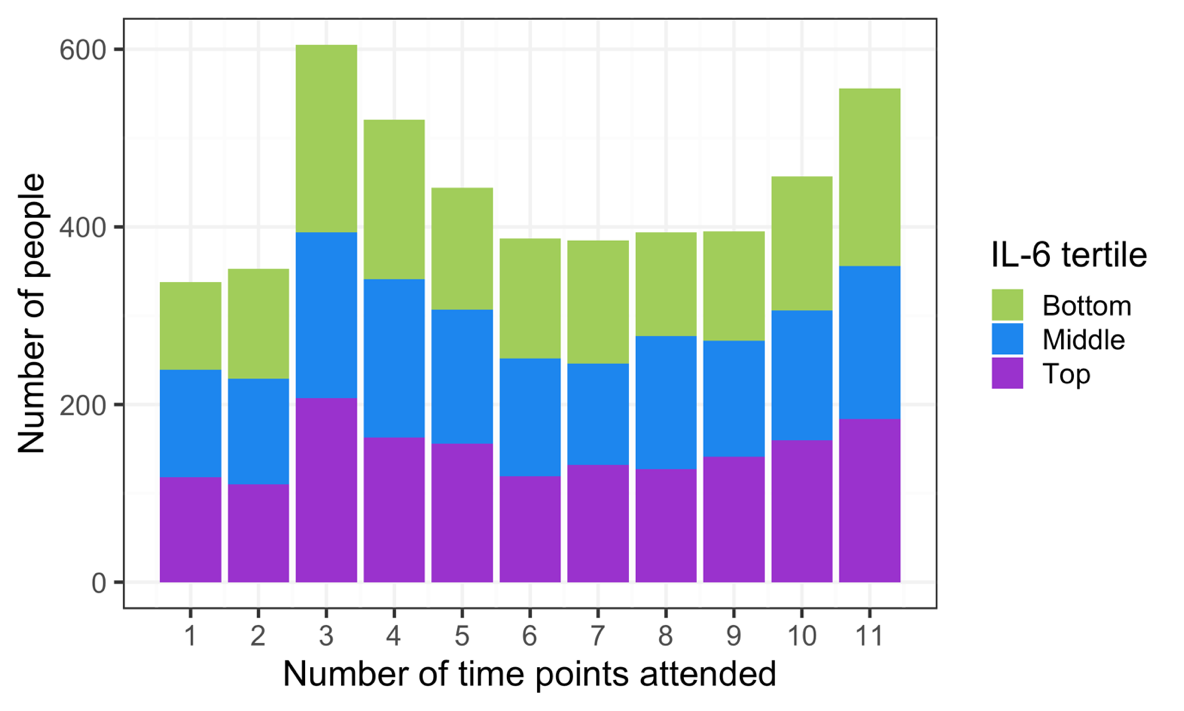


Supplementary Figure 7. Number of people who attended only one or at least two assessments in ALSPAC. Coloured by IL-6 tertile.


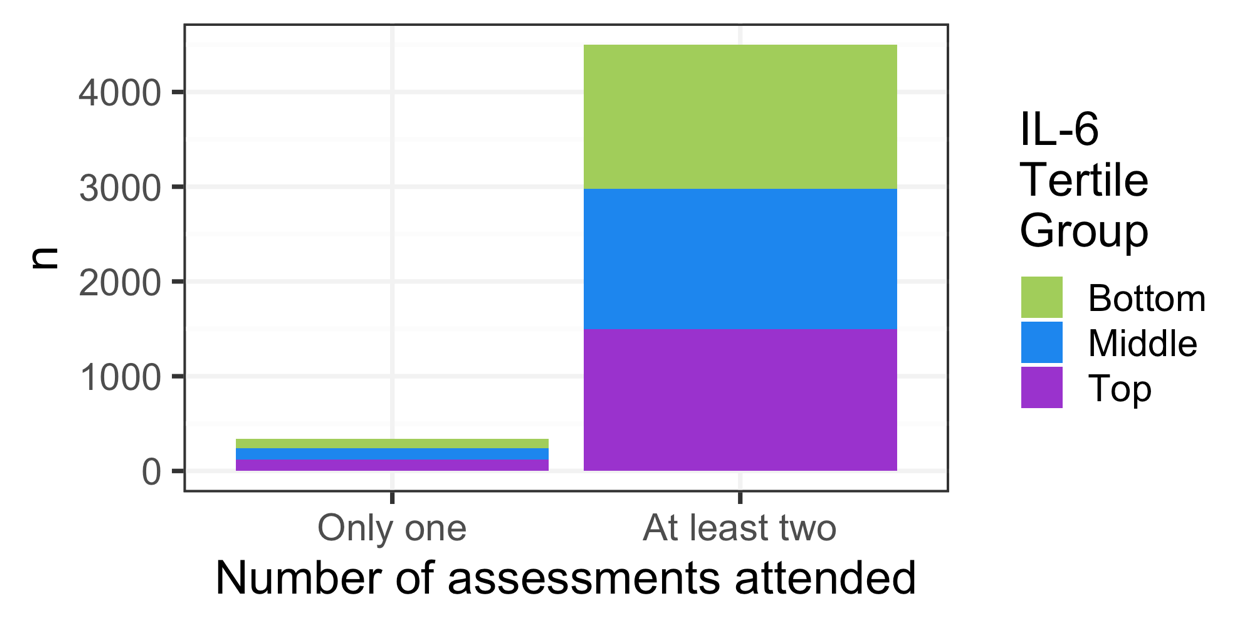


Supplementary Figure 8. Number of completed PHQ-2 questionnaires in UKB. Coloured by IL-6 tertile. Participants subset to those that remained alive after the initial assessment. Excluding the two imaging appointments as only a subset of participants were invited to these appointments.


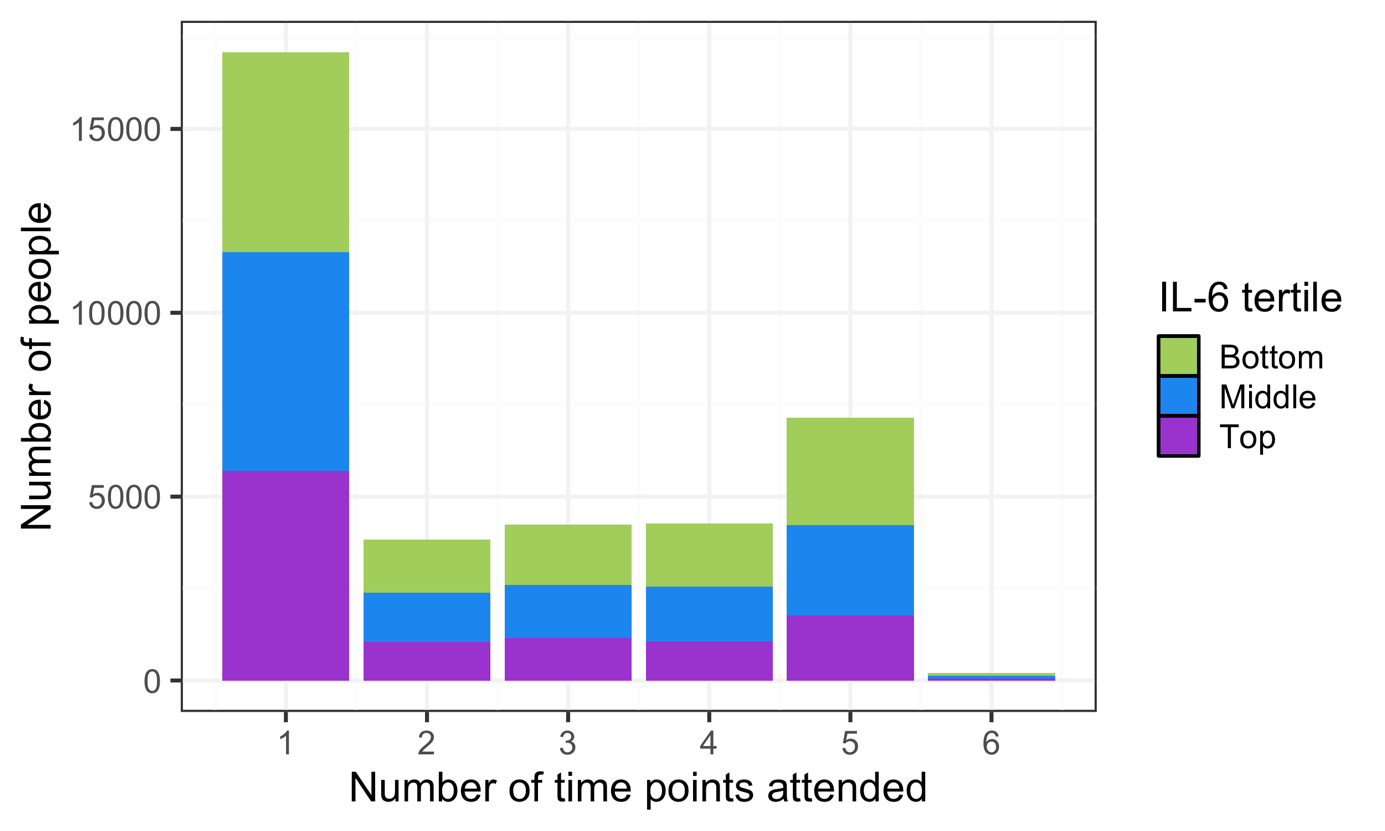


Supplementary Figure 9. Number of completed PHQ-2 questionnaires and if person was dead or alive in UK Biobank.


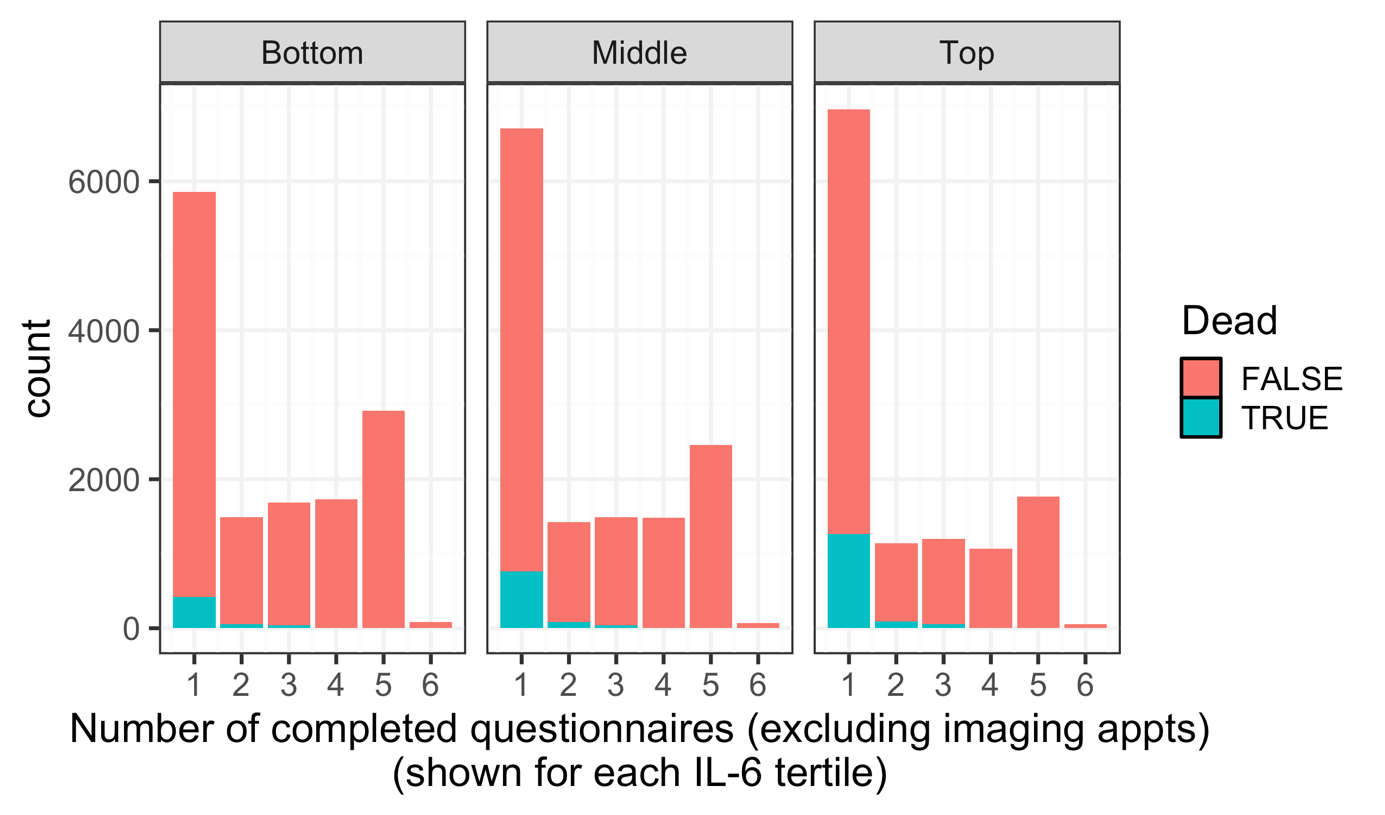


Supplementary Figure 10. Number of people who attended only one or at least two assessments in UK Biobank. Coloured by IL-6 tertile.


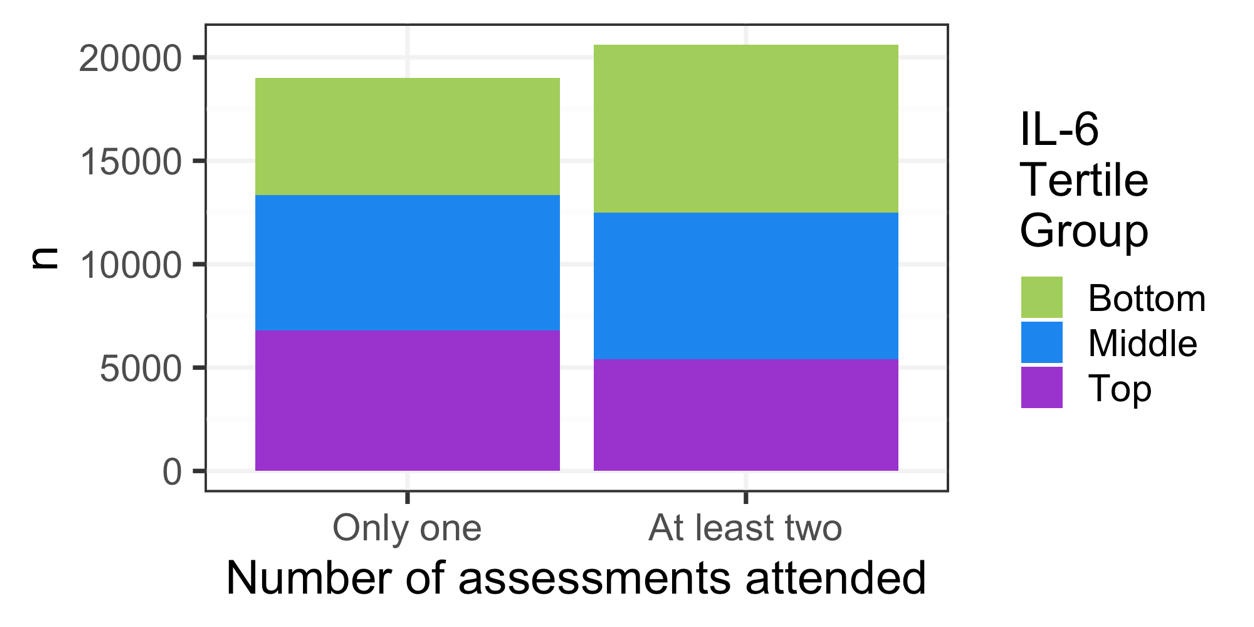


**Supplementary Tables**

Supplementary Table 1. Number of participants by age in ALSPAC.

| Time point | Age (years) | N |
| --- | --- | --- |
| 1 | 10 | 1952 |
| 1 | 11 | 2551 |
| 1 | 12 | 59 |
| 2 | 12 | 407 |
| 2 | 13 | 3654 |
| 2 | 14 | 48 |
| 3 | 12 | N < 5 |
| 3 | 13 | 31 |
| 3 | 14 | 3670 |
| 3 | 15 | 35 |
| 4 | 16 | 1244 |
| 4 | 17 | 1539 |
| 4 | 18 | 14 |
| 5 | 16 | N < 5 |
| 5 | 17 | 395 |
| 5 | 18 | 2039 |
| 5 | 19 | 147 |
| 5 | 20 | N < 5 |
| 6 | 18 | 811 |
| 6 | 19 | 972 |
| 6 | 20 | 66 |
| 7 | 21 | 380 |
| 7 | 22 | 1192 |
| 7 | 23 | 326 |
| 8 | 22 | 543 |
| 8 | 23 | 1271 |
| 8 | 24 | 296 |
| 8 | 25 | N < 5 |
| 9 | 23 | 514 |
| 9 | 24 | 1380 |
| 9 | 25 | 242 |
| 10 | 25 | 697 |
| 10 | 26 | 1428 |
| 10 | 27 | 136 |
| 11 | 27 | 52 |
| 11 | 28 | 1293 |
| 11 | 29 | 903 |

Supplementary Table 2. Number of participants by age in UK Biobank.

| Time point | Age (years) | N |
| --- | --- | --- |
| Initial | 39 | N < 5 |
| Initial | 40 | 474 |
| Initial | 41 | 877 |
| Initial | 42 | 845 |
| Initial | 43 | 889 |
| Initial | 44 | 949 |
| Initial | 45 | 1038 |
| Initial | 46 | 993 |
| Initial | 47 | 978 |
| Initial | 48 | 979 |
| Initial | 49 | 1080 |
| Initial | 50 | 1081 |
| Initial | 51 | 1191 |
| Initial | 52 | 1182 |
| Initial | 53 | 1207 |
| Initial | 54 | 1259 |
| Initial | 55 | 1323 |
| Initial | 56 | 1394 |
| Initial | 57 | 1385 |
| Initial | 58 | 1476 |
| Initial | 59 | 1563 |
| Initial | 60 | 1959 |
| Initial | 61 | 2085 |
| Initial | 62 | 2033 |
| Initial | 63 | 1907 |
| Initial | 64 | 1772 |
| Initial | 65 | 1751 |
| Initial | 66 | 1599 |
| Initial | 67 | 1497 |
| Initial | 68 | 1351 |
| Initial | 69 | 1299 |
| Initial | 70 | 196 |
| Repeat | 44 | N < 5 |
| Repeat | 45 | 26 |
| Repeat | 46 | 22 |
| Repeat | 47 | 27 |
| Repeat | 48 | 24 |
| Repeat | 49 | 38 |
| Repeat | 50 | 37 |
| Repeat | 51 | 34 |
| Repeat | 52 | 32 |
| Repeat | 53 | 34 |
| Repeat | 54 | 44 |
| Repeat | 55 | 39 |
| Repeat | 56 | 47 |
| Repeat | 57 | 49 |
| Repeat | 58 | 50 |
| Repeat | 59 | 63 |
| Repeat | 60 | 72 |
| Repeat | 61 | 70 |
| Repeat | 62 | 99 |
| Repeat | 63 | 84 |
| Repeat | 64 | 104 |
| Repeat | 65 | 137 |
| Repeat | 66 | 107 |
| Repeat | 67 | 80 |
| Repeat | 68 | 82 |
| Repeat | 69 | 60 |
| Repeat | 70 | 58 |
| Repeat | 71 | 51 |
| Repeat | 72 | 46 |
| Repeat | 73 | 29 |
| Repeat | 74 | 15 |
| Repeat | 75 | N < 5 |
| Imaging | 45 | N < 5 |
| Imaging | 46 | 8 |
| Imaging | 47 | 15 |
| Imaging | 48 | 30 |
| Imaging | 49 | 44 |
| Imaging | 50 | 66 |
| Imaging | 51 | 82 |
| Imaging | 52 | 85 |
| Imaging | 53 | 115 |
| Imaging | 54 | 120 |
| Imaging | 55 | 143 |
| Imaging | 56 | 146 |
| Imaging | 57 | 159 |
| Imaging | 58 | 157 |
| Imaging | 59 | 158 |
| Imaging | 60 | 159 |
| Imaging | 61 | 190 |
| Imaging | 62 | 180 |
| Imaging | 63 | 187 |
| Imaging | 64 | 208 |
| Imaging | 65 | 197 |
| Imaging | 66 | 204 |
| Imaging | 67 | 222 |
| Imaging | 68 | 211 |
| Imaging | 69 | 236 |
| Imaging | 70 | 211 |
| Imaging | 71 | 237 |
| Imaging | 72 | 203 |
| Imaging | 73 | 175 |
| Imaging | 74 | 145 |
| Imaging | 75 | 164 |
| Imaging | 76 | 93 |
| Imaging | 77 | 75 |
| Imaging | 78 | 74 |
| Imaging | 79 | 63 |
| Imaging | 80 | 34 |
| Imaging | 81 | 19 |
| Imaging | 82 | 8 |
| Imaging | 83 | N < 5 |
| Imaging | 84 | N < 5 |
| Repeat Imaging | 49 | N < 5 |
| Repeat Imaging | 50 | N < 5 |
| Repeat Imaging | 51 | N < 5 |
| Repeat Imaging | 52 | 5 |
| Repeat Imaging | 53 | 5 |
| Repeat Imaging | 54 | 14 |
| Repeat Imaging | 55 | 5 |
| Repeat Imaging | 56 | 19 |
| Repeat Imaging | 57 | 12 |
| Repeat Imaging | 58 | 8 |
| Repeat Imaging | 59 | 9 |
| Repeat Imaging | 60 | 15 |
| Repeat Imaging | 61 | 19 |
| Repeat Imaging | 62 | 9 |
| Repeat Imaging | 63 | 14 |
| Repeat Imaging | 64 | 20 |
| Repeat Imaging | 65 | 9 |
| Repeat Imaging | 66 | 15 |
| Repeat Imaging | 67 | 13 |
| Repeat Imaging | 68 | 19 |
| Repeat Imaging | 69 | 18 |
| Repeat Imaging | 70 | 11 |
| Repeat Imaging | 71 | 15 |
| Repeat Imaging | 72 | 21 |
| Repeat Imaging | 73 | 9 |
| Repeat Imaging | 74 | 9 |
| Repeat Imaging | 75 | 7 |
| Repeat Imaging | 76 | 7 |
| Repeat Imaging | 77 | 7 |
| Repeat Imaging | 78 | 5 |
| Repeat Imaging | 79 | 6 |
| Repeat Imaging | 80 | N < 5 |
| Repeat Imaging | 81 | N < 5 |
| Mental health | 46 | N < 5 |
| Mental health | 47 | 56 |
| Mental health | 48 | 120 |
| Mental health | 49 | 210 |
| Mental health | 50 | 284 |
| Mental health | 51 | 318 |
| Mental health | 52 | 298 |
| Mental health | 53 | 333 |
| Mental health | 54 | 361 |
| Mental health | 55 | 306 |
| Mental health | 56 | 339 |
| Mental health | 57 | 330 |
| Mental health | 58 | 376 |
| Mental health | 59 | 400 |
| Mental health | 60 | 406 |
| Mental health | 61 | 423 |
| Mental health | 62 | 485 |
| Mental health | 63 | 496 |
| Mental health | 64 | 472 |
| Mental health | 65 | 496 |
| Mental health | 66 | 538 |
| Mental health | 67 | 572 |
| Mental health | 68 | 655 |
| Mental health | 69 | 783 |
| Mental health | 70 | 667 |
| Mental health | 71 | 540 |
| Mental health | 72 | 541 |
| Mental health | 73 | 478 |
| Mental health | 74 | 407 |
| Mental health | 75 | 335 |
| Mental health | 76 | 287 |
| Mental health | 77 | 209 |
| Mental health | 78 | 87 |
| Mental health | 79 | 15 |
| Mental health | 80 | N < 5 |
| Pain | 49 | N < 5 |
| Pain | 50 | 48 |
| Pain | 51 | 150 |
| Pain | 52 | 252 |
| Pain | 53 | 309 |
| Pain | 54 | 317 |
| Pain | 55 | 326 |
| Pain | 56 | 344 |
| Pain | 57 | 362 |
| Pain | 58 | 336 |
| Pain | 59 | 367 |
| Pain | 60 | 358 |
| Pain | 61 | 445 |
| Pain | 62 | 416 |
| Pain | 63 | 451 |
| Pain | 64 | 461 |
| Pain | 65 | 525 |
| Pain | 66 | 530 |
| Pain | 67 | 487 |
| Pain | 68 | 575 |
| Pain | 69 | 565 |
| Pain | 70 | 627 |
| Pain | 71 | 706 |
| Pain | 72 | 793 |
| Pain | 73 | 670 |
| Pain | 74 | 500 |
| Pain | 75 | 599 |
| Pain | 76 | 471 |
| Pain | 77 | 412 |
| Pain | 78 | 325 |
| Pain | 79 | 278 |
| Pain | 80 | 183 |
| Pain | 81 | 78 |
| Pain | 82 | 14 |
| Health & well-being | 52 | N < 5 |
| Health & well-being | 53 | 65 |
| Health & well-being | 54 | 170 |
| Health & well-being | 55 | 274 |
| Health & well-being | 56 | 360 |
| Health & well-being | 57 | 391 |
| Health & well-being | 58 | 420 |
| Health & well-being | 59 | 423 |
| Health & well-being | 60 | 445 |
| Health & well-being | 61 | 409 |
| Health & well-being | 62 | 420 |
| Health & well-being | 63 | 452 |
| Health & well-being | 64 | 532 |
| Health & well-being | 65 | 543 |
| Health & well-being | 66 | 554 |
| Health & well-being | 67 | 584 |
| Health & well-being | 68 | 646 |
| Health & well-being | 69 | 636 |
| Health & well-being | 70 | 620 |
| Health & well-being | 71 | 681 |
| Health & well-being | 72 | 674 |
| Health & well-being | 73 | 712 |
| Health & well-being | 74 | 797 |
| Health & well-being | 75 | 908 |
| Health & well-being | 76 | 788 |
| Health & well-being | 77 | 572 |
| Health & well-being | 78 | 630 |
| Health & well-being | 79 | 509 |
| Health & well-being | 80 | 427 |
| Health & well-being | 81 | 352 |
| Health & well-being | 82 | 270 |
| Health & well-being | 83 | 186 |
| Health & well-being | 84 | 81 |
| Health & well-being | 85 | 11 |
| Mental well-being | 52 | 5 |
| Mental well-being | 53 | 35 |
| Mental well-being | 54 | 132 |
| Mental well-being | 55 | 208 |
| Mental well-being | 56 | 295 |
| Mental well-being | 57 | 327 |
| Mental well-being | 58 | 345 |
| Mental well-being | 59 | 380 |
| Mental well-being | 60 | 388 |
| Mental well-being | 61 | 368 |
| Mental well-being | 62 | 405 |
| Mental well-being | 63 | 414 |
| Mental well-being | 64 | 478 |
| Mental well-being | 65 | 487 |
| Mental well-being | 66 | 469 |
| Mental well-being | 67 | 525 |
| Mental well-being | 68 | 580 |
| Mental well-being | 69 | 557 |
| Mental well-being | 70 | 544 |
| Mental well-being | 71 | 594 |
| Mental well-being | 72 | 614 |
| Mental well-being | 73 | 614 |
| Mental well-being | 74 | 737 |
| Mental well-being | 75 | 798 |
| Mental well-being | 76 | 704 |
| Mental well-being | 77 | 556 |
| Mental well-being | 78 | 527 |
| Mental well-being | 79 | 473 |
| Mental well-being | 80 | 374 |
| Mental well-being | 81 | 286 |
| Mental well-being | 82 | 259 |
| Mental well-being | 83 | 169 |
| Mental well-being | 84 | 76 |
| Mental well-being | 85 | 19 |
| Mental well-being | 86 | N < 5 |

Supplementary Table 3. Descriptive statistics of SMFQ responses and ages at each time point in ALSPAC

Supplementary Table 4. Questions in the SMFQ.

| Question Number | SMFQ Question: |
| --- | --- |
| 1 | I felt miserable or unhappy |
| 2 | I didn’t enjoy anything at all |
| 3 | I felt so tired I just sat around and did nothing |
| 4 | I was very restless |
| 5 | I felt I was no good anymore |
| 6 | I cried a lot |
| 7 | I found it hard to think properly or concentrate |
| 8 | I hated myself |
| 9 | I was a bad person |
| 10 | I felt lonely |
| 11 | I thought nobody really loved me |
| 12 | I thought I could never be as good as others |
| 13 | I did everything wrong |

Supplementary Table 5. PHQ-9 items available at each time point. Data available is shown as “T” for “TRUE” and data not available is shown as “F” for “FALSE” in the table.

Supplementary Table 6. Descriptive statistics of PHQ-2 responses and ages at each time point in UK Biobank

Supplementary Table 7. Estimates of linear, quadratic, cubic and quartic models to assess best model fit in ALSPAC

Supplementary Table 8. Estimates of linear and quadratic models to assess best model fit in UK Biobank

| Parameter | Estimate (Linear) | SE (Linear) | p-value (Linear) | N (Linear) | Estimate (Quadratic) | SE (Quadratic) | p-value (Quadratic) | N (Quadratic) |
| --- | --- | --- | --- | --- | --- | --- | --- | --- |
| Intercept | 0.6982 | 0.011 | <0.0001 | 39613 | 0.7988 | 0.0174 | <0.0001 | 39613 |
| age | <0.0001 | 0.0004 | <0.0001 | 39613 | <0.0001 | 0.0015 | <0.0001 | 39613 |
| age^2 (acceleration) | - | - | - | - | 0.0002 | <0.0001 | <0.0001 | 39613 |
| Intercept variance | 1.3155 | 1.1469 | - | - | 1.2897 | 1.1356 | - | - |
| Age (slope) variance | 0.0011 | 0.0335 | - | - | 0.0011 | 0.0332 | - | - |
| Intercept/age covariance | -0.03 | -0.7821 | - | - | -0.0292 | -0.7757 | - | - |
| Residual variance | 0.5897 | 0.7679 | - | - | 0.5898 | 0.768 | - | - |
| Deviance | 283639.095 |  |  |  | 283584.272 |  |  |  |
| AIC | 283651.095 |  |  |  | 283598.272 |  |  |  |
| BIC | 283708.249 |  |  |  | 283664.951 |  |  |  |

Supplementary Table 9. Counts of each anti-inflammatory medication taken in UK Biobank.

Supplementary Table 10. Counts and types of inflammatory conditions in UK Biobank.

Supplementary Table 11. Estimated depression scores for each IL-6 tertile trajectory at ages 10, 13, 16, 19, 22, 25 and 28 years, in ALSPAC.

| IL-6 Tertile Group | age | estimate | 95% CI |
| --- | --- | --- | --- |
| Score [IL-6 tertile = Bottom] | 10 | 1.119 | -0.815 - 3.053 |
| Score [IL-6 tertile = Middle] | 10 | 0.976 | -0.989 - 2.941 |
| Score [IL-6 tertile = Top] | 10 | 0.847 | -1.147 - 2.84 |
| Score [IL-6 tertile = Bottom] | 13 | 1.395 | -0.522 - 3.312 |
| Score [IL-6 tertile = Middle] | 13 | 1.405 | -0.541 - 3.35 |
| Score [IL-6 tertile = Top] | 13 | 1.805 | -0.169 - 3.779 |
| Score [IL-6 tertile = Bottom] | 16 | 2.444 | 0.525 - 4.363 |
| Score [IL-6 tertile = Middle] | 16 | 2.554 | 0.607 - 4.502 |
| Score [IL-6 tertile = Top] | 16 | 3.017 | 1.04 - 4.994 |
| Score [IL-6 tertile = Bottom] | 19 | 3.221 | 1.295 - 5.148 |
| Score [IL-6 tertile = Middle] | 19 | 3.346 | 1.391 - 5.3 |
| Score [IL-6 tertile = Top] | 19 | 3.737 | 1.752 - 5.721 |
| Score [IL-6 tertile = Bottom] | 22 | 3.355 | 1.427 - 5.284 |
| Score [IL-6 tertile = Middle] | 22 | 3.384 | 1.427 - 5.34 |
| Score [IL-6 tertile = Top] | 22 | 3.764 | 1.777 - 5.751 |
| Score [IL-6 tertile = Bottom] | 25 | 3.152 | 1.218 - 5.086 |
| Score [IL-6 tertile = Middle] | 25 | 2.956 | 0.994 - 4.918 |
| Score [IL-6 tertile = Top] | 25 | 3.443 | 1.451 - 5.435 |
| Score [IL-6 tertile = Bottom] | 28 | 3.59 | 1.643 - 5.536 |
| Score [IL-6 tertile = Middle] | 28 | 3.035 | 1.061 - 5.009 |
| Score [IL-6 tertile = Top] | 28 | 3.668 | 1.664 - 5.672 |

Supplementary Table 12. Model estimates for main analysis in ALSPAC. IL-6 as a categorical variable (tertiles).

|  | Unadjusted | | | | Sex Adjusted | | | | Fully Adjusted | | | |
| --- | --- | --- | --- | --- | --- | --- | --- | --- | --- | --- | --- | --- |
| Parameter | Estimate | SE | p-value | N | Estimate | SE | p-value | N | Estimate | SE | p-value | N |
| Intercept | 3.8468 | 0.1581 | <0.0001 | 4835 | 3.4667 | 0.1563 | <0.0001 | 4835 | 1.1192 | 0.9868 | 0.2567 | 4264 |
| age | <0.0001 | 0.1145 | 0.1212 | 4835 | <0.0001 | 0.1156 | 0.1213 | 4835 | <0.0001 | 0.12 | 0.0807 | 4264 |
| age^2 (acceleration) | 0.1302 | 0.026 | <0.0001 | 4835 | 0.1293 | 0.0265 | <0.0001 | 4835 | 0.1355 | 0.0274 | <0.0001 | 4264 |
| age^3 (cubic change) | <0.0001 | 0.0021 | <0.0001 | 4835 | <0.0001 | 0.0022 | <0.0001 | 4835 | <0.0001 | 0.0022 | <0.0001 | 4264 |
| age^4 (quartic change) | 0.0003 | <0.0001 | <0.0001 | 4835 | 0.0003 | <0.0001 | <0.0001 | 4835 | 0.0003 | <0.0001 | <0.0001 | 4264 |
| IL6_tertileMiddle | 0.0327 | 0.2249 | 0.8845 | 4835 | <0.0001 | 0.2161 | 0.8284 | 4835 | <0.0001 | 0.2356 | 0.5427 | 4264 |
| IL6_tertileTop | 0.0287 | 0.2245 | 0.8984 | 4835 | <0.0001 | 0.2162 | 0.541 | 4835 | <0.0001 | 0.238 | 0.2522 | 4264 |
| age:IL6_tertileMiddle | 0.0106 | 0.1634 | 0.9484 | 4835 | 0.0168 | 0.1651 | 0.9187 | 4835 | 0.0555 | 0.1716 | 0.7462 | 4264 |
| age:IL6_tertileTop | 0.3012 | 0.1628 | 0.0643 | 4835 | 0.3045 | 0.1644 | 0.064 | 4835 | 0.3581 | 0.1717 | 0.037 | 4264 |
| age^2 (acceleration):IL6_tertileMiddle | 0.008 | 0.0371 | 0.83 | 4835 | 0.0062 | 0.0377 | 0.8705 | 4835 | <0.0001 | 0.039 | 0.9865 | 4264 |
| age^2 (acceleration):IL6_tertileTop | <0.0001 | 0.0369 | 0.277 | 4835 | <0.0001 | 0.0376 | 0.2784 | 4835 | <0.0001 | 0.039 | 0.1821 | 4264 |
| age^3 (cubic change):IL6_tertileMiddle | <0.0001 | 0.003 | 0.7455 | 4835 | <0.0001 | 0.0031 | 0.7877 | 4835 | <0.0001 | 0.0032 | 0.9293 | 4264 |
| age^3 (cubic change):IL6_tertileTop | 0.0021 | 0.003 | 0.4793 | 4835 | 0.0022 | 0.0031 | 0.4749 | 4835 | 0.003 | 0.0032 | 0.3419 | 4264 |
| age^4 (quartic change):IL6_tertileMiddle | <0.0001 | <0.0001 | 0.7705 | 4835 | <0.0001 | <0.0001 | 0.8114 | 4835 | <0.0001 | <0.0001 | 0.9589 | 4264 |
| age^4 (quartic change):IL6_tertileTop | <0.0001 | <0.0001 | 0.5873 | 4835 | <0.0001 | <0.0001 | 0.5761 | 4835 | <0.0001 | <0.0001 | 0.4344 | 4264 |
| Sex1 | - | - | - | - | 0.9212 | 0.0903 | <0.0001 | 4835 | 0.9241 | 0.0976 | <0.0001 | 4264 |
| Maternal.education.at.birth1 | - | - | - | - | - | - | - | - | <0.0001 | 0.0967 | 0.162 | 4264 |
| BMI_age9_log | - | - | - | - | - | - | - | - | 0.8621 | 0.3458 | 0.0127 | 4264 |
| Intercept variance | 10.3034 | 3.2099 | - | - | 6.0457 | 2.4588 | - | - | 9.3427 | 3.0566 | - | - |
| Age (slope) variance | 0.8899 | 0.9433 | - | - | 0.5284 | 0.7269 | - | - | 0.7297 | 0.8542 | - | - |
| Quadratic variance | 0.0021 | 0.0458 | - | - | 0.0012 | 0.0353 | - | - | 0.0017 | 0.0412 | - | - |
| Intercept/age covariance | -1.6773 | -0.5539 | - | - | -0.6783 | -0.3795 | - | - | -1.4896 | -0.5705 | - | - |
| Intercept/Quadratic covariance | 0.0718 | 0.4881 | - | - | 0.0258 | 0.2973 | - | - | 0.0633 | 0.5025 | - | - |
| Age (slope)/Quadratic covariance | -0.0413 | -0.9554 | - | - | -0.0238 | -0.9291 | - | - | -0.0333 | -0.9469 | - | - |
| Residual variance | 12.2383 | 3.4983 | - | - | 12.922 | 3.5947 | - | - | 12.3976 | 3.521 | - | - |
| Deviance | 170654 |  |  |  | 170248 |  |  |  | 153089 |  |  |  |
| AIC | 170698 |  |  |  | 170294 |  |  |  | 153139 |  |  |  |
| BIC | 170880 |  |  |  | 170485 |  |  |  | 153344 |  |  |  |

Supplementary Table 13. Model estimates for main analysis in ALSPAC split by sex. A variable was created that split the IL-6 tertiles by sex: female & bottom third IL-6 tertile, female & middle third IL-6 tertile, female & top third IL-6 tertile, male & bottom third IL-6 tertile, male & middle third IL-6 tertile and male & top third IL-6 tertile. The models were then run splitting the trajectories on this sex-split IL-6 tertile variable.

|  | Unadjusted | | | | Fully Adjusted | | | |
| --- | --- | --- | --- | --- | --- | --- | --- | --- |
| Parameter | Estimate | SE | p-value | N | Estimate | SE | p-value | N |
| Intercept | 4.2455 | 0.2069 | <0.0001 | 4835 | 2.0475 | 0.9742 | 0.0356 | 4264 |
| age | <0.0001 | 0.1538 | 0.0004 | 4835 | <0.0001 | 0.1608 | 0.0002 | 4264 |
| age^2 (acceleration) | 0.1664 | 0.0359 | <0.0001 | 4835 | 0.1775 | 0.0376 | <0.0001 | 4264 |
| age^3 (cubic change) | <0.0001 | 0.003 | <0.0001 | 4835 | <0.0001 | 0.0031 | <0.0001 | 4264 |
| age^4 (quartic change) | 0.0003 | <0.0001 | <0.0001 | 4835 | 0.0004 | <0.0001 | <0.0001 | 4264 |
| IL6_sex_tertileMale_Middle | 0.1836 | 0.3064 | 0.5491 | 4835 | 0.0102 | 0.309 | 0.9738 | 4264 |
| IL6_sex_tertileMale_Top | 0.0669 | 0.3248 | 0.8369 | 4835 | <0.0001 | 0.3275 | 0.7313 | 4264 |
| IL6_sex_tertileFemale_Bottom | <0.0001 | 0.3189 | 0.0026 | 4835 | <0.0001 | 0.321 | 0.0006 | 4264 |
| IL6_sex_tertileFemale_Middle | <0.0001 | 0.3053 | 0.0026 | 4835 | <0.0001 | 0.3079 | 0.0008 | 4264 |
| IL6_sex_tertileFemale_Top | <0.0001 | 0.2914 | 0.0194 | 4835 | <0.0001 | 0.2977 | 0.0035 | 4264 |
| age:IL6_sex_tertileMale_Middle | <0.0001 | 0.2291 | 0.2013 | 4835 | <0.0001 | 0.2403 | 0.3772 | 4264 |
| age:IL6_sex_tertileMale_Top | 0.0696 | 0.2429 | 0.7746 | 4835 | 0.1955 | 0.2545 | 0.4423 | 4264 |
| age:IL6_sex_tertileFemale_Bottom | 0.9098 | 0.2311 | <0.0001 | 4835 | 0.9872 | 0.2426 | <0.0001 | 4264 |
| age:IL6_sex_tertileFemale_Middle | 1.0771 | 0.2229 | <0.0001 | 4835 | 1.135 | 0.2338 | <0.0001 | 4264 |
| age:IL6_sex_tertileFemale_Top | 1.1256 | 0.213 | <0.0001 | 4835 | 1.1745 | 0.2253 | <0.0001 | 4264 |
| age^2 (acceleration):IL6_sex_tertileMale_Middle | 0.0553 | 0.0534 | 0.3003 | 4835 | 0.0455 | 0.0561 | 0.4172 | 4264 |
| age^2 (acceleration):IL6_sex_tertileMale_Top | <0.0001 | 0.0567 | 0.693 | 4835 | <0.0001 | 0.0596 | 0.4318 | 4264 |
| age^2 (acceleration):IL6_sex_tertileFemale_Bottom | <0.0001 | 0.0526 | 0.0437 | 4835 | <0.0001 | 0.0554 | 0.0291 | 4264 |
| age^2 (acceleration):IL6_sex_tertileFemale_Middle | <0.0001 | 0.0508 | 0.0098 | 4835 | <0.0001 | 0.0534 | 0.006 | 4264 |
| age^2 (acceleration):IL6_sex_tertileFemale_Top | <0.0001 | 0.0487 | 0.0056 | 4835 | <0.0001 | 0.0516 | 0.005 | 4264 |
| age^3 (cubic change):IL6_sex_tertileMale_Middle | <0.0001 | 0.0045 | 0.4286 | 4835 | <0.0001 | 0.0047 | 0.4932 | 4264 |
| age^3 (cubic change):IL6_sex_tertileMale_Top | 0.0025 | 0.0047 | 0.5976 | 4835 | 0.0043 | 0.005 | 0.3833 | 4264 |
| age^3 (cubic change):IL6_sex_tertileFemale_Bottom | 0.0045 | 0.0043 | 0.2984 | 4835 | 0.0054 | 0.0045 | 0.2313 | 4264 |
| age^3 (cubic change):IL6_sex_tertileFemale_Middle | 0.0058 | 0.0042 | 0.165 | 4835 | 0.0072 | 0.0044 | 0.101 | 4264 |
| age^3 (cubic change):IL6_sex_tertileFemale_Top | 0.0059 | 0.004 | 0.1414 | 4835 | 0.0065 | 0.0043 | 0.1261 | 4264 |
| age^4 (quartic change):IL6_sex_tertileMale_Middle | <0.0001 | 0.0001 | 0.5877 | 4835 | <0.0001 | 0.0001 | 0.6026 | 4264 |
| age^4 (quartic change):IL6_sex_tertileMale_Top | <0.0001 | 0.0001 | 0.514 | 4835 | <0.0001 | 0.0001 | 0.3293 | 4264 |
| age^4 (quartic change):IL6_sex_tertileFemale_Bottom | <0.0001 | 0.0001 | 0.6413 | 4835 | <0.0001 | 0.0001 | 0.5411 | 4264 |
| age^4 (quartic change):IL6_sex_tertileFemale_Middle | <0.0001 | 0.0001 | 0.4639 | 4835 | <0.0001 | 0.0001 | 0.3025 | 4264 |
| age^4 (quartic change):IL6_sex_tertileFemale_Top | <0.0001 | 0.0001 | 0.453 | 4835 | <0.0001 | 0.0001 | 0.4209 | 4264 |
| Maternal.education.at.birth1 | - | - | - | - | <0.0001 | 0.0944 | 0.2305 | 4264 |
| BMI_age9_log | - | - | - | - | 0.8202 | 0.3378 | 0.0152 | 4264 |
| Intercept variance | 9.5418 | 3.089 | - | - | 6.0387 | 2.4574 | - | - |
| Age (slope) variance | 0.6808 | 0.8251 | - | - | 0.5107 | 0.7146 | - | - |
| Quadratic variance | 0.0016 | 0.0396 | - | - | 0.0012 | 0.0347 | - | - |
| Intercept/age covariance | -1.4006 | -0.5495 | - | - | -0.6649 | -0.3786 | - | - |
| Intercept/Quadratic covariance | 0.0579 | 0.4732 | - | - | 0.0253 | 0.296 | - | - |
| Age (slope)/Quadratic covariance | -0.0308 | -0.9416 | - | - | -0.023 | -0.9283 | - | - |
| Residual variance | 12.4505 | 3.5285 | - | - | 12.6766 | 3.5604 | - | - |
| Deviance | 170248 |  |  |  | 152726 |  |  |  |
| AIC | 170322 |  |  |  | 152804 |  |  |  |
| BIC | 170629 |  |  |  | 153124 |  |  |  |

Supplementary Table 14. Estimated depression scores for each IL-6 tertile trajectory at ages 10, 13, 16, 19, 22, 25 and 28 years, in ALSPAC, split by sex.

| IL-6 Tertile Group | age | estimate | 95% CI |
| --- | --- | --- | --- |
| Score [IL-6 tertile = Male_Bottom] | 10 | 2.047 | 0.138 - 3.957 |
| Score [IL-6 tertile = Male_Middle] | 10 | 2.058 | 0.113 - 4.002 |
| Score [IL-6 tertile = Male_Top] | 10 | 1.935 | -0.038 - 3.908 |
| Score [IL-6 tertile = Female_Bottom] | 10 | 0.952 | -0.973 - 2.876 |
| Score [IL-6 tertile = Female_Middle] | 10 | 1.019 | -0.929 - 2.967 |
| Score [IL-6 tertile = Female_Top] | 10 | 1.178 | -0.797 - 3.153 |
| Score [IL-6 tertile = Male_Bottom] | 13 | 1.499 | -0.383 - 3.381 |
| Score [IL-6 tertile = Male_Middle] | 13 | 1.201 | -0.71 - 3.112 |
| Score [IL-6 tertile = Male_Top] | 13 | 1.658 | -0.274 - 3.59 |
| Score [IL-6 tertile = Female_Bottom] | 13 | 2.418 | 0.53 - 4.306 |
| Score [IL-6 tertile = Female_Middle] | 13 | 2.738 | 0.824 - 4.652 |
| Score [IL-6 tertile = Female_Top] | 13 | 3.015 | 1.068 - 4.962 |
| Score [IL-6 tertile = Male_Bottom] | 16 | 2.257 | 0.371 - 4.144 |
| Score [IL-6 tertile = Male_Middle] | 16 | 2.026 | 0.109 - 3.944 |
| Score [IL-6 tertile = Male_Top] | 16 | 2.399 | 0.459 - 4.338 |
| Score [IL-6 tertile = Female_Bottom] | 16 | 3.815 | 1.921 - 5.708 |
| Score [IL-6 tertile = Female_Middle] | 16 | 4.148 | 2.231 - 6.066 |
| Score [IL-6 tertile = Female_Top] | 16 | 4.497 | 2.547 - 6.448 |
| Score [IL-6 tertile = Male_Bottom] | 19 | 3.061 | 1.161 - 4.962 |
| Score [IL-6 tertile = Male_Middle] | 19 | 2.942 | 1.008 - 4.876 |
| Score [IL-6 tertile = Male_Top] | 19 | 3.219 | 1.258 - 5.179 |
| Score [IL-6 tertile = Female_Bottom] | 19 | 4.546 | 2.64 - 6.453 |
| Score [IL-6 tertile = Female_Middle] | 19 | 4.805 | 2.877 - 6.734 |
| Score [IL-6 tertile = Female_Top] | 19 | 5.151 | 3.191 - 7.112 |
| Score [IL-6 tertile = Male_Bottom] | 22 | 3.332 | 1.426 - 5.237 |
| Score [IL-6 tertile = Male_Middle] | 22 | 3.169 | 1.229 - 5.108 |
| Score [IL-6 tertile = Male_Top] | 22 | 3.608 | 1.64 - 5.576 |
| Score [IL-6 tertile = Female_Bottom] | 22 | 4.558 | 2.648 - 6.468 |
| Score [IL-6 tertile = Female_Middle] | 22 | 4.712 | 2.78 - 6.644 |
| Score [IL-6 tertile = Female_Top] | 22 | 5.009 | 3.045 - 6.972 |
| Score [IL-6 tertile = Male_Bottom] | 25 | 3.173 | 1.255 - 5.091 |
| Score [IL-6 tertile = Male_Middle] | 25 | 2.735 | 0.779 - 4.691 |
| Score [IL-6 tertile = Male_Top] | 25 | 3.489 | 1.502 - 5.476 |
| Score [IL-6 tertile = Female_Bottom] | 25 | 4.333 | 2.413 - 6.252 |
| Score [IL-6 tertile = Female_Middle] | 25 | 4.321 | 2.381 - 6.26 |
| Score [IL-6 tertile = Female_Top] | 25 | 4.608 | 2.638 - 6.578 |
| Score [IL-6 tertile = Male_Bottom] | 28 | 3.371 | 1.425 - 5.317 |
| Score [IL-6 tertile = Male_Middle] | 28 | 2.481 | 0.49 - 4.471 |
| Score [IL-6 tertile = Male_Top] | 28 | 3.212 | 1.187 - 5.237 |
| Score [IL-6 tertile = Female_Bottom] | 28 | 4.894 | 2.954 - 6.835 |
| Score [IL-6 tertile = Female_Middle] | 28 | 4.532 | 2.575 - 6.49 |
| Score [IL-6 tertile = Female_Top] | 28 | 4.993 | 3.007 - 6.98 |

| IL-6 Tertile Group | age | estimate | 95% CI |
| --- | --- | --- | --- |
| Score [IL-6 tertile = Male_Bottom] | 10 | 2.047 | 0.138 - 3.957 |
| Score [IL-6 tertile = Male_Middle] | 10 | 2.058 | 0.113 - 4.002 |
| Score [IL-6 tertile = Male_Top] | 10 | 1.935 | -0.038 - 3.908 |
| Score [IL-6 tertile = Female_Bottom] | 10 | 0.952 | -0.973 - 2.876 |
| Score [IL-6 tertile = Female_Middle] | 10 | 1.019 | -0.929 - 2.967 |
| Score [IL-6 tertile = Female_Top] | 10 | 1.178 | -0.797 - 3.153 |
| Score [IL-6 tertile = Male_Bottom] | 13 | 1.499 | -0.383 - 3.381 |
| Score [IL-6 tertile = Male_Middle] | 13 | 1.201 | -0.71 - 3.112 |
| Score [IL-6 tertile = Male_Top] | 13 | 1.658 | -0.274 - 3.59 |
| Score [IL-6 tertile = Female_Bottom] | 13 | 2.418 | 0.53 - 4.306 |
| Score [IL-6 tertile = Female_Middle] | 13 | 2.738 | 0.824 - 4.652 |
| Score [IL-6 tertile = Female_Top] | 13 | 3.015 | 1.068 - 4.962 |
| Score [IL-6 tertile = Male_Bottom] | 16 | 2.257 | 0.371 - 4.144 |
| Score [IL-6 tertile = Male_Middle] | 16 | 2.026 | 0.109 - 3.944 |
| Score [IL-6 tertile = Male_Top] | 16 | 2.399 | 0.459 - 4.338 |
| Score [IL-6 tertile = Female_Bottom] | 16 | 3.815 | 1.921 - 5.708 |
| Score [IL-6 tertile = Female_Middle] | 16 | 4.148 | 2.231 - 6.066 |
| Score [IL-6 tertile = Female_Top] | 16 | 4.497 | 2.547 - 6.448 |
| Score [IL-6 tertile = Male_Bottom] | 19 | 3.061 | 1.161 - 4.962 |
| Score [IL-6 tertile = Male_Middle] | 19 | 2.942 | 1.008 - 4.876 |
| Score [IL-6 tertile = Male_Top] | 19 | 3.219 | 1.258 - 5.179 |
| Score [IL-6 tertile = Female_Bottom] | 19 | 4.546 | 2.64 - 6.453 |
| Score [IL-6 tertile = Female_Middle] | 19 | 4.805 | 2.877 - 6.734 |
| Score [IL-6 tertile = Female_Top] | 19 | 5.151 | 3.191 - 7.112 |
| Score [IL-6 tertile = Male_Bottom] | 22 | 3.332 | 1.426 - 5.237 |
| Score [IL-6 tertile = Male_Middle] | 22 | 3.169 | 1.229 - 5.108 |
| Score [IL-6 tertile = Male_Top] | 22 | 3.608 | 1.64 - 5.576 |
| Score [IL-6 tertile = Female_Bottom] | 22 | 4.558 | 2.648 - 6.468 |
| Score [IL-6 tertile = Female_Middle] | 22 | 4.712 | 2.78 - 6.644 |
| Score [IL-6 tertile = Female_Top] | 22 | 5.009 | 3.045 - 6.972 |
| Score [IL-6 tertile = Male_Bottom] | 25 | 3.173 | 1.255 - 5.091 |
| Score [IL-6 tertile = Male_Middle] | 25 | 2.735 | 0.779 - 4.691 |
| Score [IL-6 tertile = Male_Top] | 25 | 3.489 | 1.502 - 5.476 |
| Score [IL-6 tertile = Female_Bottom] | 25 | 4.333 | 2.413 - 6.252 |
| Score [IL-6 tertile = Female_Middle] | 25 | 4.321 | 2.381 - 6.26 |
| Score [IL-6 tertile = Female_Top] | 25 | 4.608 | 2.638 - 6.578 |
| Score [IL-6 tertile = Male_Bottom] | 28 | 3.371 | 1.425 - 5.317 |
| Score [IL-6 tertile = Male_Middle] | 28 | 2.481 | 0.49 - 4.471 |
| Score [IL-6 tertile = Male_Top] | 28 | 3.212 | 1.187 - 5.237 |
| Score [IL-6 tertile = Female_Bottom] | 28 | 4.894 | 2.954 - 6.835 |
| Score [IL-6 tertile = Female_Middle] | 28 | 4.532 | 2.575 - 6.49 |
| Score [IL-6 tertile = Female_Top] | 28 | 4.993 | 3.007 - 6.98 |

Supplementary Table 15. Model estimates for sensitivity analysis in ALSPAC. IL-6 as a continuous variable.

|  | Unadjusted | | | | Sex Adjusted | | | | Fully Adjusted | | | |
| --- | --- | --- | --- | --- | --- | --- | --- | --- | --- | --- | --- | --- |
| Parameter | Estimate | SE | p-value | N | Estimate | SE | p-value | N | Estimate | SE | p-value | N |
| Intercept | 3.8755 | 0.092 | <0.0001 | 4835 | 3.3908 | 0.102 | <0.0001 | 4835 | 0.8942 | 0.9799 | 0.3615 | 4264 |
| age | <0.0001 | 0.0666 | 0.27 | 4835 | <0.0001 | 0.067 | 0.2949 | 4835 | <0.0001 | 0.0705 | 0.3157 | 4264 |
| age^2 (acceleration) | 0.119 | 0.0152 | <0.0001 | 4835 | 0.1174 | 0.0153 | <0.0001 | 4835 | 0.1176 | 0.016 | <0.0001 | 4264 |
| age^3 (cubic change) | <0.0001 | 0.0012 | <0.0001 | 4835 | <0.0001 | 0.0013 | <0.0001 | 4835 | <0.0001 | 0.0013 | <0.0001 | 4264 |
| age^4 (quartic change) | 0.0003 | <0.0001 | <0.0001 | 4835 | 0.0003 | <0.0001 | <0.0001 | 4835 | 0.0003 | <0.0001 | <0.0001 | 4264 |
| IL6_INT | 0.0305 | 0.0915 | 0.739 | 4835 | <0.0001 | 0.09 | 0.6564 | 4835 | <0.0001 | 0.0954 | 0.3458 | 4264 |
| age:IL6_INT | 0.104 | 0.0661 | 0.1156 | 4835 | 0.1044 | 0.0665 | 0.1164 | 4835 | 0.1168 | 0.0699 | 0.0949 | 4264 |
| age^2 (acceleration):IL6_INT | <0.0001 | 0.0151 | 0.4097 | 4835 | <0.0001 | 0.0152 | 0.4149 | 4835 | <0.0001 | 0.0159 | 0.3616 | 4264 |
| age^3 (cubic change):IL6_INT | 0.0005 | 0.0012 | 0.6845 | 4835 | 0.0005 | 0.0012 | 0.6987 | 4835 | 0.0006 | 0.0013 | 0.6549 | 4264 |
| age^4 (quartic change):IL6_INT | <0.0001 | <0.0001 | 0.861 | 4835 | <0.0001 | <0.0001 | 0.8846 | 4835 | <0.0001 | <0.0001 | 0.8635 | 4264 |
| Sex1 | - | - | - | - | 0.9557 | 0.0947 | <0.0001 | 4835 | 0.9104 | 0.0968 | <0.0001 | 4264 |
| Maternal.education.at.birth1 | - | - | - | - | - | - | - | - | <0.0001 | 0.0958 | 0.1736 | 4264 |
| BMI_age9_log | - | - | - | - | - | - | - | - | 0.8937 | 0.3413 | 0.0088 | 4264 |
| Intercept variance | 9.9354 | 3.152 | - | - | 8.3024 | 2.8814 | - | - | 8.3085 | 2.8824 | - | - |
| Age (slope) variance | 0.4873 | 0.6981 | - | - | 0.6004 | 0.7748 | - | - | 0.6573 | 0.8107 | - | - |
| Quadratic variance | 0.0011 | 0.033 | - | - | 0.0014 | 0.0371 | - | - | 0.0015 | 0.0391 | - | - |
| Intercept/age covariance | -0.8446 | -0.3838 | - | - | -1.0035 | -0.4495 | - | - | -1.2326 | -0.5274 | - | - |
| Intercept/Quadratic covariance | 0.0281 | 0.2698 | - | - | 0.0387 | 0.3626 | - | - | 0.051 | 0.4533 | - | - |
| Age (slope)/Quadratic covariance | -0.0211 | -0.9164 | - | - | -0.0269 | -0.9353 | - | - | -0.0298 | -0.942 | - | - |
| Residual variance | 12.5236 | 3.5389 | - | - | 12.6092 | 3.5509 | - | - | 12.5308 | 3.5399 | - | - |
| Deviance | 170650 |  |  |  | 170392 |  |  |  | 153037 |  |  |  |
| AIC | 170684 |  |  |  | 170428 |  |  |  | 153077 |  |  |  |
| BIC | 170825 |  |  |  | 170578 |  |  |  | 153241 |  |  |  |

Supplementary Table 16. Model estimates for sensitivity analysis in ALSPAC. Individuals taking medication removed.

|  | Unadjusted | | | | Sex Adjusted | | | | Fully Adjusted | | | |
| --- | --- | --- | --- | --- | --- | --- | --- | --- | --- | --- | --- | --- |
| Parameter | Estimate | SE | p-value | N | Estimate | SE | p-value | N | Estimate | SE | p-value | N |
| Intercept | 3.7644 | 0.1688 | <0.0001 | 4140 | 3.3313 | 0.1736 | <0.0001 | 4140 | 1.4316 | 1.0458 | 0.1711 | 3655 |
| age | <0.0001 | 0.1217 | 0.1741 | 4140 | <0.0001 | 0.1224 | 0.185 | 4140 | <0.0001 | 0.128 | 0.1586 | 3655 |
| age^2 (acceleration) | 0.1305 | 0.0278 | <0.0001 | 4140 | 0.1285 | 0.028 | <0.0001 | 4140 | 0.1333 | 0.0292 | <0.0001 | 3655 |
| age^3 (cubic change) | <0.0001 | 0.0023 | <0.0001 | 4140 | <0.0001 | 0.0023 | <0.0001 | 4140 | <0.0001 | 0.0024 | <0.0001 | 3655 |
| age^4 (quartic change) | 0.0003 | <0.0001 | <0.0001 | 4140 | 0.0003 | <0.0001 | <0.0001 | 4140 | 0.0004 | <0.0001 | <0.0001 | 3655 |
| IL6_tertileMiddle | 0.1011 | 0.2421 | 0.6764 | 4140 | 0.0161 | 0.2414 | 0.9468 | 4140 | <0.0001 | 0.2417 | 0.911 | 3655 |
| IL6_tertileTop | 0.118 | 0.2436 | 0.6282 | 4140 | <0.0001 | 0.2434 | 0.7902 | 4140 | <0.0001 | 0.2462 | 0.6394 | 3655 |
| age:IL6_tertileMiddle | 0.0406 | 0.1753 | 0.8167 | 4140 | 0.0389 | 0.1763 | 0.8252 | 4140 | 0.0501 | 0.1847 | 0.786 | 3655 |
| age:IL6_tertileTop | 0.3143 | 0.1759 | 0.0741 | 4140 | 0.3172 | 0.177 | 0.0731 | 4140 | 0.3045 | 0.1865 | 0.1025 | 3655 |
| age^2 (acceleration):IL6_tertileMiddle | <0.0001 | 0.0399 | 0.9568 | 4140 | <0.0001 | 0.0402 | 0.9642 | 4140 | <0.0001 | 0.042 | 0.9646 | 3655 |
| age^2 (acceleration):IL6_tertileTop | <0.0001 | 0.0401 | 0.2644 | 4140 | <0.0001 | 0.0403 | 0.2618 | 4140 | <0.0001 | 0.0425 | 0.3017 | 3655 |
| age^3 (cubic change):IL6_tertileMiddle | <0.0001 | 0.0033 | 0.9965 | 4140 | <0.0001 | 0.0033 | 0.9995 | 4140 | <0.0001 | 0.0034 | 0.9997 | 3655 |
| age^3 (cubic change):IL6_tertileTop | 0.0028 | 0.0033 | 0.3991 | 4140 | 0.0028 | 0.0033 | 0.3951 | 4140 | 0.0027 | 0.0035 | 0.4341 | 3655 |
| age^4 (quartic change):IL6_tertileMiddle | <0.0001 | <0.0001 | 0.9464 | 4140 | <0.0001 | <0.0001 | 0.9479 | 4140 | <0.0001 | <0.0001 | 0.9407 | 3655 |
| age^4 (quartic change):IL6_tertileTop | <0.0001 | <0.0001 | 0.4467 | 4140 | <0.0001 | <0.0001 | 0.4423 | 4140 | <0.0001 | <0.0001 | 0.4768 | 3655 |
| Sex1 | - | - | - | - | 1.0349 | 0.1037 | <0.0001 | 4140 | 0.855 | 0.1023 | <0.0001 | 3655 |
| Maternal.education.at.birth1 | - | - | - | - | - | - | - | - | <0.0001 | 0.1015 | 0.2835 | 3655 |
| BMI_age9_log | - | - | - | - | - | - | - | - | 0.7166 | 0.3672 | 0.051 | 3655 |
| Intercept variance | 10.044 | 3.1693 | - | - | 9.3044 | 3.0503 | - | - | 5.7189 | 2.3914 | - | - |
| Age (slope) variance | 0.5537 | 0.7441 | - | - | 0.5386 | 0.7339 | - | - | 0.6516 | 0.8072 | - | - |
| Quadratic variance | 0.0013 | 0.0355 | - | - | 0.0012 | 0.0346 | - | - | 0.0016 | 0.0399 | - | - |
| Intercept/age covariance | -0.9783 | -0.4148 | - | - | -1.0625 | -0.4746 | - | - | -0.7659 | -0.3968 | - | - |
| Intercept/Quadratic covariance | 0.0349 | 0.31 | - | - | 0.0403 | 0.3825 | - | - | 0.0306 | 0.3214 | - | - |
| Age (slope)/Quadratic covariance | -0.0245 | -0.9283 | - | - | -0.0236 | -0.9284 | - | - | -0.0304 | -0.945 | - | - |
| Residual variance | 12.519 | 3.5382 | - | - | 12.731 | 3.5681 | - | - | 12.647 | 3.5563 | - | - |
| Deviance | 146234 |  |  |  | 146084 |  |  |  | 131018 |  |  |  |
| AIC | 146278 |  |  |  | 146130 |  |  |  | 131068 |  |  |  |
| BIC | 146457 |  |  |  | 146317 |  |  |  | 131269 |  |  |  |

Supplementary Table 17. Model estimates for sensitivity analysis in ALSPAC. Townsend quintiles used instead of maternal education.

|  | Fully Adjusted | | | | Townsend Adjusted | | | |
| --- | --- | --- | --- | --- | --- | --- | --- | --- |
| Parameter | Estimate | SE | p-value | N | Estimate | SE | p-value | N |
| Intercept | 1.1192 | 0.9868 | 0.2567 | 4264 | 1.5355 | 1.0774 | 0.1541 | 3572 |
| age | <0.0001 | 0.12 | 0.0807 | 4264 | <0.0001 | 0.1294 | 0.2122 | 3572 |
| age^2 (acceleration) | 0.1355 | 0.0274 | <0.0001 | 4264 | 0.1265 | 0.0296 | <0.0001 | 3572 |
| age^3 (cubic change) | <0.0001 | 0.0022 | <0.0001 | 4264 | <0.0001 | 0.0024 | <0.0001 | 3572 |
| age^4 (quartic change) | 0.0003 | <0.0001 | <0.0001 | 4264 | 0.0003 | <0.0001 | <0.0001 | 3572 |
| Sex1 | 0.9241 | 0.0976 | <0.0001 | 4264 | 0.923 | 0.1062 | <0.0001 | 3572 |
| Maternal.education.at.birth1 | <0.0001 | 0.0967 | 0.162 | 4264 | - | - | - | - |
| BMI_age9_log | 0.8621 | 0.3458 | 0.0127 | 4264 | 0.6535 | 0.3781 | 0.0839 | 3572 |
| IL6_tertileMiddle | <0.0001 | 0.2356 | 0.5427 | 4264 | <0.0001 | 0.2521 | 0.9537 | 3572 |
| IL6_tertileTop | <0.0001 | 0.238 | 0.2522 | 4264 | <0.0001 | 0.2529 | 0.4034 | 3572 |
| age:IL6_tertileMiddle | 0.0555 | 0.1716 | 0.7462 | 4264 | <0.0001 | 0.1857 | 0.7028 | 3572 |
| age:IL6_tertileTop | 0.3581 | 0.1717 | 0.037 | 4264 | 0.3264 | 0.1845 | 0.0769 | 3572 |
| age^2 (acceleration):IL6_tertileMiddle | <0.0001 | 0.039 | 0.9865 | 4264 | 0.0236 | 0.0423 | 0.5764 | 3572 |
| age^2 (acceleration):IL6_tertileTop | <0.0001 | 0.039 | 0.1821 | 4264 | <0.0001 | 0.042 | 0.2687 | 3572 |
| age^3 (cubic change):IL6_tertileMiddle | <0.0001 | 0.0032 | 0.9293 | 4264 | <0.0001 | 0.0035 | 0.5182 | 3572 |
| age^3 (cubic change):IL6_tertileTop | 0.003 | 0.0032 | 0.3419 | 4264 | 0.0028 | 0.0035 | 0.4157 | 3572 |
| age^4 (quartic change):IL6_tertileMiddle | <0.0001 | <0.0001 | 0.9589 | 4264 | <0.0001 | <0.0001 | 0.5348 | 3572 |
| age^4 (quartic change):IL6_tertileTop | <0.0001 | <0.0001 | 0.4344 | 4264 | <0.0001 | <0.0001 | 0.4842 | 3572 |
| kqTownsendq52 | - | - | - | - | 0.0967 | 0.1539 | 0.5298 | 3572 |
| kqTownsendq53 | - | - | - | - | <0.0001 | 0.1481 | 0.9676 | 3572 |
| kqTownsendq54 | - | - | - | - | 0.1005 | 0.1454 | 0.4895 | 3572 |
| kqTownsendq55 | - | - | - | - | 0.3689 | 0.2157 | 0.0873 | 3572 |
| Intercept variance | 9.3427 | 3.0566 | - | - | 8.0696 | 2.8407 | - | - |
| Age (slope) variance | 0.7297 | 0.8542 | - | - | 0.5791 | 0.761 | - | - |
| Quadratic variance | 0.0017 | 0.0412 | - | - | 0.0013 | 0.036 | - | - |
| Intercept/age covariance | -1.4896 | -0.5705 | - | - | -1.0435 | -0.4827 | - | - |
| Intercept/Quadratic covariance | 0.0633 | 0.5025 | - | - | 0.0403 | 0.394 | - | - |
| Age (slope)/Quadratic covariance | -0.0333 | -0.9469 | - | - | -0.0256 | -0.9336 | - | - |
| Residual variance | 12.3976 | 3.521 | - | - | 12.5691 | 3.5453 | - | - |
| Deviance | 153089 |  |  |  | 131800 |  |  |  |
| AIC | 153139 |  |  |  | 131856 |  |  |  |
| BIC | 153344 |  |  |  | 132081 |  |  |  |

Supplementary Table 18. Model estimates from linear regression of number of SMFQ questionnaires participants completed on IL-6 tertile in ALSPAC. Total number of time points were 11.

| term | estimate | std.error | statistic | p.value | N |
| --- | --- | --- | --- | --- | --- |
| (Intercept) | 6.139 | 0.079 | 77.717 | p<0.0001 | 4835 |
| IL6_tertileMiddle | -0.076 | 0.112 | -0.675 | 0.5 | 4835 |
| IL6_tertileTop | 0.007 | 0.112 | 0.06 | 0.952 | 4835 |

Supplementary Table 19. Model estimates for sensitivity analysis in ALSPAC. Individuals who attended only one assessment removed.

|  | Unadjusted | | | | Sex Adjusted | | | | Fully Adjusted | | | |
| --- | --- | --- | --- | --- | --- | --- | --- | --- | --- | --- | --- | --- |
| Parameter | Estimate | SE | p-value | N | Estimate | SE | p-value | N | Estimate | SE | p-value | N |
| Intercept | 3.8343 | 0.1622 | <0.0001 | 4497 | 3.4638 | 0.1536 | <0.0001 | 4497 | 0.9166 | 0.8709 | 0.2926 | 3997 |
| age | <0.0001 | 0.116 | 0.1382 | 4497 | <0.0001 | 0.1177 | 0.1569 | 4497 | <0.0001 | 0.1248 | 0.1473 | 3997 |
| age^2 (acceleration) | 0.1298 | 0.0264 | <0.0001 | 4497 | 0.1278 | 0.027 | <0.0001 | 4497 | 0.13 | 0.028 | <0.0001 | 3997 |
| age^3 (cubic change) | <0.0001 | 0.0022 | <0.0001 | 4497 | <0.0001 | 0.0022 | <0.0001 | 4497 | <0.0001 | 0.0023 | <0.0001 | 3997 |
| age^4 (quartic change) | 0.0003 | <0.0001 | <0.0001 | 4497 | 0.0003 | <0.0001 | <0.0001 | 4497 | 0.0003 | <0.0001 | <0.0001 | 3997 |
| IL6_tertileMiddle | 0.0495 | 0.2311 | 0.8303 | 4497 | <0.0001 | 0.2126 | 0.8914 | 4497 | <0.0001 | 0.2433 | 0.6718 | 3997 |
| IL6_tertileTop | 0.0002 | 0.2307 | 0.9993 | 4497 | <0.0001 | 0.2126 | 0.5027 | 4497 | <0.0001 | 0.2454 | 0.2624 | 3997 |
| age:IL6_tertileMiddle | 0.0059 | 0.1657 | 0.9717 | 4497 | 0.0151 | 0.1681 | 0.9285 | 4497 | 0.0209 | 0.1784 | 0.9066 | 3997 |
| age:IL6_tertileTop | 0.3151 | 0.1649 | 0.0561 | 4497 | 0.3126 | 0.1674 | 0.0618 | 4497 | 0.354 | 0.1786 | 0.0475 | 3997 |
| age^2 (acceleration):IL6_tertileMiddle | 0.0071 | 0.0376 | 0.8492 | 4497 | 0.0048 | 0.0384 | 0.9002 | 4497 | 0.007 | 0.0399 | 0.86 | 3997 |
| age^2 (acceleration):IL6_tertileTop | <0.0001 | 0.0374 | 0.2579 | 4497 | <0.0001 | 0.0382 | 0.2726 | 4497 | <0.0001 | 0.0399 | 0.2079 | 3997 |
| age^3 (cubic change):IL6_tertileMiddle | <0.0001 | 0.0031 | 0.7785 | 4497 | <0.0001 | 0.0032 | 0.823 | 4497 | <0.0001 | 0.0033 | 0.7819 | 3997 |
| age^3 (cubic change):IL6_tertileTop | 0.0023 | 0.0031 | 0.452 | 4497 | 0.0023 | 0.0031 | 0.4671 | 4497 | 0.0028 | 0.0033 | 0.3872 | 3997 |
| age^4 (quartic change):IL6_tertileMiddle | <0.0001 | <0.0001 | 0.8079 | 4497 | <0.0001 | <0.0001 | 0.8418 | 4497 | <0.0001 | <0.0001 | 0.8142 | 3997 |
| age^4 (quartic change):IL6_tertileTop | <0.0001 | <0.0001 | 0.554 | 4497 | <0.0001 | <0.0001 | 0.5703 | 4497 | <0.0001 | <0.0001 | 0.4946 | 3997 |
| Sex1 | - | - | - | - | 0.8563 | 0.0883 | <0.0001 | 4497 | 0.893 | 0.0853 | <0.0001 | 3997 |
| Maternal.education.at.birth1 | - | - | - | - | - | - | - | - | <0.0001 | 0.0845 | 0.2593 | 3997 |
| BMI_age9_log | - | - | - | - | - | - | - | - | 0.913 | 0.3036 | 0.0026 | 3997 |
| Intercept variance | 9.888 | 3.1445 | - | - | 2.5053 | 1.5828 | - | - | 9.5027 | 3.0826 | - | - |
| Age (slope) variance | 0.7068 | 0.8407 | - | - | 0.385 | 0.6205 | - | - | 1.5029 | 1.2259 | - | - |
| Quadratic variance | 0.0016 | 0.0402 | - | - | 0.0009 | 0.0307 | - | - | 0.0037 | 0.0606 | - | - |
| Intercept/age covariance | -1.4127 | -0.5344 | - | - | 0.0627 | 0.0638 | - | - | -3.0718 | -0.8128 | - | - |
| Intercept/Quadratic covariance | 0.0588 | 0.4649 | - | - | -0.0077 | -0.1592 | - | - | 0.1502 | 0.8035 | - | - |
| Age (slope)/Quadratic covariance | -0.0319 | -0.9443 | - | - | -0.0173 | -0.9079 | - | - | -0.0727 | -0.9775 | - | - |
| Residual variance | 12.5511 | 3.5427 | - | - | 13.4127 | 3.6623 | - | - | 12.7094 | 3.565 | - | - |
| Deviance | 168709 |  |  |  | 168280 |  |  |  | 152011 |  |  |  |
| AIC | 168753 |  |  |  | 168326 |  |  |  | 152061 |  |  |  |
| BIC | 168935 |  |  |  | 168517 |  |  |  | 152265 |  |  |  |

Supplementary Table 20. Estimated depression scores for each IL-6 tertile trajectory at ages 40, 50, 60, 70 and 80 years, in UK Biobank.

| IL-6 Tertile Group | age | estimate | 95% CI |
| --- | --- | --- | --- |
| Score [IL-6 tertile = Bottom] | 40 | 0.833 | 0.571 - 1.094 |
| Score [IL-6 tertile = Middle] | 40 | 0.963 | 0.699 - 1.226 |
| Score [IL-6 tertile = Top] | 40 | 1.02 | 0.754 - 1.286 |
| Score [IL-6 tertile = Bottom] | 50 | 0.771 | 0.512 - 1.029 |
| Score [IL-6 tertile = Middle] | 50 | 0.807 | 0.548 - 1.066 |
| Score [IL-6 tertile = Top] | 50 | 0.83 | 0.571 - 1.089 |
| Score [IL-6 tertile = Bottom] | 60 | 0.725 | 0.467 - 0.983 |
| Score [IL-6 tertile = Middle] | 60 | 0.713 | 0.455 - 0.971 |
| Score [IL-6 tertile = Top] | 60 | 0.725 | 0.466 - 0.983 |
| Score [IL-6 tertile = Bottom] | 70 | 0.696 | 0.437 - 0.954 |
| Score [IL-6 tertile = Middle] | 70 | 0.681 | 0.423 - 0.94 |
| Score [IL-6 tertile = Top] | 70 | 0.704 | 0.446 - 0.963 |
| Score [IL-6 tertile = Bottom] | 80 | 0.683 | 0.422 - 0.944 |
| Score [IL-6 tertile = Middle] | 80 | 0.712 | 0.451 - 0.973 |
| Score [IL-6 tertile = Top] | 80 | 0.769 | 0.508 - 1.03 |

Supplementary Table 21. Model estimates for main analysis in UK Biobank. IL-6 as a categorical variable (tertiles).

|  | Unadjusted | | | | Sex Adjusted | | | | Fully Adjusted | | | |
| --- | --- | --- | --- | --- | --- | --- | --- | --- | --- | --- | --- | --- |
| Parameter | Estimate | SE | p-value | N | Estimate | SE | p-value | N | Estimate | SE | p-value | N |
| Intercept | 0.7568 | 0.1344 | <0.0001 | 39613 | 0.7054 | 0.1344 | <0.0001 | 39613 | 0.8401 | 0.1338 | <0.0001 | 39285 |
| age | <0.0001 | 0.0022 | 0.0002 | 39613 | <0.0001 | 0.0022 | 0.0001 | 39613 | <0.0001 | 0.0022 | 0.0011 | 39285 |
| age^2 (acceleration) | <0.0001 | <0.0001 | 0.097 | 39613 | <0.0001 | <0.0001 | 0.0883 | 39613 | <0.0001 | <0.0001 | 0.096 | 39285 |
| Batch1 | 0.0243 | 0.1323 | 0.8541 | 39613 | 0.0258 | 0.1322 | 0.8452 | 39613 | <0.0001 | 0.1303 | 0.9231 | 39285 |
| Batch2 | 0.0131 | 0.1305 | 0.92 | 39613 | 0.0135 | 0.1304 | 0.9175 | 39613 | <0.0001 | 0.1285 | 0.8528 | 39285 |
| Batch3 | 0.0419 | 0.1301 | 0.7474 | 39613 | 0.0425 | 0.1299 | 0.7438 | 39613 | 0.0071 | 0.1281 | 0.9556 | 39285 |
| Batch4 | 0.0676 | 0.1303 | 0.6039 | 39613 | 0.068 | 0.1301 | 0.6011 | 39613 | 0.0306 | 0.1283 | 0.8116 | 39285 |
| Batch5 | 0.0298 | 0.1303 | 0.8189 | 39613 | 0.031 | 0.1301 | 0.8119 | 39613 | <0.0001 | 0.1283 | 0.9394 | 39285 |
| Batch6 | 0.0367 | 0.1303 | 0.7782 | 39613 | 0.0374 | 0.1301 | 0.7737 | 39613 | 0.0033 | 0.1283 | 0.9795 | 39285 |
| Batch7 | 0.0257 | 0.1305 | 0.8438 | 39613 | 0.0262 | 0.1304 | 0.8408 | 39613 | <0.0001 | 0.1285 | 0.9203 | 39285 |
| Assessment_centre_baselineBirmingham | <0.0001 | 0.0344 | 0.3281 | 39613 | <0.0001 | 0.0344 | 0.3891 | 39613 | 0.1492 | 0.0352 | <0.0001 | 39285 |
| Assessment_centre_baselineBristol | <0.0001 | 0.0316 | <0.0001 | 39613 | <0.0001 | 0.0316 | <0.0001 | 39613 | 0.064 | 0.0332 | 0.0541 | 39285 |
| Assessment_centre_baselineBury | <0.0001 | 0.033 | <0.0001 | 39613 | <0.0001 | 0.0329 | <0.0001 | 39613 | 0.0439 | 0.0343 | 0.2011 | 39285 |
| Assessment_centre_baselineCardiff | <0.0001 | 0.0361 | 0.0446 | 39613 | <0.0001 | 0.0361 | 0.0536 | 39613 | 0.1604 | 0.0374 | <0.0001 | 39285 |
| Assessment_centre_baselineCroydon | <0.0001 | 0.0336 | 0.0762 | 39613 | <0.0001 | 0.0336 | 0.0772 | 39613 | 0.1229 | 0.0342 | 0.0003 | 39285 |
| Assessment_centre_baselineEdinburgh | <0.0001 | 0.0369 | <0.0001 | 39613 | <0.0001 | 0.0369 | <0.0001 | 39613 | 0.0332 | 0.038 | 0.3822 | 39285 |
| Assessment_centre_baselineGlasgow | <0.0001 | 0.0362 | 0.0031 | 39613 | <0.0001 | 0.0361 | 0.0037 | 39613 | 0.0652 | 0.0367 | 0.0758 | 39285 |
| Assessment_centre_baselineHounslow | <0.0001 | 0.0332 | 0.0002 | 39613 | <0.0001 | 0.0331 | 0.0002 | 39613 | 0.0549 | 0.0337 | 0.1035 | 39285 |
| Assessment_centre_baselineLeeds | <0.0001 | 0.0307 | <0.0001 | 39613 | <0.0001 | 0.0307 | <0.0001 | 39613 | 0.0468 | 0.0322 | 0.1459 | 39285 |
| Assessment_centre_baselineLiverpool | <0.0001 | 0.0333 | 0.0002 | 39613 | <0.0001 | 0.0333 | 0.0003 | 39613 | 0.0892 | 0.0344 | 0.0095 | 39285 |
| Assessment_centre_baselineManchester | <0.0001 | 0.0379 | 0.2771 | 39613 | <0.0001 | 0.0379 | 0.3276 | 39613 | 0.1497 | 0.0385 | 0.0001 | 39285 |
| Assessment_centre_baselineMiddlesborough | <0.0001 | 0.0346 | <0.0001 | 39613 | <0.0001 | 0.0346 | <0.0001 | 39613 | 0.0843 | 0.0358 | 0.0185 | 39285 |
| Assessment_centre_baselineNewcastle | <0.0001 | 0.0323 | <0.0001 | 39613 | <0.0001 | 0.0323 | <0.0001 | 39613 | 0.0529 | 0.0333 | 0.1123 | 39285 |
| Assessment_centre_baselineNottingham | <0.0001 | 0.0315 | <0.0001 | 39613 | <0.0001 | 0.0315 | <0.0001 | 39613 | 0.1048 | 0.033 | 0.0015 | 39285 |
| Assessment_centre_baselineOxford | <0.0001 | 0.0395 | <0.0001 | 39613 | <0.0001 | 0.0395 | <0.0001 | 39613 | 0.0548 | 0.0405 | 0.1763 | 39285 |
| Assessment_centre_baselineReading | <0.0001 | 0.0338 | <0.0001 | 39613 | <0.0001 | 0.0338 | <0.0001 | 39613 | 0.0665 | 0.0357 | 0.0625 | 39285 |
| Assessment_centre_baselineSheffield | <0.0001 | 0.0327 | 0.0001 | 39613 | <0.0001 | 0.0327 | 0.0002 | 39613 | 0.1025 | 0.0339 | 0.0025 | 39285 |
| Assessment_centre_baselineStoke | <0.0001 | 0.0363 | 0.0005 | 39613 | <0.0001 | 0.0363 | 0.0012 | 39613 | 0.1303 | 0.0377 | 0.0005 | 39285 |
| Assessment_centre_baselineSwansea | <0.0001 | 0.0933 | 0.7119 | 39613 | <0.0001 | 0.0932 | 0.7373 | 39613 | 0.1484 | 0.093 | 0.1105 | 39285 |
| Assessment_centre_baselineWrexham | <0.0001 | 0.16 | 0.9902 | 39613 | <0.0001 | 0.1599 | 0.9988 | 39613 | 0.2072 | 0.158 | 0.1897 | 39285 |
| IL6_tertileMiddle | 0.2083 | 0.0401 | <0.0001 | 39613 | 0.2097 | 0.0402 | <0.0001 | 39613 | 0.1417 | 0.0399 | 0.0004 | 39285 |
| IL6_tertileTop | 0.3339 | 0.0452 | <0.0001 | 39613 | 0.3317 | 0.0454 | <0.0001 | 39613 | 0.2041 | 0.0452 | <0.0001 | 39285 |
| age:IL6_tertileMiddle | <0.0001 | 0.0034 | <0.0001 | 39613 | <0.0001 | 0.0034 | <0.0001 | 39613 | <0.0001 | 0.0034 | 0.0003 | 39285 |
| age:IL6_tertileTop | <0.0001 | 0.0038 | <0.0001 | 39613 | <0.0001 | 0.0038 | <0.0001 | 39613 | <0.0001 | 0.0037 | <0.0001 | 39285 |
| age^2 (acceleration):IL6_tertileMiddle | 0.0002 | <0.0001 | 0.0007 | 39613 | 0.0002 | <0.0001 | 0.0007 | 39613 | 0.0002 | <0.0001 | 0.0015 | 39285 |
| age^2 (acceleration):IL6_tertileTop | 0.0003 | <0.0001 | <0.0001 | 39613 | 0.0003 | <0.0001 | <0.0001 | 39613 | 0.0003 | <0.0001 | <0.0001 | 39285 |
| SexFemale | - | - | - | - | 0.0908 | 0.0096 | <0.0001 | 39613 | 0.1218 | 0.0096 | <0.0001 | 39285 |
| smoking_statusNever | - | - | - | - | - | - | - | - | <0.0001 | 0.0169 | <0.0001 | 39285 |
| smoking_statusPrevious | - | - | - | - | - | - | - | - | <0.0001 | 0.0175 | <0.0001 | 39285 |
| Townsend | - | - | - | - | - | - | - | - | 0.1144 | 0.0054 | <0.0001 | 39285 |
| BMI | - | - | - | - | - | - | - | - | 0.072 | 0.0052 | <0.0001 | 39285 |
| Intercept variance | 1.2788 | 1.1308 | - | - | 1.3097 | 1.1444 | - | - | 1.2126 | 1.1012 | - | - |
| Age (slope) variance | 0.0011 | 0.0333 | - | - | 0.0011 | 0.0338 | - | - | 0.0011 | 0.0328 | - | - |
| Intercept/age covariance | -0.0292 | -0.776 | - | - | -0.0303 | -0.7838 | - | - | -0.0279 | -0.7736 | - | - |
| Residual variance | 0.5893 | 0.7677 | - | - | 0.5877 | 0.7666 | - | - | 0.5885 | 0.7671 | - | - |
| Deviance | 283287 |  |  |  | 283198 |  |  |  | 280150 |  |  |  |
| AIC | 283367 |  |  |  | 283280 |  |  |  | 280240 |  |  |  |
| BIC | 283748 |  |  |  | 283671 |  |  |  | 280668 |  |  |  |

Supplementary Table 22. Model estimates for main analysis in UK Biobank split by sex. A variable was created that split the IL-6 tertiles by sex: female & bottom third IL-6 tertile, female & middle third IL-6 tertile, female & top third IL-6 tertile, male & bottom third IL-6 tertile, male & middle third IL-6 tertile and male & top third IL-6 tertile. The models were then run splitting the trajectories on this sex-split IL-6 tertile variable.

|  | Unadjusted | | | | Fully Adjusted | | | |
| --- | --- | --- | --- | --- | --- | --- | --- | --- |
| Parameter | Estimate | SE | p-value | N | Estimate | SE | p-value | N |
| Intercept | 0.8032 | 0.0453 | <0.0001 | 39613 | 0.8849 | 0.05 | <0.0001 | 39285 |
| age | <0.0001 | 0.0032 | <0.0001 | 39613 | <0.0001 | 0.0033 | 0.0015 | 39285 |
| age^2 (acceleration) | 0.0001 | <0.0001 | 0.0641 | 39613 | 0.0001 | <0.0001 | 0.0981 | 39285 |
| Batch | 0.0001 | 0.0028 | 0.964 | 39613 | <0.0001 | 0.0028 | 0.9649 | 39285 |
| Assessment_centre_baselineBirmingham | <0.0001 | 0.034 | 0.4015 | 39613 | 0.1493 | 0.0352 | <0.0001 | 39285 |
| Assessment_centre_baselineBristol | <0.0001 | 0.0312 | <0.0001 | 39613 | 0.064 | 0.0332 | 0.0538 | 39285 |
| Assessment_centre_baselineBury | <0.0001 | 0.0326 | <0.0001 | 39613 | 0.0431 | 0.0343 | 0.2096 | 39285 |
| Assessment_centre_baselineCardiff | <0.0001 | 0.0357 | 0.0519 | 39613 | 0.1612 | 0.0374 | <0.0001 | 39285 |
| Assessment_centre_baselineCroydon | <0.0001 | 0.0333 | 0.0846 | 39613 | 0.1246 | 0.0342 | 0.0003 | 39285 |
| Assessment_centre_baselineEdinburgh | <0.0001 | 0.0365 | <0.0001 | 39613 | 0.0337 | 0.038 | 0.3755 | 39285 |
| Assessment_centre_baselineGlasgow | <0.0001 | 0.0358 | 0.003 | 39613 | 0.0652 | 0.0367 | 0.0759 | 39285 |
| Assessment_centre_baselineHounslow | <0.0001 | 0.0328 | 0.0004 | 39613 | 0.0557 | 0.0337 | 0.0984 | 39285 |
| Assessment_centre_baselineLeeds | <0.0001 | 0.0304 | <0.0001 | 39613 | 0.047 | 0.0322 | 0.1445 | 39285 |
| Assessment_centre_baselineLiverpool | <0.0001 | 0.033 | 0.0003 | 39613 | 0.0892 | 0.0344 | 0.0095 | 39285 |
| Assessment_centre_baselineManchester | <0.0001 | 0.0375 | 0.3308 | 39613 | 0.1504 | 0.0385 | <0.0001 | 39285 |
| Assessment_centre_baselineMiddlesborough | <0.0001 | 0.0343 | <0.0001 | 39613 | 0.085 | 0.0358 | 0.0176 | 39285 |
| Assessment_centre_baselineNewcastle | <0.0001 | 0.0319 | <0.0001 | 39613 | 0.0525 | 0.0333 | 0.1147 | 39285 |
| Assessment_centre_baselineNottingham | <0.0001 | 0.0312 | <0.0001 | 39613 | 0.1054 | 0.033 | 0.0014 | 39285 |
| Assessment_centre_baselineOxford | <0.0001 | 0.0391 | <0.0001 | 39613 | 0.0557 | 0.0405 | 0.169 | 39285 |
| Assessment_centre_baselineReading | <0.0001 | 0.0335 | <0.0001 | 39613 | 0.0664 | 0.0357 | 0.063 | 39285 |
| Assessment_centre_baselineSheffield | <0.0001 | 0.0323 | 0.0002 | 39613 | 0.1023 | 0.0339 | 0.0025 | 39285 |
| Assessment_centre_baselineStoke | <0.0001 | 0.036 | 0.001 | 39613 | 0.1308 | 0.0377 | 0.0005 | 39285 |
| Assessment_centre_baselineSwansea | <0.0001 | 0.0926 | 0.8299 | 39613 | 0.1549 | 0.093 | 0.0958 | 39285 |
| Assessment_centre_baselineWrexham | <0.0001 | 0.1576 | 0.9098 | 39613 | 0.2 | 0.158 | 0.2055 | 39285 |
| IL6_sex_tertileMale_Middle | 0.2263 | 0.0561 | <0.0001 | 39613 | 0.1581 | 0.0584 | 0.0068 | 39285 |
| IL6_sex_tertileMale_Top | 0.3484 | 0.0646 | <0.0001 | 39613 | 0.2325 | 0.0674 | 0.0006 | 39285 |
| IL6_sex_tertileFemale_Bottom | <0.0001 | 0.0481 | 0.8933 | 39613 | 0.0402 | 0.0502 | 0.4232 | 39285 |
| IL6_sex_tertileFemale_Middle | 0.1918 | 0.0539 | 0.0004 | 39613 | 0.1687 | 0.0562 | 0.0027 | 39285 |
| IL6_sex_tertileFemale_Top | 0.319 | 0.0596 | <0.0001 | 39613 | 0.2263 | 0.0622 | 0.0003 | 39285 |
| age:IL6_sex_tertileMale_Middle | <0.0001 | 0.0048 | 0.004 | 39613 | <0.0001 | 0.005 | 0.0158 | 39285 |
| age:IL6_sex_tertileMale_Top | <0.0001 | 0.0054 | 0.0007 | 39613 | <0.0001 | 0.0056 | 0.0011 | 39285 |
| age:IL6_sex_tertileFemale_Bottom | 0.006 | 0.0043 | 0.1651 | 39613 | 0.0058 | 0.0044 | 0.1899 | 39285 |
| age:IL6_sex_tertileFemale_Middle | <0.0001 | 0.0046 | 0.1236 | 39613 | <0.0001 | 0.0048 | 0.1714 | 39285 |
| age:IL6_sex_tertileFemale_Top | <0.0001 | 0.0051 | 0.0222 | 39613 | <0.0001 | 0.0052 | 0.0456 | 39285 |
| age^2 (acceleration):IL6_sex_tertileMale_Middle | 0.0002 | 0.0001 | 0.0223 | 39613 | 0.0002 | 0.0001 | 0.0542 | 39285 |
| age^2 (acceleration):IL6_sex_tertileMale_Top | 0.0003 | 0.0001 | 0.0023 | 39613 | 0.0004 | 0.0001 | 0.0018 | 39285 |
| age^2 (acceleration):IL6_sex_tertileFemale_Bottom | <0.0001 | <0.0001 | 0.5309 | 39613 | <0.0001 | <0.0001 | 0.4638 | 39285 |
| age^2 (acceleration):IL6_sex_tertileFemale_Middle | 0.0002 | 0.0001 | 0.0575 | 39613 | 0.0002 | 0.0001 | 0.0802 | 39285 |
| age^2 (acceleration):IL6_sex_tertileFemale_Top | 0.0003 | 0.0001 | 0.0119 | 39613 | 0.0003 | 0.0001 | 0.02 | 39285 |
| smoking_statusNever | - | - | - | - | <0.0001 | 0.0169 | <0.0001 | 39285 |
| smoking_statusPrevious | - | - | - | - | <0.0001 | 0.0175 | <0.0001 | 39285 |
| Townsend | - | - | - | - | 0.1142 | 0.0054 | <0.0001 | 39285 |
| BMI | - | - | - | - | 0.0721 | 0.0052 | <0.0001 | 39285 |
| Intercept variance | 0.9557 | 0.9776 | - | - | 1.2112 | 1.1006 | - | - |
| Age (slope) variance | 0.0007 | 0.026 | - | - | 0.0011 | 0.0327 | - | - |
| Intercept/age covariance | -0.0174 | -0.6849 | - | - | -0.0278 | -0.7731 | - | - |
| Residual variance | 0.6129 | 0.7829 | - | - | 0.5884 | 0.7671 | - | - |
| Deviance | 283310.87 |  |  |  | 280144.41 |  |  |  |
| AIC | 283396.87 |  |  |  | 280238.41 |  |  |  |
| BIC | 283806.47 |  |  |  | 280685.83 |  |  |  |

Supplementary Table 23. Estimated depression scores for each IL-6 tertile trajectory at ages 40, 50, 60, 70 and 80 years, in UK Biobank, split by sex.

| IL-6 Tertile Group | age | estimate | 95% CI |
| --- | --- | --- | --- |
| Score [IL-6 tertile = Male_Bottom] | 40 | 0.875 | 0.781 - 0.969 |
| Score [IL-6 tertile = Male_Middle] | 40 | 1.021 | 0.916 - 1.126 |
| Score [IL-6 tertile = Male_Top] | 40 | 1.089 | 0.968 - 1.211 |
| Score [IL-6 tertile = Female_Bottom] | 40 | 0.921 | 0.83 - 1.011 |
| Score [IL-6 tertile = Female_Middle] | 40 | 1.037 | 0.936 - 1.138 |
| Score [IL-6 tertile = Female_Top] | 40 | 1.091 | 0.979 - 1.203 |
| Score [IL-6 tertile = Male_Bottom] | 50 | 0.785 | 0.712 - 0.859 |
| Score [IL-6 tertile = Male_Middle] | 50 | 0.837 | 0.761 - 0.912 |
| Score [IL-6 tertile = Male_Top] | 50 | 0.862 | 0.783 - 0.941 |
| Score [IL-6 tertile = Female_Bottom] | 50 | 0.881 | 0.808 - 0.953 |
| Score [IL-6 tertile = Female_Middle] | 50 | 0.904 | 0.829 - 0.979 |
| Score [IL-6 tertile = Female_Top] | 50 | 0.928 | 0.851 - 1.005 |
| Score [IL-6 tertile = Male_Bottom] | 60 | 0.72 | 0.649 - 0.792 |
| Score [IL-6 tertile = Male_Middle] | 60 | 0.717 | 0.646 - 0.789 |
| Score [IL-6 tertile = Male_Top] | 60 | 0.732 | 0.66 - 0.803 |
| Score [IL-6 tertile = Female_Bottom] | 60 | 0.85 | 0.78 - 0.921 |
| Score [IL-6 tertile = Female_Middle] | 60 | 0.831 | 0.76 - 0.902 |
| Score [IL-6 tertile = Female_Top] | 60 | 0.842 | 0.77 - 0.913 |
| Score [IL-6 tertile = Male_Bottom] | 70 | 0.68 | 0.607 - 0.753 |
| Score [IL-6 tertile = Male_Middle] | 70 | 0.664 | 0.592 - 0.735 |
| Score [IL-6 tertile = Male_Top] | 70 | 0.699 | 0.627 - 0.771 |
| Score [IL-6 tertile = Female_Bottom] | 70 | 0.83 | 0.758 - 0.902 |
| Score [IL-6 tertile = Female_Middle] | 70 | 0.819 | 0.747 - 0.89 |
| Score [IL-6 tertile = Female_Top] | 70 | 0.831 | 0.759 - 0.903 |
| Score [IL-6 tertile = Male_Bottom] | 80 | 0.663 | 0.572 - 0.755 |
| Score [IL-6 tertile = Male_Middle] | 80 | 0.675 | 0.588 - 0.762 |
| Score [IL-6 tertile = Male_Top] | 80 | 0.764 | 0.673 - 0.854 |
| Score [IL-6 tertile = Female_Bottom] | 80 | 0.819 | 0.732 - 0.907 |
| Score [IL-6 tertile = Female_Middle] | 80 | 0.867 | 0.782 - 0.952 |
| Score [IL-6 tertile = Female_Top] | 80 | 0.897 | 0.808 - 0.986 |

Supplementary Table 24. Model estimates for sensitivity analysis in UK Biobank. IL-6 as a continuous variable.

|  | Unadjusted | | | | Sex Adjusted | | | | Fully Adjusted | | | |
| --- | --- | --- | --- | --- | --- | --- | --- | --- | --- | --- | --- | --- |
| Parameter | Estimate | SE | p-value | N | Estimate | SE | p-value | N | Estimate | SE | p-value | N |
| Intercept | 0.9431 | 0.132 | <0.0001 | 39613 | 0.8894 | 0.1332 | <0.0001 | 39613 | 0.9558 | 0.1323 | <0.0001 | 39285 |
| age | <0.0001 | 0.0015 | <0.0001 | 39613 | <0.0001 | 0.0015 | <0.0001 | 39613 | <0.0001 | 0.0015 | <0.0001 | 39285 |
| age^2 (acceleration) | 0.0003 | <0.0001 | <0.0001 | 39613 | 0.0003 | <0.0001 | <0.0001 | 39613 | 0.0003 | <0.0001 | <0.0001 | 39285 |
| Batch1 | 0.0267 | 0.1312 | 0.8388 | 39613 | 0.0226 | 0.1321 | 0.8639 | 39613 | <0.0001 | 0.13 | 0.926 | 39285 |
| Batch2 | 0.0174 | 0.1294 | 0.8931 | 39613 | 0.0116 | 0.1303 | 0.9292 | 39613 | <0.0001 | 0.1282 | 0.8573 | 39285 |
| Batch3 | 0.0448 | 0.129 | 0.7285 | 39613 | 0.0398 | 0.1299 | 0.7592 | 39613 | 0.0077 | 0.1278 | 0.9521 | 39285 |
| Batch4 | 0.07 | 0.1292 | 0.5877 | 39613 | 0.0655 | 0.1301 | 0.6144 | 39613 | 0.031 | 0.128 | 0.8084 | 39285 |
| Batch5 | 0.0337 | 0.1292 | 0.794 | 39613 | 0.0289 | 0.1301 | 0.8244 | 39613 | <0.0001 | 0.128 | 0.9443 | 39285 |
| Batch6 | 0.0392 | 0.1292 | 0.7612 | 39613 | 0.0345 | 0.1301 | 0.7909 | 39613 | 0.0038 | 0.128 | 0.9764 | 39285 |
| Batch7 | 0.0289 | 0.1294 | 0.823 | 39613 | 0.0239 | 0.1303 | 0.8547 | 39613 | <0.0001 | 0.1282 | 0.9229 | 39285 |
| Assessment_centre_baselineBirmingham | <0.0001 | 0.0341 | 0.3241 | 39613 | <0.0001 | 0.0344 | 0.3833 | 39613 | 0.1499 | 0.035 | <0.0001 | 39285 |
| Assessment_centre_baselineBristol | <0.0001 | 0.0313 | <0.0001 | 39613 | <0.0001 | 0.0316 | <0.0001 | 39613 | 0.0644 | 0.0331 | 0.0519 | 39285 |
| Assessment_centre_baselineBury | <0.0001 | 0.0327 | <0.0001 | 39613 | <0.0001 | 0.0329 | <0.0001 | 39613 | 0.0446 | 0.0342 | 0.1926 | 39285 |
| Assessment_centre_baselineCardiff | <0.0001 | 0.0357 | 0.036 | 39613 | <0.0001 | 0.036 | 0.0482 | 39613 | 0.1605 | 0.0373 | <0.0001 | 39285 |
| Assessment_centre_baselineCroydon | <0.0001 | 0.0333 | 0.0706 | 39613 | <0.0001 | 0.0336 | 0.0747 | 39613 | 0.1233 | 0.0341 | 0.0003 | 39285 |
| Assessment_centre_baselineEdinburgh | <0.0001 | 0.0366 | <0.0001 | 39613 | <0.0001 | 0.0368 | <0.0001 | 39613 | 0.0336 | 0.0379 | 0.3763 | 39285 |
| Assessment_centre_baselineGlasgow | <0.0001 | 0.0358 | 0.0023 | 39613 | <0.0001 | 0.0361 | 0.0034 | 39613 | 0.0652 | 0.0366 | 0.0753 | 39285 |
| Assessment_centre_baselineHounslow | <0.0001 | 0.0328 | 0.0003 | 39613 | <0.0001 | 0.0331 | 0.0003 | 39613 | 0.0566 | 0.0336 | 0.0922 | 39285 |
| Assessment_centre_baselineLeeds | <0.0001 | 0.0304 | <0.0001 | 39613 | <0.0001 | 0.0307 | <0.0001 | 39613 | 0.0476 | 0.0321 | 0.1382 | 39285 |
| Assessment_centre_baselineLiverpool | <0.0001 | 0.033 | 0.0002 | 39613 | <0.0001 | 0.0333 | 0.0003 | 39613 | 0.0903 | 0.0343 | 0.0085 | 39285 |
| Assessment_centre_baselineManchester | <0.0001 | 0.0375 | 0.2725 | 39613 | <0.0001 | 0.0379 | 0.334 | 39613 | 0.1506 | 0.0384 | <0.0001 | 39285 |
| Assessment_centre_baselineMiddlesborough | <0.0001 | 0.0343 | <0.0001 | 39613 | <0.0001 | 0.0346 | <0.0001 | 39613 | 0.0852 | 0.0357 | 0.017 | 39285 |
| Assessment_centre_baselineNewcastle | <0.0001 | 0.032 | <0.0001 | 39613 | <0.0001 | 0.0322 | <0.0001 | 39613 | 0.0533 | 0.0332 | 0.108 | 39285 |
| Assessment_centre_baselineNottingham | <0.0001 | 0.0312 | <0.0001 | 39613 | <0.0001 | 0.0315 | <0.0001 | 39613 | 0.1056 | 0.0329 | 0.0013 | 39285 |
| Assessment_centre_baselineOxford | <0.0001 | 0.0392 | <0.0001 | 39613 | <0.0001 | 0.0395 | <0.0001 | 39613 | 0.0566 | 0.0404 | 0.1618 | 39285 |
| Assessment_centre_baselineReading | <0.0001 | 0.0335 | <0.0001 | 39613 | <0.0001 | 0.0338 | <0.0001 | 39613 | 0.0676 | 0.0356 | 0.0576 | 39285 |
| Assessment_centre_baselineSheffield | <0.0001 | 0.0324 | 0.0001 | 39613 | <0.0001 | 0.0326 | 0.0002 | 39613 | 0.1031 | 0.0338 | 0.0023 | 39285 |
| Assessment_centre_baselineStoke | <0.0001 | 0.036 | 0.0004 | 39613 | <0.0001 | 0.0363 | 0.0012 | 39613 | 0.1308 | 0.0376 | 0.0005 | 39285 |
| Assessment_centre_baselineSwansea | <0.0001 | 0.0927 | 0.7579 | 39613 | <0.0001 | 0.0932 | 0.7531 | 39613 | 0.1507 | 0.0928 | 0.1044 | 39285 |
| Assessment_centre_baselineWrexham | <0.0001 | 0.1577 | 0.956 | 39613 | 0.0009 | 0.1598 | 0.9956 | 39613 | 0.2069 | 0.1574 | 0.1886 | 39285 |
| IL6_INT | 0.1289 | 0.0167 | <0.0001 | 39613 | 0.1286 | 0.0174 | <0.0001 | 39613 | 0.0751 | 0.0172 | <0.0001 | 39285 |
| age:IL6_INT | <0.0001 | 0.0014 | <0.0001 | 39613 | <0.0001 | 0.0015 | <0.0001 | 39613 | <0.0001 | 0.0015 | <0.0001 | 39285 |
| age^2 (acceleration):IL6_INT | 0.0001 | <0.0001 | <0.0001 | 39613 | 0.0001 | <0.0001 | <0.0001 | 39613 | 0.0001 | <0.0001 | <0.0001 | 39285 |
| SexFemale | - | - | - | - | 0.0916 | 0.0096 | <0.0001 | 39613 | 0.1218 | 0.0096 | <0.0001 | 39285 |
| smoking_statusNever | - | - | - | - | - | - | - | - | <0.0001 | 0.0168 | <0.0001 | 39285 |
| smoking_statusPrevious | - | - | - | - | - | - | - | - | <0.0001 | 0.0174 | <0.0001 | 39285 |
| Townsend | - | - | - | - | - | - | - | - | 0.1149 | 0.0054 | <0.0001 | 39285 |
| BMI | - | - | - | - | - | - | - | - | 0.0722 | 0.0052 | <0.0001 | 39285 |
| Intercept variance | 0.9643 | 0.982 | - | - | 1.2791 | 1.131 | - | - | 1.121 | 1.0588 | - | - |
| Age (slope) variance | 0.0007 | 0.0263 | - | - | 0.0011 | 0.0332 | - | - | 0.001 | 0.0308 | - | - |
| Intercept/age covariance | -0.0178 | -0.6879 | - | - | -0.0292 | -0.7776 | - | - | -0.0246 | -0.7523 | - | - |
| Residual variance | 0.6129 | 0.7829 | - | - | 0.5897 | 0.7679 | - | - | 0.5951 | 0.7714 | - | - |
| Deviance | 283415.6 |  |  |  | 283204.3 |  |  |  | 280168.7 |  |  |  |
| AIC | 283489.6 |  |  |  | 283280.3 |  |  |  | 280252.7 |  |  |  |
| BIC | 283842 |  |  |  | 283642.2 |  |  |  | 280652.5 |  |  |  |

Supplementary Table 25. Model estimates for sensitivity analysis in UK Biobank. Individuals taking anti-inflammatory medication removed.

|  | Unadjusted | | | | Sex Adjusted | | | | Fully Adjusted | | | |
| --- | --- | --- | --- | --- | --- | --- | --- | --- | --- | --- | --- | --- |
| Parameter | Estimate | SE | p-value | N | Estimate | SE | p-value | N | Estimate | SE | p-value | N |
| Intercept | 0.7482 | 0.1369 | <0.0001 | 33271 | 0.6981 | 0.1368 | <0.0001 | 33271 | 0.835 | 0.1366 | <0.0001 | 33021 |
| age | <0.0001 | 0.0023 | <0.0001 | 33271 | <0.0001 | 0.0023 | <0.0001 | 33271 | <0.0001 | 0.0023 | 0.0004 | 33021 |
| age^2 (acceleration) | <0.0001 | <0.0001 | 0.0537 | 33271 | 0.0001 | <0.0001 | 0.0436 | 33271 | <0.0001 | <0.0001 | 0.0583 | 33021 |
| Batch1 | 0.0201 | 0.1347 | 0.8811 | 33271 | 0.0198 | 0.1345 | 0.8828 | 33271 | <0.0001 | 0.1329 | 0.8754 | 33021 |
| Batch2 | 0.0006 | 0.1327 | 0.9961 | 33271 | <0.0001 | 0.1325 | 0.9921 | 33271 | <0.0001 | 0.1309 | 0.7555 | 33021 |
| Batch3 | 0.0324 | 0.1322 | 0.8064 | 33271 | 0.031 | 0.132 | 0.8146 | 33271 | <0.0001 | 0.1304 | 0.9618 | 33021 |
| Batch4 | 0.0631 | 0.1324 | 0.6337 | 33271 | 0.0618 | 0.1322 | 0.64 | 33271 | 0.0187 | 0.1307 | 0.8859 | 33021 |
| Batch5 | 0.0287 | 0.1324 | 0.8283 | 33271 | 0.0277 | 0.1322 | 0.8341 | 33271 | <0.0001 | 0.1307 | 0.8988 | 33021 |
| Batch6 | 0.0282 | 0.1324 | 0.8316 | 33271 | 0.0271 | 0.1322 | 0.8377 | 33271 | <0.0001 | 0.1307 | 0.935 | 33021 |
| Batch7 | 0.015 | 0.1327 | 0.9102 | 33271 | 0.0136 | 0.1325 | 0.918 | 33271 | <0.0001 | 0.1309 | 0.8314 | 33021 |
| Assessment_centre_baselineBirmingham | <0.0001 | 0.036 | 0.2788 | 33271 | <0.0001 | 0.036 | 0.3452 | 33271 | 0.142 | 0.0369 | 0.0001 | 33021 |
| Assessment_centre_baselineBristol | <0.0001 | 0.0329 | <0.0001 | 33271 | <0.0001 | 0.0329 | <0.0001 | 33271 | 0.0684 | 0.0348 | 0.0492 | 33021 |
| Assessment_centre_baselineBury | <0.0001 | 0.0346 | <0.0001 | 33271 | <0.0001 | 0.0345 | <0.0001 | 33271 | 0.035 | 0.0361 | 0.3326 | 33021 |
| Assessment_centre_baselineCardiff | <0.0001 | 0.0376 | 0.0608 | 33271 | <0.0001 | 0.0376 | 0.0729 | 33271 | 0.1532 | 0.0391 | <0.0001 | 33021 |
| Assessment_centre_baselineCroydon | <0.0001 | 0.0353 | 0.0679 | 33271 | <0.0001 | 0.0353 | 0.0702 | 33271 | 0.1116 | 0.036 | 0.0019 | 33021 |
| Assessment_centre_baselineEdinburgh | <0.0001 | 0.0381 | <0.0001 | 33271 | <0.0001 | 0.0381 | <0.0001 | 33271 | 0.0458 | 0.0395 | 0.2457 | 33021 |
| Assessment_centre_baselineGlasgow | <0.0001 | 0.0377 | 0.0046 | 33271 | <0.0001 | 0.0377 | 0.0057 | 33271 | 0.0642 | 0.0385 | 0.0952 | 33021 |
| Assessment_centre_baselineHounslow | <0.0001 | 0.0346 | <0.0001 | 33271 | <0.0001 | 0.0346 | <0.0001 | 33271 | 0.0317 | 0.0353 | 0.3681 | 33021 |
| Assessment_centre_baselineLeeds | <0.0001 | 0.0321 | <0.0001 | 33271 | <0.0001 | 0.032 | <0.0001 | 33271 | 0.0597 | 0.0337 | 0.077 | 33021 |
| Assessment_centre_baselineLiverpool | <0.0001 | 0.0349 | 0.0001 | 33271 | <0.0001 | 0.0349 | 0.0002 | 33271 | 0.0678 | 0.0362 | 0.0608 | 33021 |
| Assessment_centre_baselineManchester | <0.0001 | 0.0398 | 0.0781 | 33271 | <0.0001 | 0.0397 | 0.1082 | 33271 | 0.1137 | 0.0405 | 0.005 | 33021 |
| Assessment_centre_baselineMiddlesborough | <0.0001 | 0.0361 | <0.0001 | 33271 | <0.0001 | 0.0361 | 0.0001 | 33271 | 0.0787 | 0.0375 | 0.0359 | 33021 |
| Assessment_centre_baselineNewcastle | <0.0001 | 0.0339 | <0.0001 | 33271 | <0.0001 | 0.0338 | <0.0001 | 33271 | 0.0592 | 0.0351 | 0.0914 | 33021 |
| Assessment_centre_baselineNottingham | <0.0001 | 0.0329 | <0.0001 | 33271 | <0.0001 | 0.0329 | 0.0001 | 33271 | 0.1049 | 0.0346 | 0.0025 | 33021 |
| Assessment_centre_baselineOxford | <0.0001 | 0.0413 | 0.0001 | 33271 | <0.0001 | 0.0412 | 0.0001 | 33271 | 0.0777 | 0.0425 | 0.0674 | 33021 |
| Assessment_centre_baselineReading | <0.0001 | 0.0351 | <0.0001 | 33271 | <0.0001 | 0.0351 | <0.0001 | 33271 | 0.0683 | 0.0372 | 0.0668 | 33021 |
| Assessment_centre_baselineSheffield | <0.0001 | 0.0344 | 0.0009 | 33271 | <0.0001 | 0.0343 | 0.0011 | 33271 | 0.1061 | 0.0357 | 0.003 | 33021 |
| Assessment_centre_baselineStoke | <0.0001 | 0.0381 | 0.0011 | 33271 | <0.0001 | 0.0381 | 0.0024 | 33271 | 0.1215 | 0.0397 | 0.0022 | 33021 |
| Assessment_centre_baselineSwansea | 0.0098 | 0.0986 | 0.921 | 33271 | 0.012 | 0.0985 | 0.9026 | 33271 | 0.1711 | 0.0978 | 0.0801 | 33021 |
| Assessment_centre_baselineWrexham | 0.1148 | 0.1708 | 0.5016 | 33271 | 0.1167 | 0.1706 | 0.494 | 33271 | 0.3237 | 0.1689 | 0.0554 | 33021 |
| IL6_tertileMiddle | 0.1888 | 0.0414 | <0.0001 | 33271 | 0.1893 | 0.0414 | <0.0001 | 33271 | 0.1339 | 0.0412 | 0.0012 | 33021 |
| IL6_tertileTop | 0.3089 | 0.0477 | <0.0001 | 33271 | 0.3054 | 0.0477 | <0.0001 | 33271 | 0.1973 | 0.0477 | <0.0001 | 33021 |
| age:IL6_tertileMiddle | <0.0001 | 0.0035 | 0.0005 | 33271 | <0.0001 | 0.0035 | 0.0005 | 33271 | <0.0001 | 0.0035 | 0.0012 | 33021 |
| age:IL6_tertileTop | <0.0001 | 0.004 | <0.0001 | 33271 | <0.0001 | 0.004 | <0.0001 | 33271 | <0.0001 | 0.004 | <0.0001 | 33021 |
| age^2 (acceleration):IL6_tertileMiddle | 0.0002 | <0.0001 | 0.0035 | 33271 | 0.0002 | <0.0001 | 0.0034 | 33271 | 0.0002 | <0.0001 | 0.0048 | 33021 |
| age^2 (acceleration):IL6_tertileTop | 0.0003 | <0.0001 | 0.0003 | 33271 | 0.0003 | <0.0001 | 0.0003 | 33271 | 0.0003 | <0.0001 | 0.0002 | 33021 |
| SexFemale | - | - | - | - | 0.0927 | 0.01 | <0.0001 | 33271 | 0.1192 | 0.0101 | <0.0001 | 33021 |
| smoking_statusNever | - | - | - | - | - | - | - | - | <0.0001 | 0.0179 | <0.0001 | 33021 |
| smoking_statusPrevious | - | - | - | - | - | - | - | - | <0.0001 | 0.0187 | <0.0001 | 33021 |
| Townsend | - | - | - | - | - | - | - | - | 0.107 | 0.0058 | <0.0001 | 33021 |
| BMI | - | - | - | - | - | - | - | - | 0.0602 | 0.0056 | <0.0001 | 33021 |
| Intercept variance | 1.2073 | 1.0988 | - | - | 1.2103 | 1.1001 | - | - | 1.153 | 1.0738 | - | - |
| Age (slope) variance | 0.001 | 0.0321 | - | - | 0.001 | 0.0322 | - | - | 0.001 | 0.0318 | - | - |
| Intercept/age covariance | -0.0278 | -0.7878 | - | - | -0.0279 | -0.7897 | - | - | -0.0268 | -0.7859 | - | - |
| Residual variance | 0.5671 | 0.753 | - | - | 0.567 | 0.753 | - | - | 0.5657 | 0.7521 | - | - |
| Deviance | 238383 |  |  |  | 238298 |  |  |  | 235957 |  |  |  |
| AIC | 238463 |  |  |  | 238380 |  |  |  | 236047 |  |  |  |
| BIC | 238838 |  |  |  | 238764 |  |  |  | 236468 |  |  |  |

Supplementary Table 26. Model estimates for sensitivity analysis in UK Biobank. Individuals with inflammatory conditions removed.

|  | Unadjusted | | | | Sex Adjusted | | | | Fully Adjusted | | | |
| --- | --- | --- | --- | --- | --- | --- | --- | --- | --- | --- | --- | --- |
| Parameter | Estimate | SE | p-value | N | Estimate | SE | p-value | N | Estimate | SE | p-value | N |
| Intercept | 0.7482 | 0.1369 | <0.0001 | 33271 | 0.6981 | 0.1368 | <0.0001 | 33271 | 0.835 | 0.1366 | <0.0001 | 33021 |
| age | <0.0001 | 0.0023 | <0.0001 | 33271 | <0.0001 | 0.0023 | <0.0001 | 33271 | <0.0001 | 0.0023 | 0.0004 | 33021 |
| age^2 (acceleration) | <0.0001 | <0.0001 | 0.0537 | 33271 | 0.0001 | <0.0001 | 0.0436 | 33271 | <0.0001 | <0.0001 | 0.0583 | 33021 |
| Batch1 | 0.0201 | 0.1347 | 0.8811 | 33271 | 0.0198 | 0.1345 | 0.8828 | 33271 | <0.0001 | 0.1329 | 0.8754 | 33021 |
| Batch2 | 0.0006 | 0.1327 | 0.9961 | 33271 | <0.0001 | 0.1325 | 0.9921 | 33271 | <0.0001 | 0.1309 | 0.7555 | 33021 |
| Batch3 | 0.0324 | 0.1322 | 0.8064 | 33271 | 0.031 | 0.132 | 0.8146 | 33271 | <0.0001 | 0.1304 | 0.9618 | 33021 |
| Batch4 | 0.0631 | 0.1324 | 0.6337 | 33271 | 0.0618 | 0.1322 | 0.64 | 33271 | 0.0187 | 0.1307 | 0.8859 | 33021 |
| Batch5 | 0.0287 | 0.1324 | 0.8283 | 33271 | 0.0277 | 0.1322 | 0.8341 | 33271 | <0.0001 | 0.1307 | 0.8988 | 33021 |
| Batch6 | 0.0282 | 0.1324 | 0.8316 | 33271 | 0.0271 | 0.1322 | 0.8377 | 33271 | <0.0001 | 0.1307 | 0.935 | 33021 |
| Batch7 | 0.015 | 0.1327 | 0.9102 | 33271 | 0.0136 | 0.1325 | 0.918 | 33271 | <0.0001 | 0.1309 | 0.8314 | 33021 |
| Assessment_centre_baselineBirmingham | <0.0001 | 0.036 | 0.2788 | 33271 | <0.0001 | 0.036 | 0.3452 | 33271 | 0.142 | 0.0369 | 0.0001 | 33021 |
| Assessment_centre_baselineBristol | <0.0001 | 0.0329 | <0.0001 | 33271 | <0.0001 | 0.0329 | <0.0001 | 33271 | 0.0684 | 0.0348 | 0.0492 | 33021 |
| Assessment_centre_baselineBury | <0.0001 | 0.0346 | <0.0001 | 33271 | <0.0001 | 0.0345 | <0.0001 | 33271 | 0.035 | 0.0361 | 0.3326 | 33021 |
| Assessment_centre_baselineCardiff | <0.0001 | 0.0376 | 0.0608 | 33271 | <0.0001 | 0.0376 | 0.0729 | 33271 | 0.1532 | 0.0391 | <0.0001 | 33021 |
| Assessment_centre_baselineCroydon | <0.0001 | 0.0353 | 0.0679 | 33271 | <0.0001 | 0.0353 | 0.0702 | 33271 | 0.1116 | 0.036 | 0.0019 | 33021 |
| Assessment_centre_baselineEdinburgh | <0.0001 | 0.0381 | <0.0001 | 33271 | <0.0001 | 0.0381 | <0.0001 | 33271 | 0.0458 | 0.0395 | 0.2457 | 33021 |
| Assessment_centre_baselineGlasgow | <0.0001 | 0.0377 | 0.0046 | 33271 | <0.0001 | 0.0377 | 0.0057 | 33271 | 0.0642 | 0.0385 | 0.0952 | 33021 |
| Assessment_centre_baselineHounslow | <0.0001 | 0.0346 | <0.0001 | 33271 | <0.0001 | 0.0346 | <0.0001 | 33271 | 0.0317 | 0.0353 | 0.3681 | 33021 |
| Assessment_centre_baselineLeeds | <0.0001 | 0.0321 | <0.0001 | 33271 | <0.0001 | 0.032 | <0.0001 | 33271 | 0.0597 | 0.0337 | 0.077 | 33021 |
| Assessment_centre_baselineLiverpool | <0.0001 | 0.0349 | 0.0001 | 33271 | <0.0001 | 0.0349 | 0.0002 | 33271 | 0.0678 | 0.0362 | 0.0608 | 33021 |
| Assessment_centre_baselineManchester | <0.0001 | 0.0398 | 0.0781 | 33271 | <0.0001 | 0.0397 | 0.1082 | 33271 | 0.1137 | 0.0405 | 0.005 | 33021 |
| Assessment_centre_baselineMiddlesborough | <0.0001 | 0.0361 | <0.0001 | 33271 | <0.0001 | 0.0361 | 0.0001 | 33271 | 0.0787 | 0.0375 | 0.0359 | 33021 |
| Assessment_centre_baselineNewcastle | <0.0001 | 0.0339 | <0.0001 | 33271 | <0.0001 | 0.0338 | <0.0001 | 33271 | 0.0592 | 0.0351 | 0.0914 | 33021 |
| Assessment_centre_baselineNottingham | <0.0001 | 0.0329 | <0.0001 | 33271 | <0.0001 | 0.0329 | 0.0001 | 33271 | 0.1049 | 0.0346 | 0.0025 | 33021 |
| Assessment_centre_baselineOxford | <0.0001 | 0.0413 | 0.0001 | 33271 | <0.0001 | 0.0412 | 0.0001 | 33271 | 0.0777 | 0.0425 | 0.0674 | 33021 |
| Assessment_centre_baselineReading | <0.0001 | 0.0351 | <0.0001 | 33271 | <0.0001 | 0.0351 | <0.0001 | 33271 | 0.0683 | 0.0372 | 0.0668 | 33021 |
| Assessment_centre_baselineSheffield | <0.0001 | 0.0344 | 0.0009 | 33271 | <0.0001 | 0.0343 | 0.0011 | 33271 | 0.1061 | 0.0357 | 0.003 | 33021 |
| Assessment_centre_baselineStoke | <0.0001 | 0.0381 | 0.0011 | 33271 | <0.0001 | 0.0381 | 0.0024 | 33271 | 0.1215 | 0.0397 | 0.0022 | 33021 |
| Assessment_centre_baselineSwansea | 0.0098 | 0.0986 | 0.921 | 33271 | 0.012 | 0.0985 | 0.9026 | 33271 | 0.1711 | 0.0978 | 0.0801 | 33021 |
| Assessment_centre_baselineWrexham | 0.1148 | 0.1708 | 0.5016 | 33271 | 0.1167 | 0.1706 | 0.494 | 33271 | 0.3237 | 0.1689 | 0.0554 | 33021 |
| IL6_tertileMiddle | 0.1888 | 0.0414 | <0.0001 | 33271 | 0.1893 | 0.0414 | <0.0001 | 33271 | 0.1339 | 0.0412 | 0.0012 | 33021 |
| IL6_tertileTop | 0.3089 | 0.0477 | <0.0001 | 33271 | 0.3054 | 0.0477 | <0.0001 | 33271 | 0.1973 | 0.0477 | <0.0001 | 33021 |
| age:IL6_tertileMiddle | <0.0001 | 0.0035 | 0.0005 | 33271 | <0.0001 | 0.0035 | 0.0005 | 33271 | <0.0001 | 0.0035 | 0.0012 | 33021 |
| age:IL6_tertileTop | <0.0001 | 0.004 | <0.0001 | 33271 | <0.0001 | 0.004 | <0.0001 | 33271 | <0.0001 | 0.004 | <0.0001 | 33021 |
| age^2 (acceleration):IL6_tertileMiddle | 0.0002 | <0.0001 | 0.0035 | 33271 | 0.0002 | <0.0001 | 0.0034 | 33271 | 0.0002 | <0.0001 | 0.0048 | 33021 |
| age^2 (acceleration):IL6_tertileTop | 0.0003 | <0.0001 | 0.0003 | 33271 | 0.0003 | <0.0001 | 0.0003 | 33271 | 0.0003 | <0.0001 | 0.0002 | 33021 |
| SexFemale | - | - | - | - | 0.0927 | 0.01 | <0.0001 | 33271 | 0.1192 | 0.0101 | <0.0001 | 33021 |
| smoking_statusNever | - | - | - | - | - | - | - | - | <0.0001 | 0.0179 | <0.0001 | 33021 |
| smoking_statusPrevious | - | - | - | - | - | - | - | - | <0.0001 | 0.0187 | <0.0001 | 33021 |
| Townsend | - | - | - | - | - | - | - | - | 0.107 | 0.0058 | <0.0001 | 33021 |
| BMI | - | - | - | - | - | - | - | - | 0.0602 | 0.0056 | <0.0001 | 33021 |
| Intercept variance | 1.2073 | 1.0988 | - | - | 1.2103 | 1.1001 | - | - | 1.153 | 1.0738 | - | - |
| Age (slope) variance | 0.001 | 0.0321 | - | - | 0.001 | 0.0322 | - | - | 0.001 | 0.0318 | - | - |
| Intercept/age covariance | -0.0278 | -0.7878 | - | - | -0.0279 | -0.7897 | - | - | -0.0268 | -0.7859 | - | - |
| Residual variance | 0.5671 | 0.753 | - | - | 0.567 | 0.753 | - | - | 0.5657 | 0.7521 | - | - |
| Deviance | 238382.6 |  |  |  | 238297.5 |  |  |  | 235956.7 |  |  |  |
| AIC | 238462.6 |  |  |  | 238379.5 |  |  |  | 236046.7 |  |  |  |
| BIC | 238837.5 |  |  |  | 238763.8 |  |  |  | 236468.3 |  |  |  |

Supplementary Table 27. Model estimates for sensitivity analysis in UK Biobank. Individuals with BMI >= 40 removed.

|  | Unadjusted | | | | Sex Adjusted | | | | Fully Adjusted | | | |
| --- | --- | --- | --- | --- | --- | --- | --- | --- | --- | --- | --- | --- |
| Parameter | Estimate | SE | p-value | N | Estimate | SE | p-value | N | Estimate | SE | p-value | N |
| Intercept | 1.2575 | 0.3101 | <0.0001 | 9226 | 1.2027 | 0.3098 | 0.0001 | 9226 | 1.4748 | 0.3083 | <0.0001 | 9192 |
| age | <0.0001 | 0.0085 | 0.3573 | 9226 | <0.0001 | 0.0085 | 0.3383 | 9226 | <0.0001 | 0.0085 | 0.4784 | 9192 |
| age^2 (acceleration) | <0.0001 | 0.0002 | 0.6479 | 9226 | <0.0001 | 0.0002 | 0.6441 | 9226 | <0.0001 | 0.0002 | 0.6768 | 9192 |
| Batch1 | <0.0001 | 0.2963 | 0.2485 | 9226 | <0.0001 | 0.2959 | 0.2511 | 9226 | <0.0001 | 0.2915 | 0.1021 | 9192 |
| Batch2 | <0.0001 | 0.2915 | 0.3177 | 9226 | <0.0001 | 0.291 | 0.319 | 9226 | <0.0001 | 0.2868 | 0.1444 | 9192 |
| Batch3 | <0.0001 | 0.2904 | 0.3826 | 9226 | <0.0001 | 0.29 | 0.3813 | 9226 | <0.0001 | 0.2857 | 0.1878 | 9192 |
| Batch4 | <0.0001 | 0.2909 | 0.393 | 9226 | <0.0001 | 0.2904 | 0.3939 | 9226 | <0.0001 | 0.2862 | 0.1953 | 9192 |
| Batch5 | <0.0001 | 0.2909 | 0.2818 | 9226 | <0.0001 | 0.2905 | 0.2823 | 9226 | <0.0001 | 0.2862 | 0.1316 | 9192 |
| Batch6 | <0.0001 | 0.291 | 0.4 | 9226 | <0.0001 | 0.2905 | 0.4 | 9226 | <0.0001 | 0.2862 | 0.1922 | 9192 |
| Batch7 | <0.0001 | 0.2914 | 0.2538 | 9226 | <0.0001 | 0.291 | 0.256 | 9226 | <0.0001 | 0.2867 | 0.1214 | 9192 |
| Assessment_centre_baselineBirmingham | <0.0001 | 0.0855 | 0.979 | 9226 | 0.0051 | 0.0853 | 0.9527 | 9226 | 0.2265 | 0.0865 | 0.0088 | 9192 |
| Assessment_centre_baselineBristol | <0.0001 | 0.0815 | 0.0033 | 9226 | <0.0001 | 0.0813 | 0.0045 | 9226 | 0.0685 | 0.0846 | 0.4186 | 9192 |
| Assessment_centre_baselineBury | <0.0001 | 0.0828 | 0.0096 | 9226 | <0.0001 | 0.0826 | 0.0123 | 9226 | 0.0629 | 0.0852 | 0.4599 | 9192 |
| Assessment_centre_baselineCardiff | <0.0001 | 0.0875 | 0.2872 | 9226 | <0.0001 | 0.0873 | 0.3535 | 9226 | 0.2185 | 0.0903 | 0.0155 | 9192 |
| Assessment_centre_baselineCroydon | <0.0001 | 0.0861 | 0.4272 | 9226 | <0.0001 | 0.0859 | 0.4423 | 9226 | 0.1356 | 0.0868 | 0.118 | 9192 |
| Assessment_centre_baselineEdinburgh | <0.0001 | 0.0954 | 0.0909 | 9226 | <0.0001 | 0.0952 | 0.1022 | 9226 | 0.1085 | 0.097 | 0.2635 | 9192 |
| Assessment_centre_baselineGlasgow | <0.0001 | 0.0895 | 0.2428 | 9226 | <0.0001 | 0.0893 | 0.3032 | 9226 | 0.0918 | 0.0899 | 0.3074 | 9192 |
| Assessment_centre_baselineHounslow | <0.0001 | 0.0866 | 0.4324 | 9226 | <0.0001 | 0.0864 | 0.516 | 9226 | 0.1475 | 0.0873 | 0.091 | 9192 |
| Assessment_centre_baselineLeeds | <0.0001 | 0.0788 | 0.0061 | 9226 | <0.0001 | 0.0786 | 0.0084 | 9226 | 0.0591 | 0.0814 | 0.4676 | 9192 |
| Assessment_centre_baselineLiverpool | <0.0001 | 0.0828 | 0.0161 | 9226 | <0.0001 | 0.0827 | 0.0213 | 9226 | 0.0614 | 0.0847 | 0.4687 | 9192 |
| Assessment_centre_baselineManchester | <0.0001 | 0.0943 | 0.7882 | 9226 | <0.0001 | 0.0941 | 0.8356 | 9226 | 0.1888 | 0.0952 | 0.0472 | 9192 |
| Assessment_centre_baselineMiddlesborough | <0.0001 | 0.0866 | 0.4004 | 9226 | <0.0001 | 0.0864 | 0.4227 | 9226 | 0.1941 | 0.0884 | 0.0281 | 9192 |
| Assessment_centre_baselineNewcastle | <0.0001 | 0.0814 | 0.0087 | 9226 | <0.0001 | 0.0813 | 0.0119 | 9226 | 0.0314 | 0.0829 | 0.7046 | 9192 |
| Assessment_centre_baselineNottingham | <0.0001 | 0.0804 | 0.0675 | 9226 | <0.0001 | 0.0803 | 0.0873 | 9226 | 0.1427 | 0.0831 | 0.0857 | 9192 |
| Assessment_centre_baselineOxford | <0.0001 | 0.1094 | 0.0132 | 9226 | <0.0001 | 0.1092 | 0.0125 | 9226 | <0.0001 | 0.1107 | 0.8686 | 9192 |
| Assessment_centre_baselineReading | <0.0001 | 0.0887 | 0.0088 | 9226 | <0.0001 | 0.0886 | 0.011 | 9226 | 0.1018 | 0.0923 | 0.2702 | 9192 |
| Assessment_centre_baselineSheffield | <0.0001 | 0.0826 | 0.2197 | 9226 | <0.0001 | 0.0825 | 0.2609 | 9226 | 0.1753 | 0.0848 | 0.0386 | 9192 |
| Assessment_centre_baselineStoke | <0.0001 | 0.0903 | 0.3196 | 9226 | <0.0001 | 0.0902 | 0.4278 | 9226 | 0.2319 | 0.093 | 0.0126 | 9192 |
| Assessment_centre_baselineSwansea | 0.0661 | 0.2059 | 0.7481 | 9226 | 0.0751 | 0.2055 | 0.7148 | 9226 | 0.2666 | 0.2035 | 0.1902 | 9192 |
| Assessment_centre_baselineWrexham | <0.0001 | 0.2899 | 0.2137 | 9226 | <0.0001 | 0.2894 | 0.2002 | 9226 | <0.0001 | 0.2863 | 0.6198 | 9192 |
| IL6_tertileMiddle | 0.1028 | 0.1204 | 0.3933 | 9226 | 0.0916 | 0.1204 | 0.4464 | 9226 | 0.0543 | 0.1194 | 0.6496 | 9192 |
| IL6_tertileTop | 0.2313 | 0.1159 | 0.0459 | 9226 | 0.208 | 0.1159 | 0.0727 | 9226 | 0.0868 | 0.1153 | 0.4515 | 9192 |
| age:IL6_tertileMiddle | <0.0001 | 0.0106 | 0.1514 | 9226 | <0.0001 | 0.0106 | 0.148 | 9226 | <0.0001 | 0.0105 | 0.1632 | 9192 |
| age:IL6_tertileTop | <0.0001 | 0.0102 | 0.0996 | 9226 | <0.0001 | 0.0102 | 0.1099 | 9226 | <0.0001 | 0.0101 | 0.1319 | 9192 |
| age^2 (acceleration):IL6_tertileMiddle | 0.0003 | 0.0002 | 0.1833 | 9226 | 0.0003 | 0.0002 | 0.1717 | 9226 | 0.0003 | 0.0002 | 0.1783 | 9192 |
| age^2 (acceleration):IL6_tertileTop | 0.0003 | 0.0002 | 0.1649 | 9226 | 0.0003 | 0.0002 | 0.1684 | 9226 | 0.0003 | 0.0002 | 0.1627 | 9192 |
| SexFemale | - | - | - | - | 0.121 | 0.0231 | <0.0001 | 9226 | 0.1102 | 0.0232 | <0.0001 | 9192 |
| smoking_statusNever | - | - | - | - | - | - | - | - | <0.0001 | 0.042 | <0.0001 | 9192 |
| smoking_statusPrevious | - | - | - | - | - | - | - | - | <0.0001 | 0.0429 | <0.0001 | 9192 |
| Townsend | - | - | - | - | - | - | - | - | 0.1411 | 0.0128 | <0.0001 | 9192 |
| BMI | - | - | - | - | - | - | - | - | 0.1097 | 0.0119 | <0.0001 | 9192 |
| Intercept variance | 1.6173 | 1.2717 | - | - | 1.6139 | 1.2704 | - | - | 1.5442 | 1.2426 | - | - |
| Age (slope) variance | 0.0014 | 0.037 | - | - | 0.0014 | 0.0369 | - | - | 0.0014 | 0.0368 | - | - |
| Intercept/age covariance | -0.0356 | -0.7574 | - | - | -0.0355 | -0.7585 | - | - | -0.0347 | -0.7578 | - | - |
| Residual variance | 0.7394 | 0.8599 | - | - | 0.7398 | 0.8601 | - | - | 0.7348 | 0.8572 | - | - |
| Deviance | 64496.11 |  |  |  | 64468.76 |  |  |  | 63926.07 |  |  |  |
| AIC | 64576.11 |  |  |  | 64550.76 |  |  |  | 64016.07 |  |  |  |
| BIC | 64894.37 |  |  |  | 64876.97 |  |  |  | 64373.97 |  |  |  |

Supplementary Table 28. Model estimates for sensitivity analysis in UK Biobank. Individuals that remained alive after initial baseline appointment only.

|  | Unadjusted | | | | Sex Adjusted | | | | Fully Adjusted | | | |
| --- | --- | --- | --- | --- | --- | --- | --- | --- | --- | --- | --- | --- |
| Parameter | Estimate | SE | p-value | N | Estimate | SE | p-value | N | Estimate | SE | p-value | N |
| Intercept | 0.7706 | 0.1414 | <0.0001 | 36798 | 0.7177 | 0.1413 | <0.0001 | 36798 | 0.8651 | 0.1386 | <0.0001 | 36515 |
| age | <0.0001 | 0.0022 | 0.0003 | 36798 | <0.0001 | 0.0022 | 0.0002 | 36798 | <0.0001 | 0.0021 | 0.0003 | 36515 |
| age^2 (acceleration) | <0.0001 | <0.0001 | 0.1169 | 36798 | <0.0001 | <0.0001 | 0.0985 | 36798 | <0.0001 | <0.0001 | 0.0515 | 36515 |
| Batch1 | 0.0102 | 0.1396 | 0.9415 | 36798 | 0.0142 | 0.1393 | 0.9191 | 36798 | <0.0001 | 0.1356 | 0.8559 | 36515 |
| Batch2 | <0.0001 | 0.1377 | 0.9497 | 36798 | <0.0001 | 0.1375 | 0.9701 | 36798 | <0.0001 | 0.1338 | 0.749 | 36515 |
| Batch3 | 0.0208 | 0.1373 | 0.8793 | 36798 | 0.0244 | 0.1371 | 0.859 | 36798 | <0.0001 | 0.1334 | 0.9346 | 36515 |
| Batch4 | 0.0521 | 0.1375 | 0.7045 | 36798 | 0.0553 | 0.1373 | 0.687 | 36798 | 0.0161 | 0.1336 | 0.9038 | 36515 |
| Batch5 | 0.008 | 0.1375 | 0.9537 | 36798 | 0.0122 | 0.1373 | 0.9292 | 36798 | <0.0001 | 0.1336 | 0.8168 | 36515 |
| Batch6 | 0.0209 | 0.1375 | 0.879 | 36798 | 0.0246 | 0.1373 | 0.858 | 36798 | <0.0001 | 0.1336 | 0.9365 | 36515 |
| Batch7 | 0.0092 | 0.1377 | 0.947 | 36798 | 0.0126 | 0.1375 | 0.9268 | 36798 | <0.0001 | 0.1338 | 0.8349 | 36515 |
| Assessment_centre_baselineBirmingham | <0.0001 | 0.0352 | 0.3531 | 36798 | <0.0001 | 0.0351 | 0.4151 | 36798 | 0.1508 | 0.0354 | <0.0001 | 36515 |
| Assessment_centre_baselineBristol | <0.0001 | 0.0323 | <0.0001 | 36798 | <0.0001 | 0.0322 | <0.0001 | 36798 | 0.0641 | 0.0335 | 0.0557 | 36515 |
| Assessment_centre_baselineBury | <0.0001 | 0.0338 | <0.0001 | 36798 | <0.0001 | 0.0338 | <0.0001 | 36798 | 0.0496 | 0.0348 | 0.1538 | 36515 |
| Assessment_centre_baselineCardiff | <0.0001 | 0.037 | 0.1603 | 36798 | <0.0001 | 0.0369 | 0.1785 | 36798 | 0.1774 | 0.0379 | <0.0001 | 36515 |
| Assessment_centre_baselineCroydon | <0.0001 | 0.0344 | 0.0721 | 36798 | <0.0001 | 0.0343 | 0.0721 | 36798 | 0.1203 | 0.0345 | 0.0005 | 36515 |
| Assessment_centre_baselineEdinburgh | <0.0001 | 0.0378 | <0.0001 | 36798 | <0.0001 | 0.0378 | <0.0001 | 36798 | 0.0409 | 0.0386 | 0.2885 | 36515 |
| Assessment_centre_baselineGlasgow | <0.0001 | 0.0374 | 0.0099 | 36798 | <0.0001 | 0.0374 | 0.0103 | 36798 | 0.0806 | 0.0376 | 0.0319 | 36515 |
| Assessment_centre_baselineHounslow | <0.0001 | 0.0338 | 0.0003 | 36798 | <0.0001 | 0.0338 | 0.0003 | 36798 | 0.059 | 0.0339 | 0.082 | 36515 |
| Assessment_centre_baselineLeeds | <0.0001 | 0.0315 | <0.0001 | 36798 | <0.0001 | 0.0314 | <0.0001 | 36798 | 0.0524 | 0.0326 | 0.1076 | 36515 |
| Assessment_centre_baselineLiverpool | <0.0001 | 0.0342 | 0.0002 | 36798 | <0.0001 | 0.0341 | 0.0003 | 36798 | 0.0898 | 0.0348 | 0.01 | 36515 |
| Assessment_centre_baselineManchester | <0.0001 | 0.0389 | 0.2393 | 36798 | <0.0001 | 0.0389 | 0.2762 | 36798 | 0.1487 | 0.039 | 0.0001 | 36515 |
| Assessment_centre_baselineMiddlesborough | <0.0001 | 0.0354 | <0.0001 | 36798 | <0.0001 | 0.0354 | <0.0001 | 36798 | 0.0885 | 0.0361 | 0.0143 | 36515 |
| Assessment_centre_baselineNewcastle | <0.0001 | 0.033 | <0.0001 | 36798 | <0.0001 | 0.033 | <0.0001 | 36798 | 0.0569 | 0.0336 | 0.091 | 36515 |
| Assessment_centre_baselineNottingham | <0.0001 | 0.0323 | <0.0001 | 36798 | <0.0001 | 0.0322 | <0.0001 | 36798 | 0.1167 | 0.0334 | 0.0005 | 36515 |
| Assessment_centre_baselineOxford | <0.0001 | 0.0405 | <0.0001 | 36798 | <0.0001 | 0.0404 | <0.0001 | 36798 | 0.06 | 0.0411 | 0.1443 | 36515 |
| Assessment_centre_baselineReading | <0.0001 | 0.0345 | <0.0001 | 36798 | <0.0001 | 0.0345 | <0.0001 | 36798 | 0.0639 | 0.036 | 0.0759 | 36515 |
| Assessment_centre_baselineSheffield | <0.0001 | 0.0334 | 0.0001 | 36798 | <0.0001 | 0.0334 | 0.0001 | 36798 | 0.0968 | 0.0342 | 0.0046 | 36515 |
| Assessment_centre_baselineStoke | <0.0001 | 0.0373 | 0.0003 | 36798 | <0.0001 | 0.0373 | 0.0006 | 36798 | 0.1225 | 0.0383 | 0.0014 | 36515 |
| Assessment_centre_baselineSwansea | <0.0001 | 0.098 | 0.8877 | 36798 | <0.0001 | 0.0978 | 0.9045 | 36798 | 0.1623 | 0.096 | 0.091 | 36515 |
| Assessment_centre_baselineWrexham | <0.0001 | 0.1628 | 0.9694 | 36798 | <0.0001 | 0.1626 | 0.9868 | 36798 | 0.1939 | 0.1577 | 0.2187 | 36515 |
| IL6_tertileMiddle | 0.1976 | 0.04 | <0.0001 | 36798 | 0.199 | 0.04 | <0.0001 | 36798 | 0.1348 | 0.0374 | 0.0003 | 36515 |
| IL6_tertileTop | 0.2959 | 0.0453 | <0.0001 | 36798 | 0.2941 | 0.0454 | <0.0001 | 36798 | 0.1775 | 0.0428 | <0.0001 | 36515 |
| age:IL6_tertileMiddle | <0.0001 | 0.0034 | <0.0001 | 36798 | <0.0001 | 0.0034 | <0.0001 | 36798 | <0.0001 | 0.0032 | 0.0003 | 36515 |
| age:IL6_tertileTop | <0.0001 | 0.0038 | <0.0001 | 36798 | <0.0001 | 0.0038 | <0.0001 | 36798 | <0.0001 | 0.0036 | <0.0001 | 36515 |
| age^2 (acceleration):IL6_tertileMiddle | 0.0002 | <0.0001 | 0.0009 | 36798 | 0.0002 | <0.0001 | 0.0008 | 36798 | 0.0002 | <0.0001 | 0.0016 | 36515 |
| age^2 (acceleration):IL6_tertileTop | 0.0003 | <0.0001 | <0.0001 | 36798 | 0.0003 | <0.0001 | <0.0001 | 36798 | 0.0003 | <0.0001 | <0.0001 | 36515 |
| SexFemale | - | - | - | - | 0.0901 | 0.0098 | <0.0001 | 36798 | 0.1173 | 0.0098 | <0.0001 | 36515 |
| smoking_statusNever | - | - | - | - | - | - | - | - | <0.0001 | 0.0174 | <0.0001 | 36515 |
| smoking_statusPrevious | - | - | - | - | - | - | - | - | <0.0001 | 0.0181 | <0.0001 | 36515 |
| Townsend | - | - | - | - | - | - | - | - | 0.1159 | 0.0055 | <0.0001 | 36515 |
| BMI | - | - | - | - | - | - | - | - | 0.0672 | 0.0053 | <0.0001 | 36515 |
| Intercept variance | 1.2279 | 1.1081 | - | - | 1.2296 | 1.1089 | - | - | 0.7884 | 0.8879 | - | - |
| Age (slope) variance | 0.0011 | 0.0327 | - | - | 0.0011 | 0.0327 | - | - | 0.0005 | 0.0229 | - | - |
| Intercept/age covariance | -0.0277 | -0.7658 | - | - | -0.0278 | -0.7677 | - | - | -0.0126 | -0.6214 | - | - |
| Residual variance | 0.5858 | 0.7654 | - | - | 0.586 | 0.7655 | - | - | 0.6151 | 0.7843 | - | - |
| Deviance | 272455.4 |  |  |  | 272371.9 |  |  |  | 269788.3 |  |  |  |
| AIC | 272535.4 |  |  |  | 272453.9 |  |  |  | 269878.3 |  |  |  |
| BIC | 272915.1 |  |  |  | 272843.1 |  |  |  | 270305.2 |  |  |  |

Supplementary Table 29. Model estimates from linear regression of number of PHQ-2 questionnaires participants completed on IL-6 tertile in UK Biobank. Participants subset to those that remained alive after the initial assessment.

| term | estimate | std.error | statistic | p.value | N |
| --- | --- | --- | --- | --- | --- |
| (Intercept) | 2.864 | 0.016 | 181.578 | p<0.0001 | 36798 |
| IL6_tertileMiddle | -0.221 | 0.023 | -9.82 | p<0.0001 | 36798 |
| IL6_tertileTop | -0.438 | 0.024 | -18.588 | p<0.0001 | 36798 |

Supplementary Table 30. Model estimates for sensitivity analysis in UK Biobank. Individuals who attended only one assessment removed.

|  | Unadjusted | | | | Sex Adjusted | | | | Fullu Adjusted | | | |
| --- | --- | --- | --- | --- | --- | --- | --- | --- | --- | --- | --- | --- |
| Parameter | Estimate | SE | p-value | N | Estimate | SE | p-value | N | Estimate | SE | p-value | N |
| Intercept | 0.6506 | 0.1635 | <0.0001 | 20602 | 0.5908 | 0.1641 | 0.0003 | 20602 | 0.7468 | 0.1631 | <0.0001 | 20497 |
| age | <0.0001 | 0.0024 | 0.1166 | 20602 | <0.0001 | 0.0024 | 0.1348 | 20602 | <0.0001 | 0.0024 | 0.1452 | 20497 |
| age^2 (acceleration) | <0.0001 | <0.0001 | 0.5147 | 20602 | <0.0001 | <0.0001 | 0.5516 | 20602 | <0.0001 | <0.0001 | 0.4778 | 20497 |
| Batch1 | 0.0445 | 0.1616 | 0.7832 | 20602 | 0.0457 | 0.162 | 0.778 | 20602 | 0.0325 | 0.1592 | 0.8382 | 20497 |
| Batch2 | 0.0113 | 0.1594 | 0.9434 | 20602 | 0.013 | 0.1598 | 0.9354 | 20602 | <0.0001 | 0.1571 | 0.9927 | 20497 |
| Batch3 | 0.0592 | 0.1589 | 0.7097 | 20602 | 0.0606 | 0.1593 | 0.7037 | 20602 | 0.0497 | 0.1566 | 0.7509 | 20497 |
| Batch4 | 0.0626 | 0.1592 | 0.6943 | 20602 | 0.0646 | 0.1596 | 0.6854 | 20602 | 0.0521 | 0.1568 | 0.7399 | 20497 |
| Batch5 | 0.0417 | 0.1591 | 0.7932 | 20602 | 0.0434 | 0.1595 | 0.7857 | 20602 | 0.0286 | 0.1568 | 0.8555 | 20497 |
| Batch6 | 0.0446 | 0.1592 | 0.7793 | 20602 | 0.0464 | 0.1596 | 0.7714 | 20602 | 0.037 | 0.1568 | 0.8135 | 20497 |
| Batch7 | 0.0416 | 0.1594 | 0.7941 | 20602 | 0.0434 | 0.1598 | 0.7857 | 20602 | 0.0299 | 0.1571 | 0.8489 | 20497 |
| Assessment_centre_baselineBirmingham | <0.0001 | 0.041 | 0.0197 | 20602 | <0.0001 | 0.0411 | 0.0276 | 20602 | 0.0761 | 0.0422 | 0.0713 | 20497 |
| Assessment_centre_baselineBristol | <0.0001 | 0.0368 | <0.0001 | 20602 | <0.0001 | 0.0369 | <0.0001 | 20602 | 0.0492 | 0.039 | 0.207 | 20497 |
| Assessment_centre_baselineBury | <0.0001 | 0.0395 | 0.0001 | 20602 | <0.0001 | 0.0396 | 0.0002 | 20602 | 0.0501 | 0.0414 | 0.2258 | 20497 |
| Assessment_centre_baselineCardiff | <0.0001 | 0.0437 | 0.4544 | 20602 | <0.0001 | 0.0437 | 0.505 | 20602 | 0.1639 | 0.0454 | 0.0003 | 20497 |
| Assessment_centre_baselineCroydon | <0.0001 | 0.0393 | 0.0024 | 20602 | <0.0001 | 0.0394 | 0.0028 | 20602 | 0.0386 | 0.0401 | 0.3366 | 20497 |
| Assessment_centre_baselineEdinburgh | <0.0001 | 0.0433 | <0.0001 | 20602 | <0.0001 | 0.0434 | <0.0001 | 20602 | 0.0417 | 0.045 | 0.3541 | 20497 |
| Assessment_centre_baselineGlasgow | <0.0001 | 0.0448 | 0.0606 | 20602 | <0.0001 | 0.0449 | 0.0669 | 20602 | 0.0834 | 0.0458 | 0.0682 | 20497 |
| Assessment_centre_baselineHounslow | <0.0001 | 0.0386 | <0.0001 | 20602 | <0.0001 | 0.0387 | <0.0001 | 20602 | 0.0026 | 0.0394 | 0.9465 | 20497 |
| Assessment_centre_baselineLeeds | <0.0001 | 0.0362 | <0.0001 | 20602 | <0.0001 | 0.0363 | <0.0001 | 20602 | 0.0352 | 0.0383 | 0.3577 | 20497 |
| Assessment_centre_baselineLiverpool | <0.0001 | 0.0396 | 0.0177 | 20602 | <0.0001 | 0.0397 | 0.021 | 20602 | 0.0976 | 0.0412 | 0.018 | 20497 |
| Assessment_centre_baselineManchester | <0.0001 | 0.0442 | 0.2499 | 20602 | <0.0001 | 0.0443 | 0.3015 | 20602 | 0.119 | 0.0452 | 0.0085 | 20497 |
| Assessment_centre_baselineMiddlesborough | <0.0001 | 0.0413 | 0.003 | 20602 | <0.0001 | 0.0414 | 0.0038 | 20602 | 0.0778 | 0.0429 | 0.0701 | 20497 |
| Assessment_centre_baselineNewcastle | <0.0001 | 0.0384 | 0.0004 | 20602 | <0.0001 | 0.0385 | 0.0006 | 20602 | 0.0512 | 0.0399 | 0.1997 | 20497 |
| Assessment_centre_baselineNottingham | <0.0001 | 0.0372 | 0.0015 | 20602 | <0.0001 | 0.0372 | 0.0021 | 20602 | 0.0992 | 0.0392 | 0.0115 | 20497 |
| Assessment_centre_baselineOxford | <0.0001 | 0.0463 | 0.0003 | 20602 | <0.0001 | 0.0464 | 0.0004 | 20602 | 0.0469 | 0.0477 | 0.3257 | 20497 |
| Assessment_centre_baselineReading | <0.0001 | 0.039 | <0.0001 | 20602 | <0.0001 | 0.0391 | <0.0001 | 20602 | 0.0368 | 0.0417 | 0.3765 | 20497 |
| Assessment_centre_baselineSheffield | <0.0001 | 0.0382 | 0.0066 | 20602 | <0.0001 | 0.0383 | 0.0087 | 20602 | 0.0982 | 0.0399 | 0.0139 | 20497 |
| Assessment_centre_baselineStoke | <0.0001 | 0.0452 | 0.014 | 20602 | <0.0001 | 0.0453 | 0.0265 | 20602 | 0.1233 | 0.0471 | 0.0088 | 20497 |
| Assessment_centre_baselineSwansea | <0.0001 | 0.1123 | 0.2634 | 20602 | <0.0001 | 0.1124 | 0.2825 | 20602 | 0.0337 | 0.1113 | 0.762 | 20497 |
| Assessment_centre_baselineWrexham | 0.0275 | 0.191 | 0.8855 | 20602 | 0.0374 | 0.1918 | 0.8454 | 20602 | 0.2245 | 0.1889 | 0.2348 | 20497 |
| IL6_tertileMiddle | 0.0683 | 0.0466 | 0.1428 | 20602 | 0.0664 | 0.0472 | 0.1591 | 20602 | 0.0186 | 0.0467 | 0.69 | 20497 |
| IL6_tertileTop | 0.0931 | 0.0537 | 0.0829 | 20602 | 0.0885 | 0.0544 | 0.1033 | 20602 | <0.0001 | 0.0541 | 0.9176 | 20497 |
| age:IL6_tertileMiddle | <0.0001 | 0.0038 | 0.178 | 20602 | <0.0001 | 0.0038 | 0.1957 | 20602 | <0.0001 | 0.0038 | 0.2233 | 20497 |
| age:IL6_tertileTop | <0.0001 | 0.0043 | 0.2749 | 20602 | <0.0001 | 0.0043 | 0.315 | 20602 | <0.0001 | 0.0043 | 0.2901 | 20497 |
| age^2 (acceleration):IL6_tertileMiddle | 0.0001 | <0.0001 | 0.1184 | 20602 | 0.0001 | <0.0001 | 0.1243 | 20602 | 0.0001 | <0.0001 | 0.1339 | 20497 |
| age^2 (acceleration):IL6_tertileTop | 0.0002 | <0.0001 | 0.0638 | 20602 | 0.0002 | <0.0001 | 0.0713 | 20602 | 0.0002 | <0.0001 | 0.0533 | 20497 |
| SexFemale | - | - | - | - | 0.0917 | 0.0117 | <0.0001 | 20602 | 0.1229 | 0.0117 | <0.0001 | 20497 |
| smoking_statusNever | - | - | - | - | - | - | - | - | <0.0001 | 0.0222 | <0.0001 | 20497 |
| smoking_statusPrevious | - | - | - | - | - | - | - | - | <0.0001 | 0.0229 | <0.0001 | 20497 |
| Townsend | - | - | - | - | - | - | - | - | 0.0979 | 0.0069 | <0.0001 | 20497 |
| BMI | - | - | - | - | - | - | - | - | 0.0765 | 0.0064 | <0.0001 | 20497 |
| Intercept variance | 0.8359 | 0.9143 | - | - | 0.9055 | 0.9516 | - | - | 0.8188 | 0.9049 | - | - |
| Age (slope) variance | 0.0008 | 0.0276 | - | - | 0.0009 | 0.0292 | - | - | 0.0008 | 0.0278 | - | - |
| Intercept/age covariance | -0.0164 | -0.6493 | - | - | -0.0189 | -0.6812 | - | - | -0.0166 | -0.6594 | - | - |
| Residual variance | 0.5852 | 0.765 | - | - | 0.5808 | 0.7621 | - | - | 0.5846 | 0.7646 | - | - |
| Deviance | 222893 |  |  |  | 222835 |  |  |  | 221177 |  |  |  |
| AIC | 222973 |  |  |  | 222917 |  |  |  | 221267 |  |  |  |
| BIC | 223346 |  |  |  | 223299 |  |  |  | 221686 |  |  |  |

Supplementary Material References

[1] Boyd A, Golding J, Macleod J, Lawlor DA, Fraser A, Henderson J, et al. Cohort Profile: The ‘Children of the 90s’—the index offspring of the Avon Longitudinal Study of Parents and Children. International Journal of Epidemiology. 2013;42(1):111-27. <https://doi.org/10.1093/ije/dys064>.

[2] Fraser A, Macdonald-Wallis C, Tilling K, Boyd A, Golding J, Davey Smith G, et al. Cohort Profile: The Avon Longitudinal Study of Parents and Children: ALSPAC mothers cohort. International Journal of Epidemiology. 2013;42(1):97-110. <https://doi.org/10.1093/ije/dys066>.

[3] Northstone K, Lewcock M, Groom A, Boyd A, Macleod J, Timpson N, et al. The Avon Longitudinal Study of Parents and Children (ALSPAC): an update on the enrolled sample of index children in 2019. Wellcome Open Res. 2019;4:51. <https://doi.org/10.12688/wellcomeopenres.15132.1>.

[4] Harris PA, Taylor R, Thielke R, Payne J, Gonzalez N, Conde JG. Research electronic data capture (REDCap)--a metadata-driven methodology and workflow process for providing translational research informatics support. J Biomed Inform. 2009;42(2):377-81. <https://doi.org/10.1016/j.jbi.2008.08.010>.

[5] Fry A, Littlejohns TJ, Sudlow C, Doherty N, Adamska L, Sprosen T, et al. Comparison of Sociodemographic and Health-Related Characteristics of UK Biobank Participants With Those of the General Population. Am J Epidemiol. 2017;186(9):1026-34. <https://doi.org/10.1093/aje/kwx246>.

[6] World Health Organization. A healthy lifestyle - WHO recommendations, <https://www.who.int/europe/news-room/fact-sheets/item/a-healthy-lifestyle---who-recommendations>; 2010 [accessed 01/03/2024.
